# Total Synthesis of (−)-Bipolarolide D

**DOI:** 10.1021/jacsau.4c00680

**Published:** 2024-10-09

**Authors:** Gleb A. Chesnokov, Julia Friedli, Francis J. Carta, Karl Gademann

**Affiliations:** Department of Chemistry, University of Zurich, Winterthurerstrasse 190, CH-8057 Zurich, Switzerland

**Keywords:** total synthesis, sesteterpenoids, tetraquinanes, Pauson−Khand reaction, Rautenstrauch cycloisomerization

## Abstract

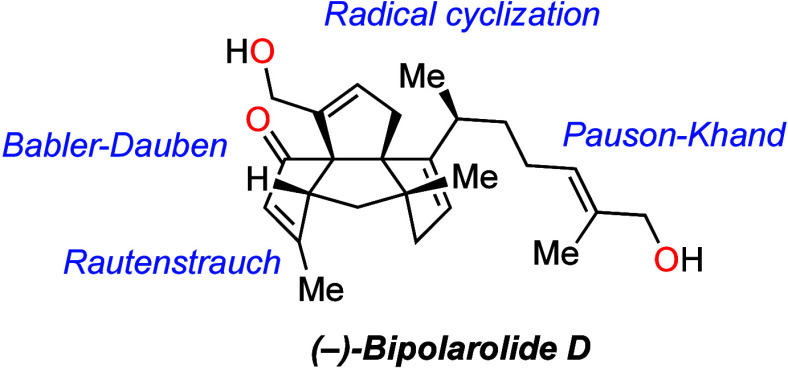

(−)-Bipolarolide D is an ophiobolin-derived sesteterpenoid with a unique tetraquinane (5/5/5/5) tetracyclic skeleton decorated with a diverse set of functionalities. Herein we report a robust, scalable, and efficient total synthesis of this natural product in 1.8% overall yield. The developed approach features a diastereoselective Pauson–Khand reaction, a highly efficient Rautenstrauch cycloisomerization, and radical cyclization to forge the carbon backbone and the installation of the side chain via crotylation with 1-methyl-2-propenylmagnesium chloride followed by Suzuki cross-coupling.

Terpenoids are one of the most abundant classes of secondary metabolites, with diverse structural motifs resulting in a broad variety of biological activities. Quinanes constitute a terpenoid subclass and are formed by the fusion of cyclopentane rings. Linear^1^ and angular triquinanes have been of interest to synthetic chemists since the middle of the 20th century.^2^ In contrast, naturally occurring tetraquinanes were first discovered only in 1985, with just one family of the crinipellins being known until recently.^3^ In 2019 Zhang and co-workers characterized novel examples of tetraquinanes, namely, the sesteterpenoids bipolarolide C (**1**) and D (**2**), together with other natural products with unprecedented structures, derived from ophiobolin precursors.^4^

Bipolarolide D (**2**) possesses an unprecedented 5/5/5/5 tetraquinane carbon backbone decorated with a side chain and containing an isolated stereocenter at C(15) (Figure 1). Within the tetraquinane core, bipolarolide D accommodates four contiguous stereocenters, three of which are quaternary. There are also several functional groups embedded into the structure: an enone in the C ring, an allylic alcohol in the D ring, and an isolated double bond in the A ring. The carbon framework of bipolarolide D includes one linear and two angular triquinane motifs. All these features render bipolarolide D a synthetically challenging yet captivating and inspiring target.

**Figure 1:**
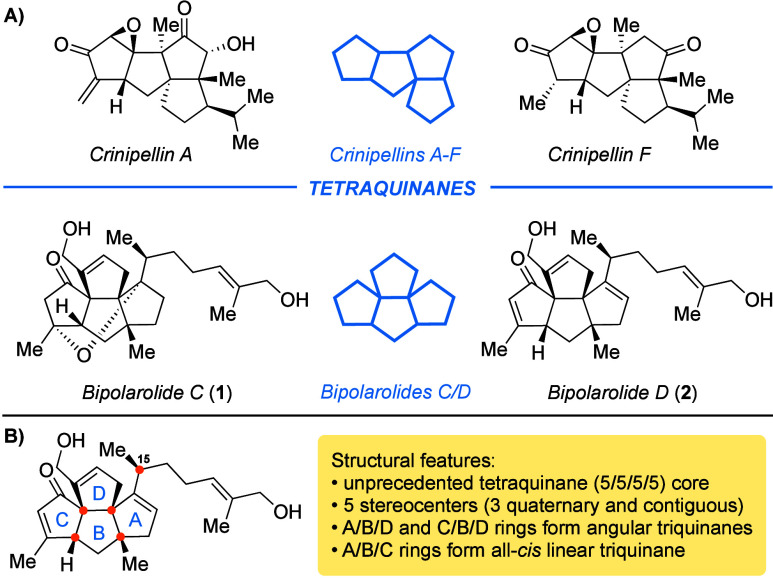
(A) Overview of natural tetraquinane classes. (B) Overview of bipolarolide D.

When the experimental work on this project was nearing completion, a total synthesis of bipolarolide D was published independently by Lu and co-workers.^5^ Here we report an alternative approach allowing for a scalable preparation of >100 mg of the title compound in quadrupled overall yield.

Retrosynthetic analysis (Scheme 1) starts with installation of the side chain by 1,2-addition to the corresponding ketoenone **3** with subsequent dehydration of the intermediate tertiary alcohol. The α,β-enone functionality in the C ring might be installed via Babler–Dauben oxidative 1,3-transposition, and the D ring can be closed in turn by radical cyclization, leading to compound **4**. It was envisioned that the cyclopentenone moiety in linear triquinane **4** can be assembled via a Au(I)-catalyzed Rautenstrauch cycloisomerization, which can be traced back to enyne **5**. The alkyne moiety in **5** suggests Sonogashira cross-coupling, the side chain might be installed via Cu(I)-mediated 1,4-addition, and the bicyclo[3.3.0]octane carbon framework of **5** was envisaged to be constructed via a Pauson–Khand reaction starting from literature-known alcohol **6**.^6^

**Scheme 1:**
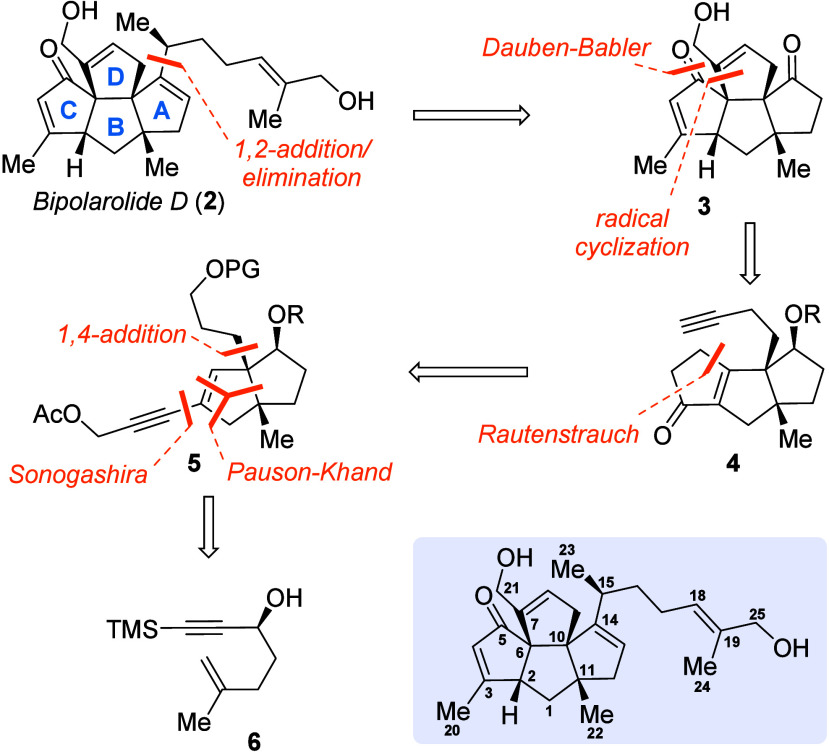
Retrosynthetic Analysis of Bipolarolide D

The synthesis commenced with the preparation of the literature-known alcohol **6** from commercially available *N*-methoxy-*N*,4-dimethyl-4-pentenamide via Li TMS-acetylenide addition followed by Noyori transfer hydrogenation, delivering (*S*)-**6** in 92% yield with 99.7% *ee*. The absolute configuration of compound **6** was confirmed by Mosher ester analysis.^7^ Protodesilylation of the TMS group in **6** was achieved by treatment with K_2_CO_3_ in wet MeOH, and the hydroxy group was protected with *p*-methylbenzyl (MBn), delivering the starting material for the Pauson–Khand reaction **7** in 87% yield (Scheme 2). Screening of various conditions (see Table SI-1) for the Pauson–Khand cyclization of enyne **7** identified utilization of an equimolar amount of Co_2_(CO)_8_ together with heating in MeCN as crucial for the success of the transformation.^8^ Unfortunately, the performance of the catalytic system was inferior. Thus, under the optimized conditions, enyne **7** was converted to the corresponding bicyclic enone **8** in 66% yield with 3.1:1 *dr* at C(11).

**Scheme 2:**
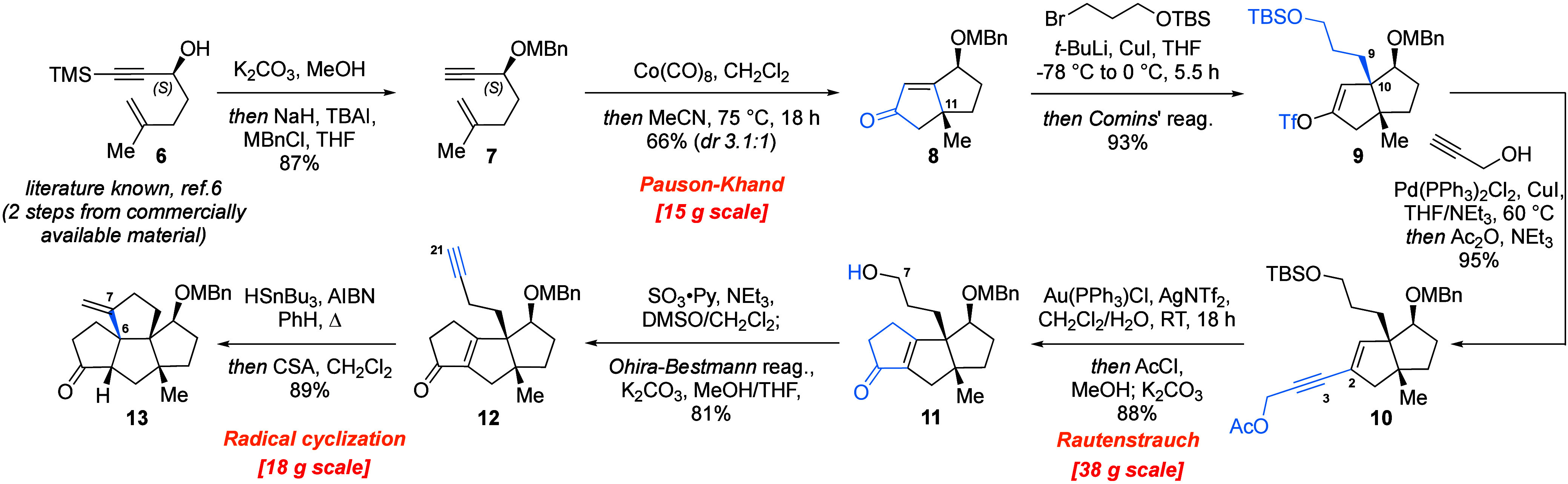
Synthesis of the Tetraquinane Core (**13**) of Bipolarolide D

The desired diastereomer was then subjected to a Cu(I)-mediated 1,4-addition. Testing of different Cu(I) sources (CuI, CuCN, CuBr·DMS, and PrCCCu) demonstrated superior performance of a CuI-based lithium cuprate. When compound **8** was treated with lithium dialkylcuprate, prepared *in situ* from (3-bromopropoxy)-*tert*-butyldimethylsilane, and the intermediate Cu enolate was trapped with Comins’ reagent, vinyl triflate **9** was directly obtained in 93% yield. As expected based on their energy difference, only the *cis*-fused bicyclo[3.3.0]octane product **9** was observed. Vinyl triflate **9** was then subjected to cross-coupling with propargyl alcohol under Sonogashira conditions, and the intermediate product was directly acetylated with acetic anhydride, resulting in enyne **10** in 95% yield (Scheme 2).

With enyne **10** in hand, the stage was set for the Rautenstrauch cycloisomerization. Delightfully, already the first attempt of the transformation with (Ph_3_P)AuCl as the precatalyst and AgSbF_6_ as the activator in DCM at room temperature successfully installed the desired cyclopentenone moiety in **11** in 72% overall yield.^9^ It is noteworthy that the reaction results in three products: in addition to the desired hydroxyenone **11**, the TBS- and Ac-protected analogues of the OH group at C(7) were obtained. The ratio of these products was not very well reproducible, but the combined yield remained consistent. Further screening of solvents (e.g., THF, acetone, toluene; see Table SI-2) and Ag-based activators (AgNTf_2_, AgBF_4_, AgOTf) identified AgNTf_2_ as the most potent activator and wet DCM as the best reaction medium. It is important to mention that addition of water turned out to be crucial for smooth reaction performance, presumably due to facilitation of proton-transfer events.^10^ Under the optimized conditions, (Ph_3_P)AuCl was activated with AgNTf_2_, followed by addition of enyne **10** in DCM and H_2_O. The resulting mixture of three products was then directly submitted to desilylation and methanolysis conditions to cleanly deliver the desired linear triquinane **11** in 88% overall yield. Interestingly, no product with the less substituted C(4)=C(5) double bond was observed. It is noteworthy that the Rautenstrauch cycloizomerization step was performed on 38 g of **10**, showcasing the robustness and efficiency of the procedure.

Next, the free OH group at C(7) in **11** was first oxidized to the corresponding aldehyde under Parikh–Doering conditions,^11^ followed by Seyferth–Gilbert homologation^12^ with the Ohira–Bestmann reagent^13^ to obtain alkyne **12**, which set the stage for closure of the D ring of bipolarolide D (**2**). The forging of the D ring of bipolarolide D was performed under radical conditions with tributyltin hydride in the presence of AIBN as a radical initiator. The crude vinylstannane was protodestannylated with CSA in DCM, affording the tetraquinane core **13** of bipolarolides C and D in 89% yield. It should be highlighted that more than 10 g of intermediate **13** was prepared in one run. Then tetraquinane intermediate **13** was oxidized via the catalytic Saegusa–Ito protocol to install α,β-enone functionality in **14** in 86% yield^14^ and set a handle for further Babler–Dauben oxidative 1,3-transposition (Scheme 3). The next challenge was the installation of an allylic alcohol at C(8)–C(7)–C(21). First, the isolated double bond (C(7)=C(21)) in **14** was epoxidized with *m*-CPBA almost quantitatively, giving a 3.7:1 mixture of diastereoisomeric epoxides, which was in turn treated with MeLi in Et_2_O to diastereoselectively furnish the Me group at C(3) in compound **15**. Interestingly, the spatial architecture of the molecule guided the 1,2-addtion exclusively from the convex face of the linear triquinane moiety of the A/B/C ring system. With epoxide **15** in hand, isomerization of the epoxide to the corresponding allylic alcohol **16** was investigated. Extensive screening of both acidic and basic reagents (see Table SI-3) revealed ambiphilic Et_2_AlTMP to be the optimal reagent for the purpose.^15^ Thus, slow warming of **15** in toluene in the presence of Et_2_AlTMP from −78 to −20 °C allows for smooth installation of the desired allylic alcohol functionality in **18** in 74% yield. Interestingly, in case of acidic conditions (TMSOTf/2,6-lutidine), the occurrence of a Meinwald rearrangement could be postulated based on the presence of aldehydes in the reaction mixture, as evidenced by NMR spectroscopy.

**Scheme 3:**
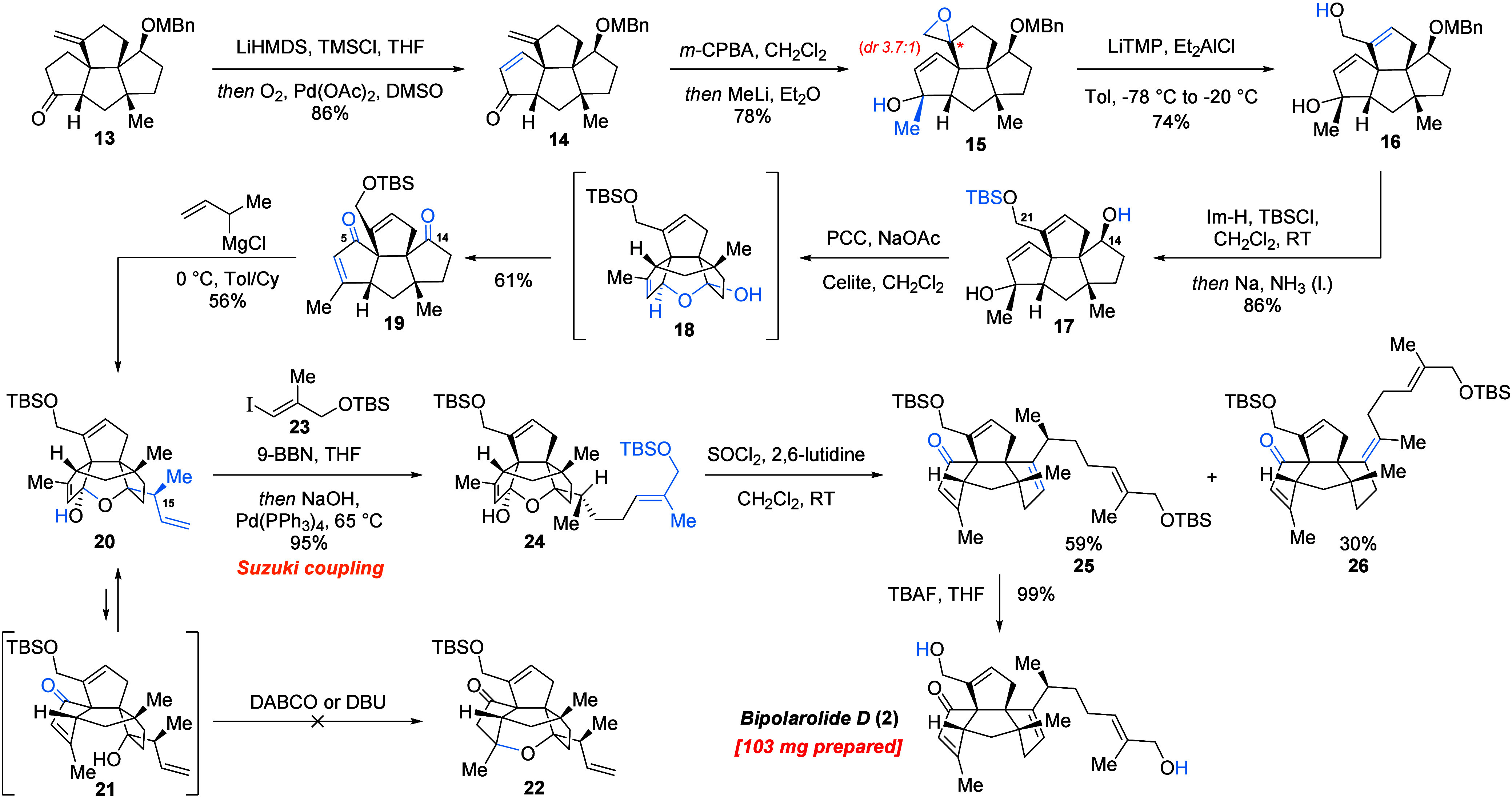
Decoration of the Core and Completion of the Total Synthesis of (−)-Bipolarolide D

Then, to set the stage for Babler–Dauben oxidative 1,3-transposition, the allylic OH group at C(21) was protected with TBSCl/Im-H and the MBn protecting group was removed from the hydroxy group at C(14) under Birch conditions to give **17** in 86% yield. Originally, the MBn group removal was planned with DDQ,^16^ but unfortunately, this reagent induced oxidation of the TBS allylic ether at C(21), forming the corresponding aldehyde. Babler–Dauben reaction with simultaneous oxidation of the secondary alcohol at C(14) was then screened (see the Supporting Information (SI)).^17^ Accounting for the acid-lability of the tertiary alcohol at C(3) and mild acidity of Cr(VI) reagents, all the screened conditions were buffered with mild bases. It was eventually found that PDC/NaOAc was not reactive enough to induce the desired transformation, performing only oxidation of the secondary alcohol at C(14). An interesting observation was made when the PCC/NaOAc system was tested: oxidation of the secondary OH group is fast and is done within 10 min (see Table SI-4); then 1,3-transposition happens within the next 1–2 h, and the intermediate transposed alcohol is then intramolecularly trapped by the newly formed carbonyl group at C(14) to form the corresponding hemiacetal **18**, which can be isolated in up to 63% yield (see the SI). If the reaction mixture is allowed to stir overnight, conversion to the desired product **19** is induced in 61% yield. Collins reagent was also tested and showed the highest reactivity (formation of the desired product **19** in less than 1 h), but due to relatively high acidity, a considerable amount of tertiary alcohol elimination product was isolated (see Table SI-4). Formation of the hemiacetal was also supportive for the plan of trying the 1,2-addition on ketoenone **19** without any concern of overaddition to the enone moiety in the C ring, since the forming tertiary alcohol at C(14) is to be trapped by the enone with the formation of the corresponding hemiacetal at C(5).

The 1,2-addition to ketoenone **19** proved to be rather challenging (Scheme 3). First, isopropyl-based organometallic reagents (*i*-PrMgCl, *i*-PrLi, *i*-PrMgCl/LaCl_3_·2LiCl) as models for the side chain were tested under various conditions, yet without any trace of the desired product formed. Better results were obtained with MeMgBr/LaCl_3_·2LiCl: the expected product was isolated in 55% yield. In light of these results, it was decided to assemble the side chain sequentially, first trying to perform crotylation of the carbonyl group at C(14), since this transformation should be more facile than 1,2-addition of secondary organometallics. Allylic Grignard reagents are known to be more reactive,^18^ and delightfully already the first try with 1-methyl-2-propenylmagnesium chloride in THF showed the formation of three new products and full consumption of the starting material (see the SI). Further screening of the conditions allowed for isolation of the desired crotylated product **20** in 56% yield when the reaction was performed in a toluene/cyclohexane mixture at 0 °C. The structure of hemiacetal **20** was confirmed by 2D NOESY NMR experiments. It is important to mention that polar solvents (THF) favor formation of the product of formal γ-addition of 1-methyl-2-propenylmagnesium chloride to the carbonyl group in **19**, whereas apolar solvents (toluene and cyclohexane), on the contrary, favor formation of the products of formal α-addition. Here an important structural and biogenetic connection must be noted. Transposition of the ether bridge between C(5) and C(14) in **20** from C(5) to C(3) would generate the pentacyclic structure **22** of bipolarolide C. This transposition can be seen as an equilibration between products of 1,2- and 1,4-addition of the tertiary alcohol at C(14) to the enone in the C ring in intermediate **21**. Unfortunately, screening of different bases (DBU, DABCO, etc.) did not result in any trace of rearranged product, presumably due to much higher strain energy of the bipolarolide C backbone, suggesting the need for an alternative retrosynthesis to this target.

With crotylated product **20** in hand, the side chain was then attached to the vinyl group via Suzuki coupling. Hemiacetal **20** was treated with 9-BBN, and the resulting trialkylborane was then coupled with vinyl iodide **23**, following Suzuki’s protocol,^19^ to obtain the side-chain-elongated product **24** in 95% yield (Scheme 3). Then the hemiacetal in **24** was broken in order to install the C(13)=C(15) double bond. First, standard conditions for tertiary alcohol dehydration were tested (SOCl_2_, Py, 0 °C) and afforded the desired compound **25** together with isomerized byproduct **26** with a C(14)=C(15) double bond; however, the conversion was not complete. When 2,6-lutidine was used instead, a fast reaction was observed, and the mixture of the same two products was obtained, from which the desired product was isolated in 59% yield. Unfortunately, further screening under these conditions was not fruitful. Presumably, the conformer with an *anti*-periplanar configuration between the hydrogen at C(15) and the oxygen atom of the ether bridge, being the most stable, is responsible for the ease of the byproduct formation. The dehydrated product **25** was then quantitatively deprotected with TBAF to afford totally synthetic bipolarolide D (**2**). It is noteworthy that >100 mg of (−)-bipolarolide D was prepared in one run. NMR spectra of the synthetic bipolarolide D were in agreement with those reported in the isolation study. The specific rotation for the totally synthetic sample was found to be higher in absolute value (−89.1 vs −51) than that for the isolated natural product, suggesting that the isolated bipolarolide D was only enantioenriched. When comparing this approach to the concomitant study of Lu et al.,^5^ their approach resulted in preparation of the natural product in 0.4% overall yield starting from commercially available methyl dimethoxyacetate. While the overall number of steps is significant for both syntheses, the results outlined in this study improve both the overall yield by a factor of 4 and the scalability of the overall synthetic sequence.

In conclusion, a robust, scalable, and efficient total synthesis of (−)-bipolarolide D (**2**) was achieved in 1.8% overall yield starting from commercially available *N*-methoxy-*N*,4-dimethyl-4-pentenamide. Salient features of this approach include (1) sequential formation of the four five-membered rings of the bipolarolide D backbone, (2) a diastereoselective Pauson–Khand reaction, (3) the highly efficient Rautenstrauch cycloisomerization on a large scale, (4) a radical cyclization to forge the D ring, and (5) installation of the side chain via crotylation with 1-methyl-2-propenylmagnesium chloride followed by Suzuki cross-coupling. Also, the viability of accessing the correspondent scaffold of bipolarolide C was briefly investigated via rearrangement of hemiacetal **20**. The developed route expands the chemical subspace of tetraquinanes, allowing for further search for novel biological activities.

## Supporting Information Available

Experimental procedures and copies of NMR spectra (PDF)
