# Supplementary material for: Total Synthesis of (−)-Bipolarolide D
Source: JACS Au. 2024 Oct 9;4(11):4194–8. doi: 10.1021/jacsau.4c00680 (PMC11600160; doi:10.1021/jacsau.4c00680)
Supplement: Supplementary file 1 — au4c00680_si_001.pdf [file au4c00680_si_001.pdf]

Total Synthesis of (–)-Bipolarolide D

Gleb A. Chesnokov,[+] Julia Friedli,[+] Francis J. Carta,[+] Karl Gademann[*]

Department of Chemistry, University of Zurich, Winterthurerstrasse 190,

CH-8057 Zurich, Switzerland

Email: karl.gademann@uzh.ch

Supporting Information

Contents

Optimization results 3

Pauson-Khand reaction 3

Rautenstrauch cycloisomerization 4

Epoxide opening 5

Babler-Dauben oxidative 1,3-transposition 6

Grignard reagent addition 7

Experimental procedures and characterization data 8

General information 8

Synthetic procedures 9

Comparison of NMR spectra of natural and synthetic samples of Bipolarolide D 31

^{1}H NMR 31

^{13}C NMR 32

NMR Spectra 33

Optimization results

Table SI-1. *Pauson-Khand* reaction

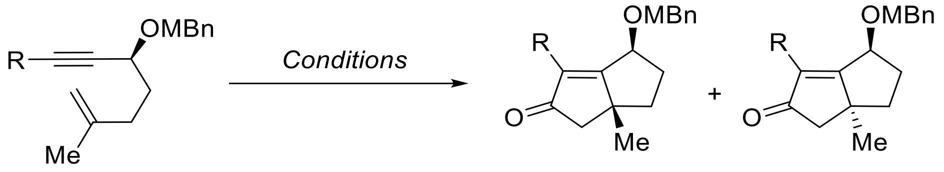

Entry	Substrate	Complex	Additive	Solvent	Temperature	Atmosphere	Result
**1**	R = TMS	$Ni(COD)_2$ (1.1 eq.)	2,2'-bipyr (1.2 eq.)	Toluene	RT	N_2 then CO	-
**2**	R = H	$Ni(COD)_2$ (1.1 eq.)	2,2'-bipyr (1.2 eq.)	Toluene	RT	N_2 then CO	-
**3**	R = H	$[Rh(CO)_2Cl]_2$ (10 mol%)	-	DCE	RT to 60 °C	N_2 then CO	-
**4**	R = H	$Co_2(CO)_8$ (10 mol%)	TMTU (60 mol%)	Toluene	RT to 80 °C	N_2 then CO	SM/product traces
**5**	R = TMS	$Co_2(CO)_8$ (1.2 eq.)	4 Å MS	Toluene	RT to 110 °C	N_2	-
**6**	R = H	$Co_2(CO)_8$ (1.2 eq.)	4 Å MS	Toluene	RT to 110 °C	N_2	product traces
**7**	R = TMS	$Co_2(CO)_8$ (1.2 eq.)	-	DCM, then MeCN	RT to 75 °C	N_2	-
**8**	R = H	$Co_2(CO)_8$ (1.2 eq.)	NMO (10.0 eq.)	DCM	RT	N_2	29% dr: 1.6:1
**9**	R = H	$Co_2(CO)_8$ (1.1 eq.)	-	DCM, then MeCN	RT to 75 °C	N_2	66% dr: 3.1:1

Table SI-2. *Rautenstrauch* cycloisomerization

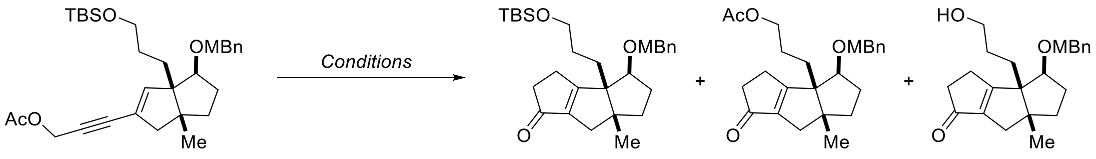

10 11

Entry	Precatalyst	Silver Salt	Solvent	Temperature	Combined Yield
**1**	$(PPh_3)AuCl$ (5 mol%)	$AgSbF_6$ (6 mol%)	dry DCM	rt	72%
**2**	$(PPh_3)AuCl$ (5 mol%)	AgOTf (6 mol%)	dry DCM	rt	72%
**3**	$(PPh_3)AuCl$ (5 mol%)	$AgNTf_2$ (6 mol%)	dry DCM	rt	79%
**4**	$(PPh_3)AuCl$ (5 mol%)	$AgBF_4$ (6 mol%)	dry DCM	rt	60%
**5**	$(PPh_3)AuCl$ (5 mol%)	$AgNTf_2$ (6 mol%)	acetone	rt	50%
**6**	$(PPh_3)AuCl$ (5 mol%)	$AgNTf_2$ (6 mol%)	toluene	rt	SM and product traces
**7**	$(PPh_3)AuCl$ (5 mol%)	$AgNTf_2$ (6 mol%)	THF	rt	SM and product traces
**8**	$(PPh_3)AuCl$ (5 mol%)	$AgNTf_2$ (6 mol%)	wet DCM	rt	88%

Table SI-3. Epoxide opening

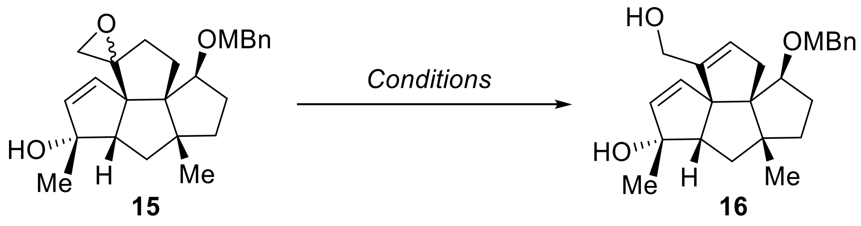

Entry	Reagent	Solvent	Temperature	Result
**1**	TMSOTf, 2,6-lutidine	DCM	0 °C to RT	Complex Mixture
**2**	Al(*i*-PrO)$_3$	Toluene	110 °C	SM
**3**	Ti(*i*-PrO)$_4$	Toluene	110 °C	SM/Product traces
**4**	LDA, KO*t*-Bu	THF	–78 °C to RT	SM/Product traces
**5**	TMPMgCl·LiCl	THF	0 °C to RT	SM
**6**	TMP$_2$Mg·2LiCl	THF	0 °C to RT	Product/Mixture
**7**	Et$_2$AlTMP	Toluene	–78 °C to RT	57% (80% purity)
**8**	Et$_2$AlTMP	Toluene	–50 °C	25%, full consumption of SM
**9**	Et$_2$AlTMP	Toluene	–30 °C	40%, full consumption of SM
**10**	Et$_2$AlTMP	Toluene	–78 °C to –20 °C	74%

Table SI-4. ***Babler-Dauben*** **oxidative 1,3-transposition**

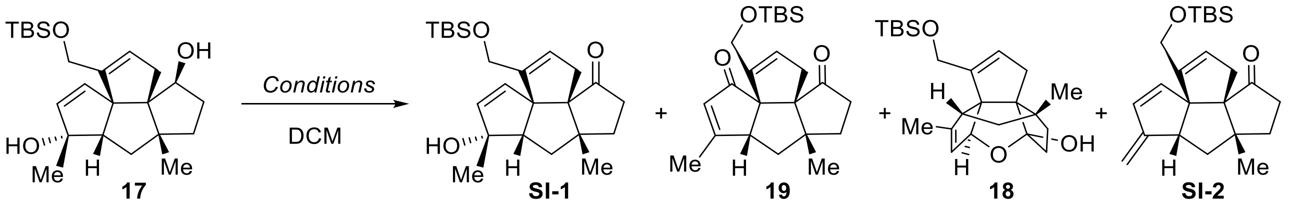

Entry	Reagent	Temperature Time	SI-1, %	19, %	18, %	SI-2, %
**1**	PCC (4 eq.) NaOAc (4 eq.)	0 °C, 3 h	0	0	63	7
**2**	PCC (8 eq.) NaOAc (10 eq.)	RT, 2 h	0	38	21	6
**3**	PDC (10 eq.) NaOAc (10 eq.)	RT, 2 h	36	0	12	0
**4**	PCC (8 eq.) Al_2O_3 (basic)	RT, 2 h	0	46	0	18
**5**	CrO_3 (12 eq.) Py (24 eq.)	RT, 2 h	0	32	0	17
**6**	PCC (12 eq.), Celite NaOAc (14 eq.)	RT, 18 h	0	61	0	0

Table SI-5. *Grignard* reagent addition

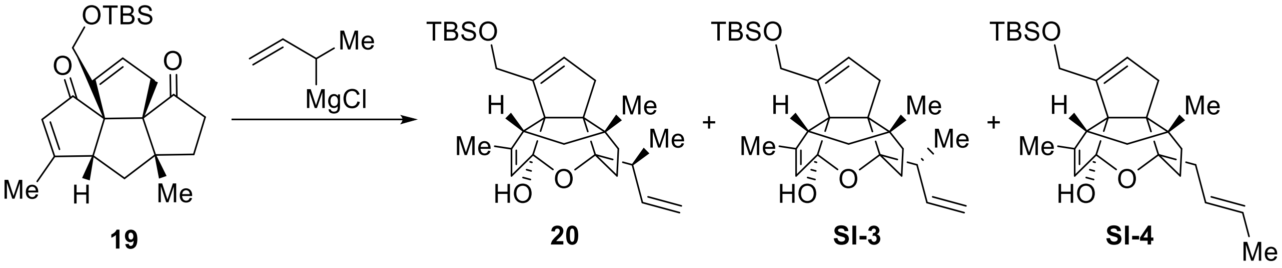

19 **20** **SI-3** **SI-4**

Entry	Additive	Solvent	Temperature, °C	20, %	SI-3, %	SI-4, %
**1**	$CeCl_3$	THF	0	21	24	42
**2**	-	THF	0	24	24	41
**3**	-	Toluene	0	48	22	18
**4**	-	Toluene	-78	40	33	15
**5**	-	Toluene/ Cyclohexane	0	56	26	12

Experimental procedures and characterization data

General information

All chemicals have been purchased from Acros, Fluka, Fluorochem, or Sigma-Aldrich and were used without further purification. All reactions have been carried out in heat gun-dried glassware and under an atmosphere of nitrogen, unless otherwise stated. Dry solvents were purchased from commercial sources. Solvents for work-up and purification have been distilled from technical grade. All synthetic transformations have been monitored by thin layer chromatography (TLC). Yields refer to purified, dried and spectroscopically pure compounds. TLC was performed on Merck aluminum silica gel 60 F_{254} plates (0.25 mm thickness) pre-coated with a fluorescent indicator, and visualized by staining with I_2 vapors, Cerium Ammonium Molybdate solution (CAM), or $KMnO_4$ stain. Concentration under reduced pressure was performed by rotary evaporation at 40°C. Flash column chromatography was performed using silica gel 60 (230-400 mesh) from Sigma-Aldrich with a forced flow eluent at 0.1-0.5 bar pressure. All NMR spectra were recorded using a Bruker Avance (400 MHz or 500 MHz) spectrometers at room temperature (unless otherwise stated). Chemical shifts (δ-values) are reported in ppm, spectra were calibrated relative to the residual proton chemical shifts ($CHCl_3$, δ = 7.26; DMSO-d_6, δ = 2.50; MeOH-d_4, δ = 4.87; DCM, δ = 5.32) and carbon chemical shifts ($CDCl_3$, δ = 77.16; DMSO-d_6, δ = 39.52; MeOH-d_4, δ = 49.0) of the solvents, multiplicity is reported as follows: s = singlet, d = doublet, t = triplet, q = quartet, m = multiplet or unresolved and coupling constant *J* in Hz. IR spectra were recorded on a Perkin Elmer SpectrumTwo ATR-FTIR. The absorptions are reported in cm^{-1}. All mass spectra (HRMS) were recorded by the Mass Spectrometric Service of the University of Zürich on a QExactive instrument (Thermo Fisher Scientific, Bremen, Germany) equipped with a heated electrospray (ESI) or an atmospheric pressure chemical ionization (APCI) ionization source and connected to a Dionex Ultimate 3000 UHPLC system. Melting points (MP) were determined using a Büchi B-545 apparatus in open capillaries and are uncorrected. Optical rotations $[\alpha]_D$ were measured at the sodium D line using a 2 ml cell with a 1 dm path length or a 0.15 ml cell with a 0.1 dm path length on a Jasco P-2000 digital polarimeter and the concentrations c are given in g/100 ml.

Synthetic procedures

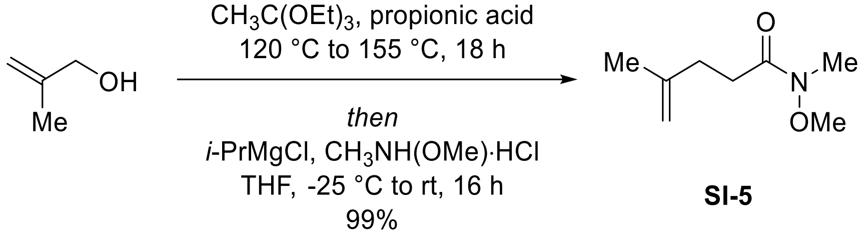

SI-5

A round-bottom flask, equipped with a *Vigreux* column and a distillation bridge, was charged with methallyl alcohol (26.7 mL, 22.9 g, 318 mmol, 1.00 eq.), triethyl orthoacetate (167 mL, 103 g, 636 mmol, 2.00 eq.) and propionic acid (0.31 mL, 306 mg, 4.13 mmol, 0.013 eq.). The solution was heated for 1 hour at 120 °C, then for 1 h at 130 °C and for 1 h at 140 °C. Additional propionic acid (0.31 mL, 306 mg, 4.13 mmol, 0.013 eq.) was added and the solution was heated at 155 °C overnight (15 h). The reaction mixture was cooled down to 0 °C and HCl (0.5 M, 125 mL) was slowly added. After 2 h, GC-MS showed no presence of triethyl orthoacetate. DCM (250 mL) was added, and the layers were separated. The aqueous phase was extracted with DCM (3 × 100 mL) and the combined organic layers were dried over Na_2SO_4, filtered and concentrated (40 °C, 150 mbar). The residue was dissolved in THF (700 mL) and *N,O*-dimethylhydroxylamine hydrochloride (34.1 g, 350 mmol, 1.00 eq.) was added. The suspension was cooled down to –25 °C and *i*-PrMgCl (350 mL, 700 mmol, 2 M in THF, 2.20 eq.) was added with a dropping funnel over 1 h. After addition was complete, the reaction mixture was left stirring overnight at RT. The reaction mixture was cooled down to 0 °C and quenched with sat. aq. NH_4Cl soln. (800 mL). The layers were separated, and the aqueous phase was extracted with Et_2O (4 × 400 mL). The combined organic layers were washed with brine (800 mL), dried over Na_2SO_4, filtered, and concentrated to give **SI-5** (49.6 g, 315 mmol, 99%)

^{1}H-NMR (400 MHz, $CDCl_3$): δ 4.75 – 4.73 (m, 1H), 4.71 – 4.69 (m, 1H), 3.69 (s, 3H), 3.18 (s, 3H), 2.57 (dd, *J* = 9.5, 6.3 Hz, 2H), 2.37 – 2.30 (m, 2H), 1.76 (d, *J* = 0.6 Hz, 3H).

The spectroscopic data agree with that reported.[1]

[1] G. C. Tay, N. Sizemore, S. D. Rychnovsky, *Org. Lett.* **2016**, *18*, 3050–3053.

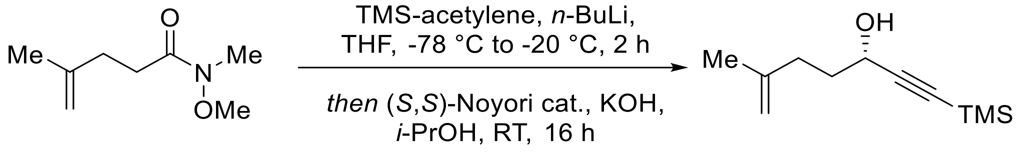

SI-5 **6**

To a solution of TMS-acetylene (55.5 mL, 38.3 g, 390 mmol, 1.30 eq.) in THF (1.4 L) at –78 °C *n*-BuLi (11 M in *n*-hexane, 32.7 mL, 360 mmol, 1.20 eq.) was slowly added and the solution was left to stir for 1 h, after which **SI-5** (47.2 g, 300 mmol, 1.00 eq.) in dry THF (100 mL) was added via cannula. The pale-yellow solution was stirred at this temperature for 30 min. It was allowed to warm up to –20 °C over 30 min and it was stirred at this temperature for 1 h more, after which TLC showed full consumption of the starting material. The solution was then slowly cannulated into a 5 L beaker filled with ice-cold 2M HCl (3 L) under vigorous stirring. Et_2O (1 L) was added and the organic phase was separated. The aqueous phase was extracted with Et_2O (3 × 750 mL). All organic fractions were washed with PBS (3 × 1 L) until a neutral pH was obtained. The combined organic phase was washed once with brine (1 L), dried over anhydrous Na_2SO_4, and concentrated under reduced pressure. (*S*,*S*)-*Noyori* cat. (RuCl[(1*S*,2*S*)-TsDPEN](p-cymene)) (5.00 g, 7.92 mmol, 2.60 mol%), was dissolved in DCM (60 mL) and finely powdered KOH (0.89 g, 15.8 mmol, 5.20 mol%) was added. After 10 min, under vigorous stirring, H_2O (45 mL) was added, and a color change from dark orange to an intense purple was observed. After 10 min, the organic layer was transferred into a second flask via cannula and the aqueous phase was washed once with DCM. Powdered CaH_2 was added to the catalyst solution and the mixture was stirred until the gas evolution ceased. The organic layer was filtered by cannula and concentrated under N_2 until ca. 30 mL of DCM remained to give a solution of the activated *Noyori* catalyst. The crude intermediate product was redissolved in freshly distilled *i*-PrOH (1.5 L) and the activated *Noyori* cat. in DCM was added. The initially purple solution quickly turned orange and it was left to stir overnight (16 h), after which full consumption of starting material was observed according to TLC. The solution was concentrated and the crude mixture was purified by flash column chromatography (*n*-hexane/EtOAc 8:1) to give pure **6** (54.4 g, 277 mmol, 92%, 99.7% ee).

N.B. During lithium TMS-acetylenide addition it is not recommended to allow the solution to warm up above -20 °C as decomposition can occur.

Appearance: yellow oil.

R_f (SiO_2, *n*-hexane/EtOAc 10:1, $KMnO_4$): 0.27.

FTIR (thin film, neat, cm^{-1}): 3334, 3076, 2959, 2173, 1651, 1448, 1376, 1251, 1072, 1021, 950, 888, 843, 760, 700, 649, 429.

^{1}H-NMR (400 MHz, $CDCl_3$) δ 4.76 – 4.72 (m, 2H), 4.38 (td, *J* = 6.5, 5.6 Hz, 1H), 2.21 – 2.16 (m, 2H), 1.88 – 1.81 (m, 3H), 1.76 – 1.73 (m, 3H), 0.17 (s, 9H).

$^{13}C\{^1H\}$ NMR (101 MHz, CDCl3) δ 145.2, 110.7, 106.6, 89.8, 62.7, 35.7, 33.4, 22.6, 0.0.

HRMS (ESI, *m/z*): calc. for $C_{11}H_{20}OSi$ $[M+H]^+$: 197.1356, found: 197.1356.

Optical Rotation: $[\alpha]_D^{25}$ = +7.7 (*c* 0.475, $CHCl_3$).

Enantiomeric excess was determined by GC-MS (MEGA-DEX DET Beta, 0.25 µm, 0.25 mm, 30 m):

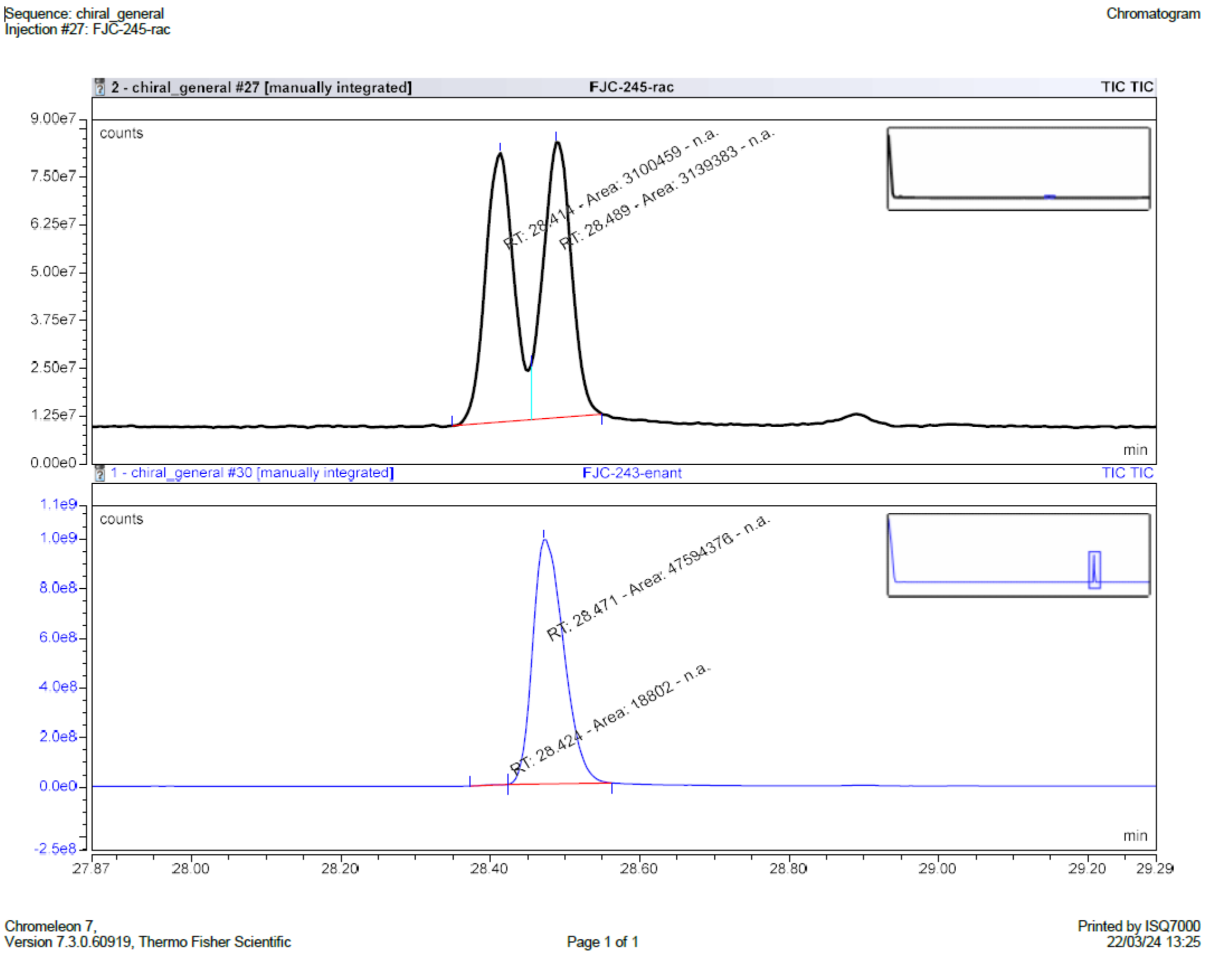

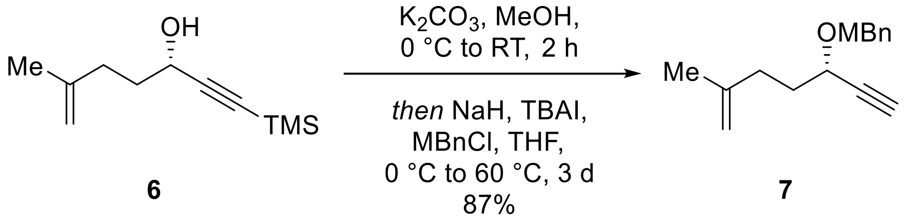

To a solution of alcohol **6** (54.1 g, 276 mmol, 1.00 eq.) in MeOH (1.4 L) at 0 °C K_2CO_3 (45.7 g, 331 mmol, 1.20 eq.) was added and the mixture was warmed up to RT. After 2 h, the mixture was concentrated (35 °C, 150 mbar) until a minimal amount of MeOH was still present. Then, DCM (1 L) and H_2O (1 L) were added and the organic phase was separated. The aqueous phase was extracted with DCM (3 × 300 mL) and the combined organic phase was dried over anhydrous Na_2SO_4, and concentrated (35 °C, 150 mbar). *N.B. This compound is volatile.* The crude mixture in THF (80 mL) was then added to a suspension of NaH (60 w% in mineral oil, 13.2 g, 331 mmol, 1.20 eq.) and TBAI (10.2 g, 27.6 mmol, 0.10 eq.) in dry THF (600 mL) at 0 °C. The reaction was stirred at 0 °C for 30 min, after which MBnCl (43.8 mL, 46.5 g, 331 mmol, 1.20 eq.) was added. The suspension was allowed to warm up to RT and stirred for 15 h. It was then heated to 60 °C for 1 h, after which it was cooled down to 0 °C and quenched with sat. aq. NH_4Cl solution (800 mL). The organic phase was separated and the aqueous phase was extracted with Et_2O (3 × 300 mL). The combined organic phase was washed with brine (600 mL), dried over anhydrous Na_2SO_4 and concentrated. The residue was purified by flash column chromatography (*n*-hexane/EtOAc 40:1) to give **7** (59.1 g, 240 mmol, 87%).

Appearance: yellow oil.

R_f (SiO_2, *n*-hexane/EtOAc 40:1, $KMnO_4$): 0.28.

FTIR (thin film, neat, cm^{-1}): 3301, 2968, 2935, 2858, 1650, 1445, 1376, 1335.

^{1}H-NMR (400 MHz, $CDCl_3$) δ 7.26 (d, *J* = 8.0 Hz, 2H), 7.16 (d, *J* = 7.7 Hz, 2H), 4.77 (d, *J* = 11.5 Hz, 1H), 4.71 (ddt, *J* = 2.2, 1.5, 0.7 Hz, 1H), 4.67 (ddt, *J* = 2.8, 2.1, 0.9 Hz, 1H), 4.46 (d, *J* = 11.5 Hz, 1H), 4.08 (td, *J* = 6.5, 2.1 Hz), 2.48 (d, *J* = 2.0 Hz, 1H), 2.35 (s, 3H), 2.24 – 2.14 (m, 2H), 1.99 – 1.81 (m, 2H), 1.72 (dd, *J* = 1.4, 0.8 Hz, 3H).

^{13}C{^{1}H} NMR (101 MHz, $CDCl_3$) δ 145.0, 137.6, 134.9, 129.2, 128.3, 110.4, 83.0, 74.0, 70.6, 68.0, 33.8, 33.3, 22.7, 21.3.

HRMS (ESI, *m/z*): calc. for $C_{16}H_{20}NaO$ $[M+Na]^+$: 251.1406, found: 251.1406.

$[\alpha]_D^{25}$ = –114.6 (c = 0.435, $CHCl_3$).

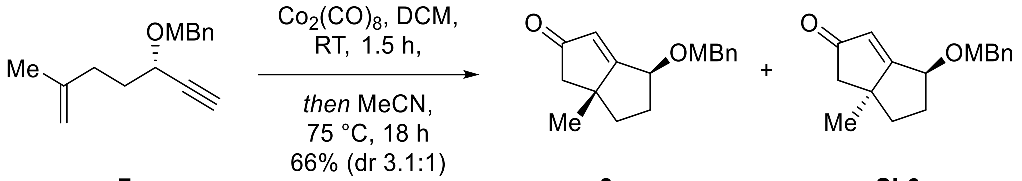

7 **8** **SI-6**

To a suspension of $Co_2(CO)_8$ (25.0 g, 73.1 mmol, 1.10 eq.) in dry DCM (500 mL) alkyne **7** (15.2 g, 66.5 mmol, 1.00 eq.) dissolved in dry DCM (50 mL) was added during which gas evolution was observed. The dark red solution was stirred for 1.5 h and it was then concentrated under N_2. The residue was redissolved in MeCN (1.33 L) and was stirred for 1 h at rt. It was then heated to 75 °C and stirred overnight (18 h). The mixture was cooled down to RT and Celite (400 mL) was added. The mixture was then concentrated under N_2 atmosphere. A minimal amount of DCM was added and the black powder was quickly submitted to flash column chromatography (*n*-hexane/EtOAc 8:1 → 4:1) resulting in full separation of the two diastereomers and to give **8** (8.46 g, 33.0 mmol, 50%) and **SI-6** (2.69 g, 10.5 mmol, 16%).

N.B. After evaporation of MeCN, the black powder is prone to autoignition under normal atmosphere. If heat evolution occurs, a minimal amount of DCM should be added to cool down the powder.

Characterization data for **8**:

Appearance: off-white crystalline solid.

MP: 47.6-48.5 (hexane).

R_f (SiO_2, *n*-hexane/EtOAc 4:1, $KMnO_4$): 0.38.

FTIR (thin film, neat, cm^{-1}): 2954, 2866, 1710, 1631, 1517, 1454, 1411, 1325, 1235, 1174, 1068, 861, 804, 483.

^{1}H-NMR (400 MHz, $CDCl_3$) δ 7.22 (d, *J* = 8.0 Hz, 2H), 7.16 (d, *J* = 7.9 Hz, 2H), 5.95 (s, 1H), 4.57 – 4.51 (m, 2H), 4.43 (d, *J* = 11.3 Hz, 1H), 2.44 (d, *J* = 17.7 Hz, 1H), 2.40 – 2.30 (m, 5H), 2.19 (dddd, *J* = 14.3, 10.2, 8.6, 4.1 Hz, 1H), 2.01 (ddd, *J* = 12.8, 8.6, 2.1 Hz, 1H), 1.45 – 1.34 (m, 4H).

$^{13}C\{^1H\}$ NMR (101 MHz, $CDCl_3$) δ 211.0, 188.2, 137.8, 134.8, 129.3, 128.1, 127.3, 74.7, 71.5, 52.6, 49.3, 35.5, 32.8, 26.0, 21.3.

HRMS (ESI, *m/z*): calc. for $C_{17}H_{20}O_2Na$ $[M+Na]^+$: 279.1356, found: 279.1357.

$[\alpha]_D^{25}$ = +43.4 (c = 0.625, $CHCl_3$).

Characterization data for **SI-6**:

Appearance: off-white crystalline solid.

MP: 57.9-60.1 (hexane).

R_f (SiO_2, *n*-hexane/EtOAc 4:1, $KMnO_4$): 0.28.

FTIR (thin film, neat, cm^{-1}): 2958, 2867, 1707, 1633, 1516, 1454, 1190, 1105, 1053, 805.

^{1}H-NMR (400 MHz, $CDCl_3$): δ 7.25 (d, *J* = 7.4 Hz, 2H), 7.17 (d, *J* = 7.7 Hz, 2H), 6.04 (d, *J* = 1.8 Hz, 1H), 4.82 (ddd, *J* = 9.8, 4.3, 1.9 Hz, 1H), 4.64 (d, *J* = 11.7 Hz, 1H), 4.59 (d, *J* = 11.7 Hz, 1H), 2.52 – 2.40 (m, 1H), 2.37 (s, 2H), 2.35 (s, 3H), 2.00 – 1.84 (m, 2H), 1.77 – 1.67 (m, 1H), 1.15 (d, *J* = 0.7 Hz, 3H).

^{13}C{^{1}H} NMR (101 MHz, $CDCl_3$): δ 209.8, 192.9, 137.9, 134.8, 129.4, 128.0, 123.8, 75.6, 72.3, 52.0, 48.2, 35.7, 30.6, 26.1, 21.3.

HRMS (ESI, *m/z*): calc. for $C_{17}H_{21}O_2$ $[M+H]^+$: 257.1536, found: 257.1532.

$[\alpha]_D^{25}$ = –91.2 (c = 0.435, $CHCl_3$).

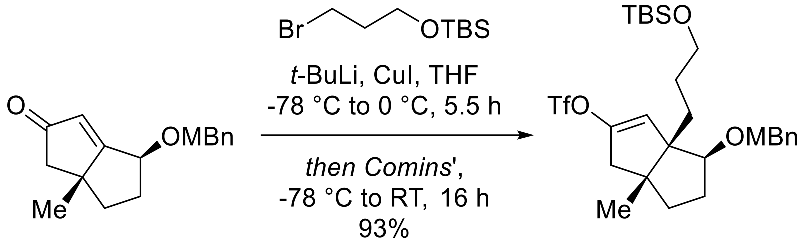

8 **9**

To a solution of (3-bromopropoxy)(*tert*-butyl)trimethylsilane) (75.1 g, 296 mmol, 4.00 eq.) in dry THF (1.2 L) *t*-BuLi (349 mL, 593 mmol, 1.7 M in *n*-pentane, 8.00 eq.) was slowly added with a dropping funnel at –78 °C. After 1 h, CuI (28.2 g, 148 mmol, 2.00 eq.) was added in one portion, resulting in an instant color change from bright yellow to black. The solution was warmed up to 0 °C. Then, enone **8** (19.0 g, 74.1 mmol, 1.00 eq.), dissolved in THF (50 mL), was added via cannula. After stirring for 4.5 h at 0 °C the solution was cooled down to –78 °C and *Comins*' reagent (105 g, 267 mmol, 3.60 eq.) dissolved in THF (200 mL) was added. The solution was allowed to warm up to 0 °C, after which it was warmed up to RT overnight (16 h). The colorless solution was poured into a solution of ice-cold sat. aq. NH_4Cl soln. (1 L). Then, NH_4OH (35%, 100 mL) was added and the solution was vigorously stirred for 1 h under ambient atmosphere. The organic layer was separated and the aqueous phase was extracted with Et_2O (2 × 400 mL). The combined organic phase was washed with brine (800 mL) and dried over anhydrous Na_2SO_4. Celite (500 mL) was added and the solution was concentrated. The blue powder was submitted to flash column chromatography (*n*-hexane/EtOAc 30:1) to give pure **9** (38.9 g, 69.1 mmol, 93%)

Appearance: viscous yellow oil.

R_f (*n*-hexane/EtOAc 1:40, $KMnO_4$): 0.40

FTIR (thin film, neat, cm^{-1}): 2953, 2930, 2857, 1461, 1422, 1247, 1207, 1142, 1095, 1072, 1021, 1006, 939, 904, 832, 802, 774, 661, 611, 569, 518, 481.

^{1}H-NMR (400 MHz, $CDCl_3$) δ 7.20 (d, *J* = 8.0 Hz, 2H), 7.14 (d, *J* = 8.0 Hz, 2H), 5.42 (s, 1H), 4.52 (d, *J* = 11.6 Hz, 1H), 4.28 (d, *J* = 11.6 Hz, 1H), 3.72 (d, *J* = 3.5 Hz, 1H), 3.59 (qt, *J* = 10.0, 7.1 Hz, 2H), 2.60 (dd, *J* = 16.5, 2.2 Hz, 1H), 2.49 (d, *J* = 16.5 Hz, 1H), 2.35 (s, 3H), 1.92 – 1.81 (m, 3H), 1.75 (dd, *J* = 11.7, 8.1 Hz, 1H), 1.62 – 1.42 (m, 3H), 1.31 (td, *J* = 12.8, 4.7 Hz, 1H), 1.15 (s, 3H), 0.89 (s, 9H), 0.04 (s, 6H).

$^{13}C\{^1H\}$ NMR (101 MHz, $CDCl_3$) δ 148.1, 137.1, 136.0, 129.1, 127.5, 123.5, 118.7 (q, *J* = 320.9 Hz), 83.9, 70.8, 64.0, 61.8, 48.1, 47.8, 41.9, 29.1, 28.2, 27.0, 26.1, 26.1, 25.1, 21.3, 18.5, –5.2.

^{19}F-NMR (376 MHz, $CDCl_3$): δ –73.58.

HRMS (ESI, *m/z*): calc. for $C_{27}H_{41}NaO_5F_3SSi$ $[M+Na]^+$: 585.2288, found: 585.2287.

$[\alpha]_D^{25}$ = +13.5 (c = 0.680, $CHCl_3$).

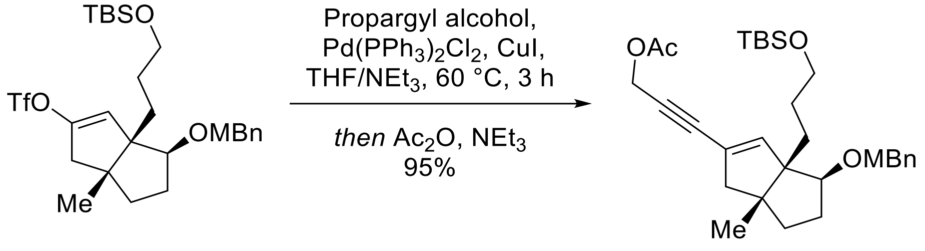

9 **10**

Triflate **9** (47.0 g, 83.6 mmol, 1.00 eq.) was dissolved in THF (220 mL) and NEt_3 (220 mL). Then, propargyl alcohol (18.7 g, 334 mmol, 4.00 eq.), CuI (1.59 g, 8.36 mmol, 0.10 eq.) and $Pd(PPh_3)_2Cl_2$ (2.93 g, 4.18 mmol, 0.05 eq.) were added sequentially. After stirring for 5 min, the suspension was heated to 60 °C and stirred at this temperature for 3 h. The reaction mixture was then cooled down to RT, Et_2O (400 mL) was added and the mixture was filtered through a pad of Celite. Then, sat. aq. NH_4Cl soln. (1 L) was added, and the layers were separated. The aqueous phase was extracted with Et_2O (3 × 800 mL), the combined organic layers were washed with brine (1 L), dried over anhydrous Na_2SO_4 and concentrated. The residue was passed through a pad of silica (*n*-hexane/EtOAc 1:1), concentrated and redissolved in DCM (330 mL). Then, NEt_3 (70.5 mL, 501 mmol, 6.00 eq.), DMAP (3.38 mL, 25.1 mmol, 0.30 eq.) and Ac_2O (25.6 g, 251 mmol, 3.00 eq.) were added sequentially at RT. After 70 min, the reaction was quenched by the addition of sat. aq. NH_4Cl soln. (600 mL). The layers were separated and the aqueous phase was extracted with DCM (3 × 300 mL). The combined organic layers were washed with brine (500 mL), dried over anhydrous Na_2SO_4 and concentrated. The residue was purified by flash column chromatography (*n*-hexane/EtOAc 10:1) to give pure **10** (40.5 g, 79.4 mmol, 95%).

Appearance: colorless oil

R_f: (SiO_2, *n*-hexane/EtOAc 10:1, $KMnO_4$): 0.37

FTIR (thin film, neat, cm^{-1}): 2951, 2931, 2857, 1748, 1517, 1460, 1377, 1358, 1307, 1220, 1093, 1024, 946, 835, 805, 775, 662, 603, 482.

^{1}H-NMR (400 MHz, $CDCl_3$) δ 7.20 (d, *J* = 8.0 Hz, 2H), 7.13 (d, *J* = 7.9 Hz, 2H), 5.84 (s, 1H), 4.80 (s, 2H), 4.50 (d, *J* = 11.7 Hz, 1H), 4.27 (d, *J* = 11.7 Hz, 1H), 3.73 (d, *J* = 3.6 Hz, 1H), 3.56 (qdd, *J* = 9.9, 7.5, 6.3 Hz, 2H), 2.47 (dd, *J* = 16.4, 2.4 Hz, 1H), 2.41 (dd, *J* = 16.3, 1.5 Hz, 1H), 2.34 (s, 3H), 2.11 (s, 3H), 1.85 – 1.72 (m, 3H), 1.66 (dd, *J* = 11.4, 8.3 Hz, 1H), 1.55 – 1.42 (m, 3H), 1.21 (td, *J* = 12.4, 4.7 Hz, 1H), 1.10 (s, 3H), 0.89 (s, 9H), 0.04 (s, 6H).

$^{13}C\{^1H\}$ NMR (101 MHz, $CDCl_3$) δ 170.5, 145.0, 136.9, 136.3, 129.0, 127.4, 123.0, 84.4, 84.2, 84.0, 70.7, 65.8, 64.4, 53.3, 53.1, 49.3, 42.0, 29.2, 28.5, 26.9, 26.2, 25.0, 21.3, 21.0, 18.6, –5.1.

HRMS (ESI, *m/z*): calc. for $C_{31}H_{46}NaO_4Si$ $[M+Na]^+$: 533.3058, found: 533.3054.

$[\alpha]_D^{25}$ = +16.7 (c = 0.395, $CHCl_3$)

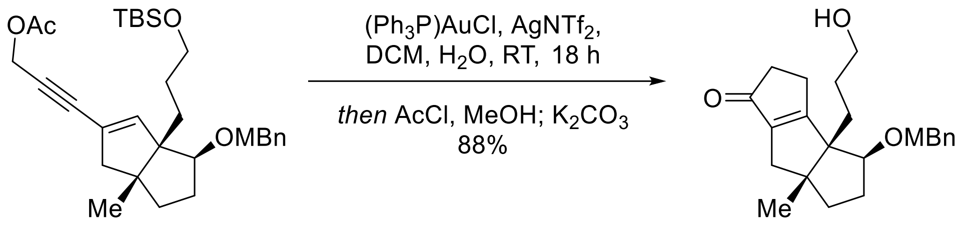

10 **11**

A round-bottom flask covered by aluminium foil was charged with $(Ph_3P)AuCl$ (3.68 g, 7.44 mmol, 0.10 eq.), $AgNTf_2$ (3.46 g, 8.93 mmol, 0.12 eq.) and DCM (250 mL), and the resulting white suspension was stirred for 30 min. Then, enyne **10** (38.0 g, 74.4 mmol, 1.00 eq.) dissolved in DCM (125 mL) was added, followed by H_2O (0.9 mL). After 18 h the mixture was filtered through a pad of Celite and concentrated. The dark residue was redissolved in MeOH (220 mL), cooled down to 0 °C and AcCl (0.79 mL, 11.2 mmol, 0.15 eq.) was added. After 1 h, finely powdered K_2CO_3 (30.8 g, 223 mmol, 3.00 eq.) was added and the mixture was allowed to warm up to RT. After 1 h, sat. aq. NH_4Cl soln. (350 mL) and DCM (500 mL) were added, and the layers were separated. The aqueous phase was extracted with DCM (3 × 400 mL), the combined organic phase was washed once with brine (500 mL), dried over anhydrous Na_2SO_4, and concentrated. The residue was purified by flash column chromatography (*n*-hexane/EtOAc 1:4) to give pure **11** (23.2 g, 63.8 mmol, 88%).

Appearance: pale-yellow syrup.

R_f (SiO_2, *n*-hexane/EtOAc 1:4, $KMnO_4$): 0.45.

FTIR (thin film, neat, cm^{-1}): 3410, 2944, 2865, 1687, 1633, 1516, 1441, 1381, 1213, 1140, 1065, 803, 482.

^{1}H-NMR (400 MHz, $CDCl_3$) δ 7.21 (d, *J* = 8.0 Hz, 2H), 7.16 (d, *J* = 7.9 Hz, 2H), 4.56 (d, *J* = 11.7 Hz, 1H), 4.32 (d, *J* = 11.7 Hz, 1H), 3.89 (dd, *J* = 4.0, 1.6 Hz, 1H), 3.60 (t, *J* = 6.5 Hz, 2H), 2.72 – 2.58 (m, 2H), 2.52 – 2.38 (m, 2H), 2.35 (s, 4H), 2.23 (dt, *J* = 16.1, 3.1 Hz, 1H), 1.94 – 1.80 (m, 3H), 1.71 – 1.61 (m, 2H), 1.55 (ttd, *J* = 12.8, 6.5, 4.4 Hz, 1H), 1.40 (dddd, *J* = 15.5, 12.9, 11.4, 7.6 Hz, 3H), 1.23 (s, 3H).

^{13}C{^{1}H} NMR (101 MHz, $CDCl_3$) δ 204.5, 189.0, 147.6, 137.3, 135.8, 129.1, 127.5, 83.4, 70.9, 64.1, 63.6, 56.0, 41.8, 41.0, 40.0, 29.4, 29.1, 25.9, 25.4, 24.4, 21.3.

HRMS (ESI, *m/z*): calc. for $C_{23}H_{31}O_3$ $[M+H]^+$: 355.2268, found: 355.2268.

$[\alpha]_D^{25}$ = +46.2 (c = 0.435, $CHCl_3$).

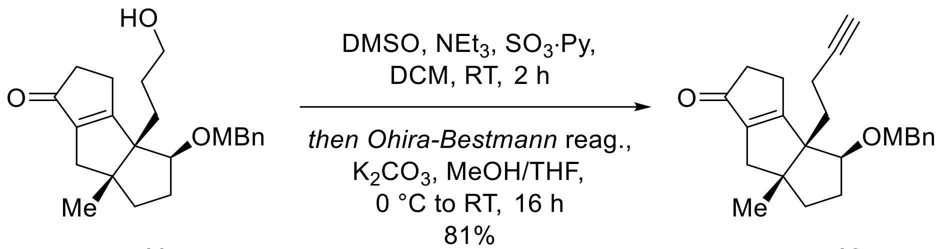

11 **12**

To a solution of alcohol **11** (22.9 g, 65.1 mmol, 1.00 eq.) in dry DCM (220 mL), DMSO (46.2 mL, 651 mmol, 10.0 eq.) and NEt_3 (45.8 mL, 326 mmol, 5.00 eq.) were added. The solution was cooled down to 0 °C and SO_3·Py (41.5 g, 260 mmol, 4.00 eq.) was added. The solution was allowed to warm up to RT and after 2 h, it was poured into sat. aq. NH_4Cl soln. (400 mL). The layers were separated and the aqueous phase was extracted with DCM (3 × 150 mL). The combined organic layers were washed once with 1 M HCl (400 mL), sat. aq. $NaHCO_3$ soln. (500 mL) and brine (500 mL). The combined organic phase was dried over anhydrous Na_2SO_4 and concentrated under N_2. The residue, dissolved in dry THF (40 mL), was added to a suspension finely powdered K_2CO_3 (18.0 g, 130 mmol, 2.00 eq.) in MeOH (220 mL) and the suspension was cooled down to 0 °C. *Ohira-Bestmann* reagent (18.8 g, 97.6 mmol, 1.50 eq.) was added, the solution was warmed up to RT and stirred overnight (16 h). Then, sat. aq. NH_4Cl soln. (400 mL) and Et_2O (300 mL) were added and the layers were separated. The aqueous phase was extracted with Et_2O (3 × 150 mL), the combined organic phase was washed with brine (400 mL), dried over anhydrous Na_2SO_4 and concentrated. The residue was purified by flash column chromatography (*n*-hexane/EtOAc 2:1) to give pure **12** (18.3 g, 52.5 mmol, 81%).

Appearance: viscous pale-yellow oil.

R_f (*n*-hexane/EtOAc 2:1, $KMnO_4$): 0.41.

FTIR (thin film, neat, cm^{-1}): 3290, 2934, 2865, 1693, 1638, 1516, 1442, 1379, 1350, 1262, 1220, 1138, 1073, 1020, 802, 633, 570, 481.

^{1}H-NMR (400 MHz, $CDCl_3$) δ 7.23 (d, J = 8.0 Hz, 2H), 7.17 (d, J = 7.9 Hz, 2H), 4.56 (d, J = 11.7 Hz, 1H), 4.34 (d, J = 11.7 Hz, 1H), 3.86 (dd, J = 4.0, 1.6 Hz, 1H), 2.73 – 2.58 (m, 2H), 2.55 – 2.43 (m, 1H), 2.41 – 2.31 (m, 4H), 2.27 – 1.99 (m, 4H), 1.99 – 1.95 (m, 1H), 1.95 – 1.80 (m, 2H), 1.70 – 1.59 (m, 1H), 1.46 – 1.31 (m, 1H), 1.24 (s, 3H).

^{13}C{^{1}H} NMR (101 MHz, $CDCl_3$) δ 204.2, 187.8, 148.2, 137.3, 135.6, 129.1, 127.5, 84.5, 83.1, 70.8, 68.6, 64.0, 56.1, 41.8, 41.0, 40.0, 29.3, 29.3, 25.4, 24.4, 21.3, 15.3.

HRMS (ESI, *m/z*): calc. for $C_{24}H_{29}O_2$ $[M+H]^+$: 349.2162, found: 349.2158.

$[\alpha]_D^{25}$ = +23.0 (c = 0.46, $CHCl_3$).

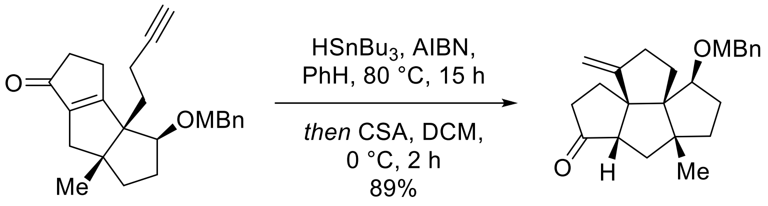

12 **13**

Alkyne **12** (18.3 g, 52.5 mmol, 1.00 eq.) was dissolved in dry benzene (900 mL) and $HSnBu_3$ (28.3 mL, 30.6 g, 31.4 mmol, 2.00 eq.) and AIBN (0.86 g, 5.25 mmol, 0.10 eq.) were added at RT. The solution was heated to 80 °C and stirred overnight (15 h). The solution was cooled down to RT and concentrated *in vacuo*. The crude was passed through a pad of silica (*n*-hexane → *n*-hexane/EtOAc 10:1). The residue (29.8 g, 46.7 mmol, 1.00 eq.) was redissolved in dry DCM (350 mL) and cooled down to 0 °C. Then CSA (17.1 g, 73.5 mmol, 1.4 eq.) was added in one portion and the resulting yellow solution was allowed to stir for 1 h. The solution was quenched by addition of sat. aq. $NaHCO_3$ soln. (100 mL) and H_2O (100 mL) and then extracted with DCM (3 × 50 mL). The combined organic fractions were dried over anhydrous Na_2SO_4 and concentrated. The residue was purified by flash column chromatography (*n*-hexane/EtOAc 15:1) to give pure **13** (16.4 g, 46.9 mmol, 89%).

Appearance: colorless oil.

R_f (SiO_2, *n*-hexane/EtOAc 15:1, $KMnO_4$): 0.22

FTIR (thin film, neat, cm^{-1}): 2949, 2869, 1735, 1648, 1516, 1458, 1412, 1376, 1353, 1175, 1079, 1021, 879, 802, 480.

^{1}H-NMR (400 MHz, $CDCl_3$) δ 7.21 (d, *J* = 8.1 Hz, 2H), 7.14 (d, *J* = 7.7 Hz, 2H), 4.84 (t, *J* = 1.9 Hz, 1H), 4.78 (t, *J* = 2.0 Hz, 1H), 4.55 (d, *J* = 11.6 Hz, 1H), 4.32 (d, *J* = 11.6 Hz, 1H), 3.69 (t, *J* = 6.1 Hz, 1H), 2.67 – 2.48 (m, 2H), 2.48 – 2.26 (m, 6H), 2.21 – 2.06 (m, 2H), 2.06 – 1.82 (m, 3H), 1.81 – 1.67 (m, 2H), 1.56 – 1.49 (m, 2H), 1.37 – 1.24 (m, 1H), 1.04 (s, 3H).

^{13}C{^{1}H} NMR (101 MHz, $CDCl_3$) δ 221.9, 163.1, 137.2, 135.9, 129.1, 127.6, 103.2, 82.6, 71.3, 70.1, 64.7, 61.2, 53.9, 42.8, 39.2, 36.8, 34.0, 30.9, 30.0, 26.7, 24.4, 21.3.

HRMS (ESI, *m/z*): calc. for $C_{24}H_{30}NaO_2$ $[M+Na]^+$: 373.2138, found: 373.2140.

$[\alpha]_D^{25}$ = +120.9 (c = 0.335, $CHCl_3$).

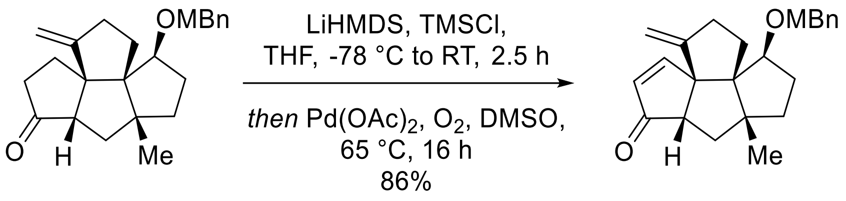

13 **14**

To a solution of ketone **13** (10.3 g, 29.5 mmol, 1.00 eq.) in dry THF (120 mL) at –78 °C LiHMDS (1 M in *n*-hexane, 35.4 mL, 35.4 mmol, 1.20 eq.) was added dropwise. After 1 h at –78 °C, TMSCl (5.28 mL, 4.49 g, 41.3 mmol, 1.40 eq.) was added to the yellow solution, which was allowed to warm up to RT. After 1.5 h at RT, the solution was cooled down to 0 °C and carefully poured into cold 1M PBS (250 mL). The layers were separated with a pre-cooled separatory funnel (–20 °C) and the aqueous one was extracted with DCM (3 × 50 mL). The combined organic phase was dried over anhydrous Na_2SO_4 and concentrated (30 °C) under reduced N_2 atmosphere. The residue was redissolved in dry DMSO (200 mL) and the atmosphere was exchanged with O_2. Then, $Pd(OAc)_2$ (0.66 g, 2.95 mmol, 0.10 eq.) was added, and the solution was left stirring at 65 °C overnight (16 h) under O_2 atmosphere. The resulting black solution was cooled down to RT and diluted with cold H_2O (1 L) and extracted with DCM (5 × 150 mL) dried over anhydrous Na_2SO_4 and concentrated. The residue was purified by flash column chromatography (*n*-hexane/EtOAc 1:15 → 1:10) to give pure **14** (8.84 g, 25.4 mmol, 86%).

Appearance: pale-yellow oil.

R_f (SiO_2, *n*-hexane/EtOAc 10:1, $KMnO_4$): 0.40.

FTIR (thin film, neat, cm^{-1}): 2938, 2867, 1704, 1648, 1579, 1516, 1456, 1357, 1201, 1067, 1019, 886, 802, 576, 480.

^{1}H-NMR (400 MHz, $CDCl_3$) δ 7.36 (d, *J* = 5.5 Hz, 1H), 7.20 (d, *J* = 8.0 Hz, 2H), 7.15 (d, *J* = 8.0 Hz, 2H), 6.01 (d, *J* = 5.5 Hz, 1H), 4.94 (s, 1H), 4.62 (t, *J* = 2.1 Hz, 1H), 4.55 (d, *J* = 11.7 Hz, 1H), 4.28 (d, *J* = 11.7 Hz, 1H), 3.81 (dd, *J* = 6.5, 4.6 Hz, 1H), 2.59 (dd, *J* = 10.2, 4.9 Hz, 1H), 2.54 – 2.41 (m, 2H), 2.36 – 2.28 (m, 4H), 1.99–1.78 (m, 3H), 1.64 – 1.43 (m, 2H), 1.38 – 1.24 (m, 2H), 1.07 (s, 3H).

$^{13}C\{^1H\}$ NMR (101 MHz, $CDCl_3$) δ 214.1, 167.8, 157.6, 137.2, 135.9, 131.3, 129.1, 127.5, 107.4, 81.7, 71.4, 71.1, 70.6, 59.8, 54.9, 40.6, 36.7, 34.0, 30.1, 26.5, 24.6, 21.3.

HRMS (ESI, *m/z*): calc. for $C_{24}H_{29}O_2$ $[M+H]^+$: 349.2162, found: 349.2164.

$[\alpha]_D^{25}$ = +119.0 (c = 0.703, $CHCl_3$).

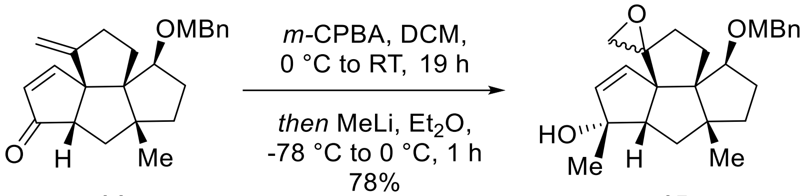

14 **15**

To a solution of enone **14** (8.60 g, 24.7 mmol, 1.00 eq.) in DCM (120 mL) *m*-CPBA (7.91 g, 32.1 mmol, 70–75%, 1.30 eq.) was added at 0 °C and the solution was allowed to warm up to RT overnight (19 h). The mixture was quenched by addition of sat. aq. $Na_2S_2O_3$ soln. (20 mL) and sat. aq. $NaHCO_3$ soln. (120 mL). The layers were separated and the aqueous phase was extracted with DCM (3 × 100 mL). The combined organic phases were dried over anhydrous Na_2SO_4 and concentrated. The residue was redissolved in dry Et_2O (120 mL) and cooled down to –78 °C. Then, MeLi (1.6 M in Et_2O, 20.1 mL, 1.30 eq.) was added dropwise. The solution was allowed to stir for 5 min, after which the solution was allowed to warm up to 0 °C over 20 min. After 1 h at 0 °C, the reaction was quenched by addition of sat. aq. NH_4Cl soln. (100 mL). The layers were separated and the aqueous phase was extracted with DCM (3 × 50 mL). The combined organic phase was dried over anhydrous Na_2SO_4 and concentrated. The residue was purified by flash column chromatography (*n*-hexane/EtOAc 4:1 to 2:1) to give **15** (7.37 g, 19.4 mmol, 78%) as a mixture of two diastereomers (3.7:1).

Appearance: colorless oil

R_f (SiO_2, *n*-hexane/EtOAc 3:1, $KMnO_4$): 0.51, 0.43

FTIR (thin film, neat, cm^{-1}): 3459, 2951, 2868, 1517, 1452, 1367, 1130, 1102, 1067, 1021, 934, 875, 800, 764, 574, 485.

^{1}H-NMR (400 MHz, $CDCl_3$) (major diastereomer) δ 7.23 (d, *J* = 7.8 Hz, 2H), 7.14 (d, *J* = 7.7 Hz, 2H), 5.61 (d, *J* = 5.6 Hz, 1H), 5.56 (d, *J* = 5.6 Hz, 1H), 4.52 (d, *J* = 11.6 Hz, 1H), 4.38 (d, *J* = 11.7 Hz, 1H), 3.87 (t, *J* = 5.9 Hz, 1H), 2.76 (d, *J* = 4.5 Hz, 1H), 2.64 (d, *J* = 4.5 Hz, 1H), 2.34 (s, 3H), 2.27 (ddd, *J* = 12.9, 10.6, 7.4 Hz, 1H), 2.04 (ddd, *J* = 13.0, 10.4, 6.8 Hz, 2H), 1.97 – 1.80 (m, 2H), 1.80 – 1.58 (m, 6H), 1.58 – 1.47 (m, 2H), 1.43 (d, *J* = 3.3 Hz, 1H), 1.35 (s, 3H), 1.11 (s, 3H).

^{13}C{^{1}H} NMR (101 MHz, $CDCl_3$) (major diastereomer) δ 138.7, 136.9, 136.4, 132.8, 129.0, 127.4, 84.2, 82.6, 72.0, 71.2, 70.0, 69.8, 58.4, 53.3, 48.5, 43.4, 37.0, 33.3, 30.2, 30.0, 25.3, 25.0, 21.3.

HRMS (ESI, *m/z*): calc. for $C_{25}H_{32}KO_3$ $[M+K]^+$: 419.1983, found: 419.1981.

$[\alpha]_D^{25}$ = +56.9 (c = 0.670, $CHCl_3$).

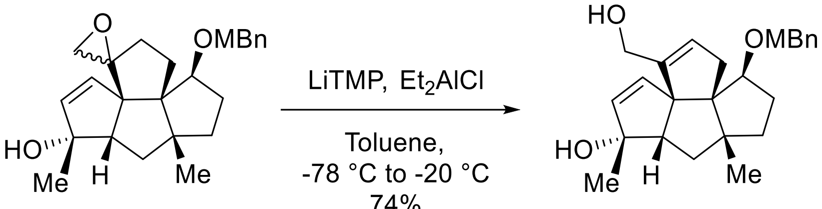

15 **16**

A solution of freshly distilled 2,2,6,6-tetramethylpiperidine (13.5 mL, 79.1 mmol, 4.1 eq.) in toluene (90 mL) was cooled down to 0 °C and *n*-BuLi (31.3 mL, 78.2 mmol, 2.5 M in *n*-hexane, 4.05 eq.) was added dropwise resulting in a yellow suspension. After 40 min, diethylaluminium chloride (77.2 mL, 77.2 mmol, 1 M in *n*-hexane, 4.00 eq.) was added over 10 min, resulting in a white suspension. After 70 min, the suspension was cooled down to –78 °C and epoxide **15** (7.35 g, 19.3 mmol, 1.00 eq.), dissolved in toluene (25 mL), was added dropwise over 10 min. Residual dry ice in the dry-ice bath was removed and the solution was allowed to slowly warm up to –20 °C (4–6 h). After stirring the solution at –20 °C for 30 min, the typically bright yellow solution was quenched by addition of MeOH (15 mL), followed by addition of sat. aq. *Rochelle* salt soln. (250 mL). The mixture was stirred for 1 h and the layers were separated. The aqueous phase was extracted with EtOAc (3 × 100mL), the combined organic phase was dried over anhydrous Na_2SO_4, filtrated and concentrated. The residue was purified by flash column chromatography (*n*-hexane/EtOAc 1:1) to give pure **16** (5.46 g, 14.3 mmol, 74%).

Appearance: white foam.

R_f (SiO_2, *n*-hexane/EtOAc 1:2, $KMnO_4$): 0.09.

FTIR (thin film, neat, cm^{-1}): 3362, 3048, 2951, 2867, 1690, 1517, 1455, 1367, 1203, 1098, 1067, 1048, 1022, 874, 803, 601, 580, 481, 427, 407.

^{1}H-NMR (400 MHz, Chloroform-*d*) δ 7.20 (d, *J* = 8.1 Hz, 2H), 7.13 (d, *J* = 7.7 Hz, 2H), 5.64 (d, *J* = 5.5 Hz, 1H), 5.61 (s, 1H), 5.50 (d, *J* = 5.5 Hz, 1H), 4.47 (d, *J* = 11.4 Hz, 1H), 4.34 (d, *J* = 11.4, 1H), 4.14 – 4.00 (m, 2H), 3.90 (dd, *J* = 9.4, 7.1 Hz, 1H), 2.80 (dq, *J* = 17.4, 2.4 Hz, 1H), 2.55 (dd, *J* = 11.3, 8.2 Hz, 1H), 2.34 (s, 3H), 2.23 – 2.13 (m, 1H), 2.12 – 2.03 (m, 1H), 1.79 – 1.68 (m, 1H), 1.67 – 1.49 (m, 5H), 1.47 (s, 3H), 1.43 (br s, 1H), 1.32 – 1.24 (m, 1H), 0.96 (s, 3H).

^{13}C{^{1}H} NMR (101 MHz, $CDCl_3$) δ 145.8, 137.1, 137.0, 136.2, 136.1, 129.0, 127.7, 126.4, 82.3, 77.0, 71.4, 68.1, 60.6, 56.2, 53.4, 42.7, 34.1, 30.5, 30.2, 30.0, 23.2, 21.3.

HRMS (ESI, *m/z*): calc. for $C_{25}H_{32}O_3Na$ $[M+Na]^+$: 403.2249, found: 403.2252.

$[\alpha]_D^{25}$ = +86.2 (c = 0.783, $CHCl_3$).

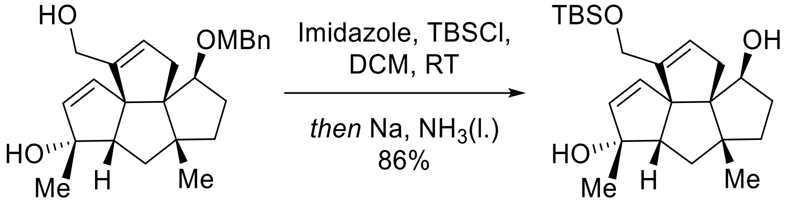

16 **17**

To a solution of diol **16** (3.85 g, 10.1 mmol, 1.00 eq.) in DCM (100 mL) imidazole (1.72 g, 25.3 mmol, 2.50 eq.) and TBSCl (1.60 g, 10.6 mmol, 1.05 eq.) were added and the solution was stirred at RT for 3 h. Then, sat. aq. NH_4Cl (150 mL) was added, the layers were separated and the aqueous phase was extracted with DCM (3 × 80 mL). The combined organic phase was dried over anhydrous Na_2SO_4, and concentrated. The residue was dissolved in THF (20 mL) and added to a solution of Na (2.32 g, 101 mmol, 10.0 eq.) in liquid NH_3 (100 mL) at –42 °C. After 6 h of vigorous stirring at -42 °C the reaction was quenched by addition of solid NH_4Cl and allowed to warm up to RT. Then, DCM (150 mL) and H_2O (150 mL) were added and the layers were separated. The aqueous phase was extracted with DCM (3 × 50 mL) and the combined organic phase was dried over anhydrous Na_2SO_4, filtered and concentrated. The residue was purified by flash column chromatography (*n*-hexane/EtOAc 1:1) to give pure **17** (3.41 g, 8.73 mmol, 86%).

Appearance: white solid.

MP: 101-102.

R_f (SiO_2, *n*-hexane/EtOAc 1:1, $KMnO_4$): 0.46.

FTIR (thin film, neat, cm^{-1}): 3381, 2928, 2857, 1463, 1256, 1085, 838, 775.

^{1}H-NMR (400 MHz, Chloroform-*d*): δ 5.61 – 5.55 (m, 3H), 4.26 (t, *J* = 8.8 Hz, 1H), 4.12 – 4.04 (m, 1H), 3.96 (ddt, *J* = 14.2, 3.1, 1.6 Hz, 1H), 2.62 – 2.53 (m, 2H), 2.11 – 2.02 (m, 2H), 1.79 – 1.68 (m, 1H), 1.68 – 1.52 (m, 3H), 1.49 – 1.40 (m, 5H), 1.36 – 1.27 (m, 1H), 0.98 (s, 3H), 0.89 (s, 9H), 0.04 (s, 6H).

^{13}C{^{1}H} NMR (101 MHz, CDCl3): δ 145.8, 137.4, 135.1, 124.7, 82.3, 76.7, 75.5, 68.2, 60.7, 55.7, 54.0, 42.9, 33.9, 33.7, 30.5, 29.1, 26.0, 23.5, 18.5, -5.2, -5.2.

HRMS (ESI, *m/z*): calc. for $C_{23}H_{38}O_3SiNa$ $[M+Na]^+$: 413.2482, found: 413.2483.

$[\alpha]_D^{25}$ = –34.4 (c = 0.613, $CHCl_3$).

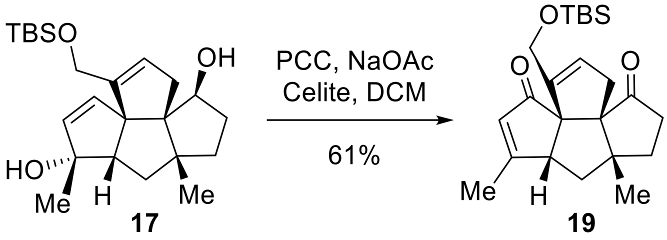

Diol **17** (1.58 g, 4.04 mmol, 1.00 eq.) in of dry DCM (10 mL) was slowly added to a suspension of PCC (10.5 g, 48.5 mmol, 12.0 eq.), NaOAc (4.69 g, 56.6 mmol, 14.0 eq.) and Celite (15 g) in dry DCM (240 ml) at RT. The resulting darkened suspension was allowed to stir overnight (15 h). The reaction was diluted with 300 mL of Et_2O, and the resulting suspension was filtered through a pad of silica (Et_2O), the filtrate was concentrated and the residue was purified by flash column chromatography (*n*-hexane/EtOAc 2:1) to give pure **19** (960 mg, 2.48 mmol, 61%).

Appearance: white solid.

MP: 103-105.

R_f (SiO_2, *n*-hexane/EtOAc 2:1, $KMnO_4$, UV): 0.43.

FTIR (thin film, neat, cm^{-1}): 2953, 2927, 2893, 2856, 1737, 1695, 1618, 1471, 1462, 1434, 1410, 1378, 1361, 1330, 1303, 1283, 1254, 1230, 1216, 1186, 1152, 1085, 1065, 1039, 833, 775.

^{1}H-NMR (400 MHz, $CDCl_3$): δ 5.87 (t, *J* = 1.6 Hz, 1H), 5.78 (t, *J* = 1.2 Hz, 1H), 4.06 (dd, *J* = 12.9, 3.8 Hz, 1H), 3.94–3.84 (m, 1H), 3.40 (dddd, *J* = 10.0, 3.1, 1.9, 1.0 Hz, 1H), 2.60 (dtd, *J* = 17.1, 2.5, 1.4 Hz, 1H), 2.40–2.32 (m, 1H), 2.32–2.24 (m, 1H), 2.23–2.13 (m, 1H), 2.12 (s, 3H), 2.05–1.90 (m, 2H), 1.77 (ddd, *J* = 13.7, 10.2, 8.7 Hz, 1H), 1.66–1.55 (m, 1H), 0.99 (s, 3H), 0.82 (s, 9H), –0.03 (s, 3H), -0.06 (s, 3H).

^{13}C{^{1}H} NMR (101 MHz, $CDCl_3$): δ 218.1, 208.0, 180.5, 142.0, 130.3, 129.0, 79.3, 74.5, 60.5, 57.4, 53.0, 39.7, 35.3, 35.0, 32.0, 26.0, 23.9, 18.3, 18.1, –5.5, –5.56.

HRMS (ESI, *m/z*): calc. for $C_{23}H_{35}O_3Si$ $[M+H]^+$: 387.23500, found: 387.23558.

$[\alpha]_D^{25}$ = –90.6 (c = 0.580, $CHCl_3$).

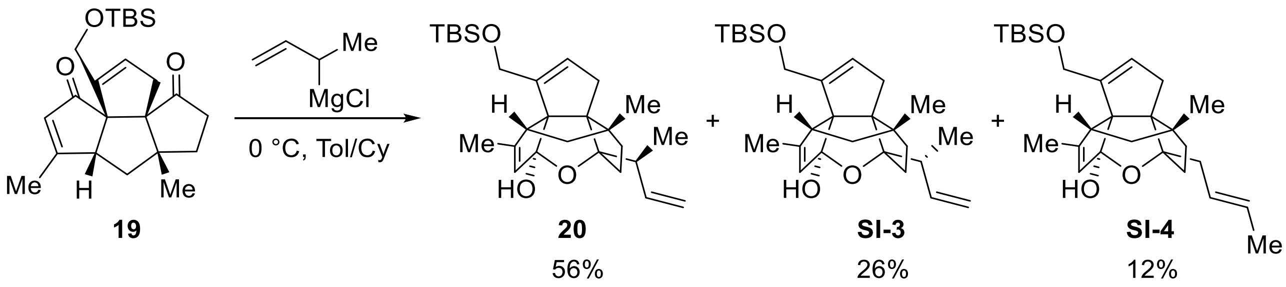

1-Methyl-2-propenylmagnesium chloride (0.5 M in THF, 12.4 mL, 6.21 mmol, 3.0 eq.) was transferred to a round-bottom flask; THF was removed *in vacuo* and the resulting gel was redissolved in dry toluene (15 mL). This solution then was added to the solution of **19** (800 mg, 2.07 mg, 1.00 eq.) in dry cyclohexane (40 mL) and dry toluene (5 mL) dropwise at 0 °C. After stirring the reaction for 20 min, it was quenched with sat. aq. NH_4Cl soln. (50 mL). The layers were separated and the aqueous phase was extracted with DCM (3 × 20 mL). The combined organic phase was dried over anhydrous Na_2SO_4 and concentrated. The residue was first submitted to flash column chromatography (*n*-hexane/EtOAc 50:1 → 15:1) to obtain mixture of **20** and **SI-3** together with **SI-4** (105 mg, 0.237 mmol, 12%). Then the former mixture was separated by flash column chromatography (*n*-hexane/acetone 90:1 → 60:1) to obtain **20** (512 mg, 1.16 mmol, 56%) and **SI-3** (235 mg, 0.531 mmol, 26%).

Characterization data for **20**:

Appearance: colorless oil.

R_f (SiO_2, *n*-hexane/EtOAc 20:1, $KMnO_4$): 0.19.

R_f (SiO_2, *n*-hexane/acetone 20:1, $KMnO_4$): 0.24.

FTIR (thin film, neat, cm^{-1}): 3362, 2955, 2928, 2880, 2856, 1462, 1255, 1111, 1082, 1073, 1014, 998, 899, 837, 776.

^{1}H NMR (400 MHz, $CDCl_3$) δ 6.30 – 6.13 (m, 1H), 5.59 – 5.51 (m, 1H), 5.41 – 5.33 (m, 1H), 5.11 – 5.07 (m, 1H), 5.08 – 5.03 (m, 1H), 4.45 (dtd, *J* = 14.4, 2.8, 1.8 Hz, 1H), 4.14 (ddt, *J* = 14.4, 2.8, 1.4 Hz, 1H), 3.65 (s, 1H), 2.86 (ddt, *J* = 9.9, 2.2, 1.2 Hz, 1H), 2.65 – 2.55 (m, 1H), 2.49 – 2.41 (m, 1H), 2.41 – 2.33 (m, 1H), 1.91 – 1.77 (m, 3H), 1.73 (t, *J* = 1.3 Hz, 3H), 1.61 – 1.46 (m, 2H), 1.33 – 1.24 (m, 1H), 1.12 (d, *J* = 6.9 Hz, 3H), 0.98 (s, 3H), 0.91 (s, 10H), 0.07 (s, 3H), 0.07 (s, 3H).

^{13}C{^{1}H} NMR (101 MHz, $CDCl_3$) δ 145.2, 142.5, 142.2, 128.6, 128.0, 118.3, 114.9, 100.8, 86.8, 77.6, 61.2, 56.8, 56.7, 43.6, 40.0, 38.6, 37.0, 34.3, 26.1, 25.1, 18.5, 16.5, 15.2, -5.3, -5.3.

HRMS (ESI, *m/z*): calc. for $C_{27}H_{42}O_3SiNa$ $[M+Na]^+$: 465.2795, found: 465.2790.

$[\alpha]_D^{25}$ = –39.3 (c = 0.743, $CHCl_3$).

Characterization data for **SI-3**:

Appearance: colorless oil.

R_f (SiO_2, *n*-hexane/EtOAc 20:1, $KMnO_4$): 0.19.

R_f (SiO_2, *n*-hexane/acetone 20:1, $KMnO_4$): 0.21.

FTIR (thin film, neat, cm^{-1}): 3412, 2954, 2928, 2882, 2856, 1255, 1109, 1066, 1011, 1002, 899, 837, 776.

^1H NMR (400 MHz, $CDCl_3$) δ 5.88 (ddd, *J* = 17.1, 10.2, 8.8 Hz, 1H), 5.65 – 5.58 (m, 1H), 5.43 – 5.38 (m, 1H), 4.96 (ddd, *J* = 17.2, 2.1, 0.8 Hz, 1H), 4.92 (dd, *J* = 10.1, 2.1 Hz, 1H), 4.42 – 4.34 (m, 1H), 4.27 – 4.21 (m, 1H), 4.05 (s, 1H), 2.86 (d, *J* = 9.7 Hz, 1H), 2.60 – 2.52 (m, 1H), 2.39 – 2.26 (m, 2H), 1.91 – 1.82 (m, 2H), 1.74 (t, *J* = 1.3 Hz, 3H), 1.65 (ddd, *J* = 13.4, 5.8, 1.0 Hz, 1H), 1.55 – 1.45 (m, 2H), 1.26 (ddd, *J* = 12.2, 6.4, 1.0 Hz, 1H), 1.06 (d, *J* = 6.6 Hz, 3H), 0.96 (s, 3H), 0.89 (s, 9H), 0.06 (s, 3H), 0.06 (s, 3H).

$^{13}C\{^1H\}$ NMR (101 MHz, $CDCl_3$) δ 144.5, 142.7, 142.0, 129.2, 128.9, 118.3, 113.7, 100.9, 87.3, 61.2, 56.8, 56.6, 45.8, 40.1, 39.4, 37.1, 34.3, 26.0, 24.9, 18.5, 16.9, 15.2, -5.4, -5.4.

HRMS (ESI, *m/z*): calc. for $C_{27}H_{42}O_3SiNa$ $[M+Na]^+$: 465.2795, found: 465.2791.

$[\alpha]_D^{25}$ = –27.4 (c = 1.10, $CHCl_3$).

Characterization data for **SI-4**:

Appearance: colorless oil.

R_f (SiO_2, *n*-hexane/EtOAc 20:1, $KMnO_4$): 0.17.

FTIR (thin film, neat, cm^{-1}): 3352, 2952, 2927, 1461, 1444, 1376, 1254, 1111, 1081, 1064, 1023, 1002, 835, 775.

^1H NMR (400 MHz, $CDCl_3$) δ 5.66 – 5.50 (m, 3H), 5.38 (t, *J* = 1.7 Hz, 1H), 4.47 – 4.36 (m, 1H), 4.25 – 4.14 (m, 1H), 3.78 (s, 1H), 2.94 – 2.82 (m, 1H), 2.55 – 2.42 (m, 2H), 2.42 – 2.24 (m, 2H), 1.91 – 1.84 (m, 2H), 1.82 – 1.76 (m, 1H), 1.73 (s, 3H), 1.62 (d, *J* = 5.0 Hz, 3H), 1.60 – 1.46 (m, 2H), 1.35 – 1.27 (m, 1H), 0.98 (s, 3H), 0.90 (s, 9H), 0.06 (s, 6H).

$^{13}C\{^1H\}$ NMR (101 MHz, $CDCl_3$) δ 145.0, 142.4, 128.6, 127.8, 127.5, 126.1, 118.6, 99.3, 87.5, 76.9, 61.3, 56.5, 56.4, 40.9, 39.2, 37.7, 35.9, 33.9, 26.0, 25.2, 18.5, 15.3, 13.2, -5.4, -5.4.

HRMS (ESI, *m/z*): calc. for $C_{27}H_{42}O_3SiNa$ $[M+Na]^+$: 465.2795, found: 465.2794.

$[\alpha]_D^{25}$ = –31.2 (c = 0.533, $CHCl_3$).

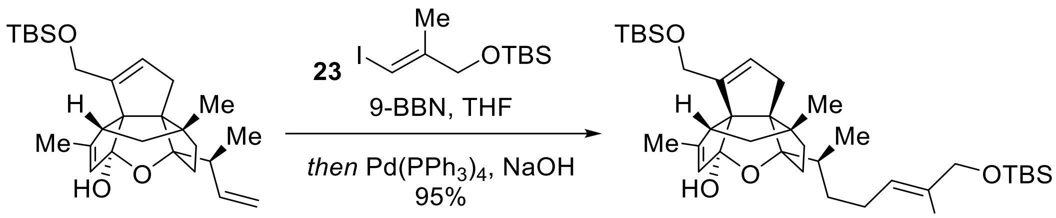

To the solution of hemiacetal **20** (400 mg, 0.904 mmol, 1.00 eq.) in dry THF (9 ml) 9-BBN (0.5 M in THF, 9.0 mL, 4.5 mmol, 4.98 eq) was added. The resulting solution was heated to 60 °C and stirred for 1 h. Then it was cooled down to 0 °C and NaOH (2 M in water, 6.8 mL, 13.6 mmol, 15.0 eq.) was added to it. The reaction mixture was vigorously stirred for 20 min at 0 °C before vinyl iodide **23** (564 mg, 1.81 mmol, 2.00 eq.)[2] and $Pd(PPh_3)_4$ (60.3 mg, 0.0904 mmol, 10 mol%) were added, and the reaction was heated at 65 °C. After 20 min TLC showed full conversion and the reaction was cooled down to RT and quenched with sat. aq. NH_4Cl soln. (40 mL). The layers were separated, and the aqueous layer was extracted with DCM (3 × 15 mL). The combined organic phase was dried over anhydrous Na_2SO_4 and concentrated. The residue was purified by flash column chromatography (*n*-hexane/EtOAc 30:1) to give **24** (542 mg, 0.862 mmol, 95 %).

Appearance: colorless oil.

R_f (SiO_2, *n*-hexane/EtOAc 20:1, $KMnO_4$): 0.22.

FTIR (thin film, neat, cm^{-1}): 3412, 2928, 2856, 1463, 1376, 1253, 1073, 1006, 836, 775.

^1H NMR (500 MHz, $CDCl_3$) δ 5.60 (s, 1H), 5.43 – 5.34 (m, 2H), 4.46 – 4.38 (m, 1H), 4.19 (d, *J* = 14.0 Hz, 1H), 4.00 (s, 2H), 3.50 (s, 1H), 2.87 (d, *J* = 9.6 Hz, 1H), 2.44 (d, *J* = 18.1 Hz, 1H), 2.39 – 2.29 (m, 1H), 2.19 – 2.09 (m, 1H), 2.06 – 1.95 (m, 1H), 1.92 – 1.81 (m, 2H), 1.77 (dd, *J* = 13.0, 5.8 Hz, 1H), 1.74 (s, 3H), 1.70 – 1.61 (m, 1H), 1.60 (s, 3H), 1.56 – 1.45 (m, 3H), 1.28 – 1.24 (m, 1H), 1.13 (dtd, *J* = 13.5, 8.9, 4.8 Hz, 1H), 0.98 (d, *J* = 6.6 Hz, 3H), 0.95 (s, 3H), 0.91 (s, 9H), 0.90 (s, 9H), 0.06 (s, 6H), 0.06 (s, 6H).

$^{13}C\{^1H\}$ NMR (126 MHz, $CDCl_3$) δ 144.81, 142.41, 134.38, 129.09, 128.73, 124.98, 118.16, 102.49, 87.03, 77.52, 68.89, 61.13, 56.88, 56.72, 39.98, 39.23, 38.31, 36.99, 34.37, 32.06, 26.13, 26.06, 25.95, 24.86, 18.58, 18.49, 16.04, 15.14, 13.58, -5.08, -5.29, -5.36.

HRMS (ESI, *m/z*): calc. for $C_{37}H_{64}O_4Si_2Na$ $[M+Na]^+$: 651.4253, found: 651.4253.

$[\alpha]_D^{25}$ = –18.0 (c = 0.920, $CHCl_3$).

[2] X.-B. Ding, D. P. Furkert, M. A. Brimble, *Chem. Commun.*, **2016**, *52*, 12638-12641.

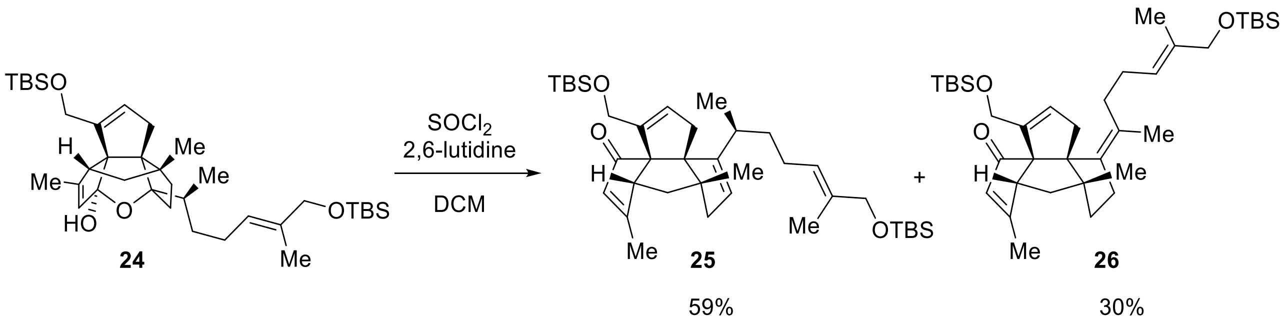

To the solution of **24** (470 mg, 0.747 mmol, 1.00 eq.) in dry DCM (12 mL) and 2,6-lutidine (1.31 mL, 1.20 g, 11.2 mmol, 15.0 eq.) $SOCl_2$ (0.325 mL, 533 mg, 4.48 mmol, 6.00 eq.) was added dropwise at RT. The reaction was stirred for 20 min before it was quenched with sat. aq. $NaHCO_3$ soln. (20 mL). The layers were separated, and the aqueous layer was extracted with DCM (3 × 10 mL). The combined organic phase was dried over anhydrous Na_2SO_4 and concentrated. The residue was purified by flash column chromatography (*n*-hexane/EtOAc 60:1 → 40/1) to give **25** (269 mg, 0.440 mmol, 59 %) and **26** (136 mg, 0.223 mmol, 30%).

Characterization data for **25**:

Appearance: colorless oil.

R_f (SiO_2, *n*-hexane/EtOAc 20:1, $KMnO_4$, UV): 0.31.

FTIR (thin film, neat, cm^{-1}): 2955, 2928, 2856, 1699, 1623, 1472, 1463, 1376, 1361, 1253, 1067, 836, 775.

^1H NMR (400 MHz, $CDCl_3$) δ 5.82 – 5.74 (m, 1H), 5.69 (t, *J* = 1.4 Hz, 1H), 5.42 – 5.32 (m, 1H), 5.18 (t, *J* = 2.4 Hz, 1H), 4.10 – 3.92 (m, 4H), 3.41 (ddt, *J* = 8.7, 7.3, 1.3 Hz, 1H), 2.40 (dq, *J* = 17.6, 2.3 Hz, 1H), 2.34 – 2.22 (m, 1H), 2.24 – 2.15 (m, 1H), 2.10 (dd, *J* = 16.3, 2.4 Hz, 1H), 2.01 (t, *J* = 1.1 Hz, 3H), 2.00 – 1.89 (m, 3H), 1.81 (dd, *J* = 12.6, 8.9 Hz, 1H), 1.66 (dd, *J* = 12.6, 7.2 Hz, 1H), 1.60 (s, 3H), 1.38 (dddd, *J* = 13.0, 11.3, 5.7, 3.6 Hz, 1H), 1.21 – 1.09 (m, 1H), 1.03 (s, 3H), 0.97 (d, *J* = 6.7 Hz, 3H), 0.90 (s, 9H), 0.85 (s, 9H), 0.05 (s, 6H), 0.00 (s, 3H), -0.02 (s, 3H).

$^{13}C\{^1H\}$ NMR (101 MHz, $CDCl_3$) δ 209.2, 178.7, 155.7, 144.0, 133.8, 129.3, 128.4, 125.8, 121.6, 77.3, 74.4, 69.1, 60.7, 56.6, 55.0, 44.1, 41.2, 38.3, 35.9, 31.9, 26.2, 26.1, 26.0, 23.6, 23.1, 18.6, 18.4, 17.9, 13.5, -5.1, -5.4, -5.5.

HRMS (ESI, *m/z*): calc. for $C_{37}H_{63}O_3Si_2$ $[M+H]^+$: 611.4310, found: 611.4321.

$[\alpha]_D^{25}$ = –63.5 (c = 0.533, $CHCl_3$).

Characterization data for **26**:

Appearance: colorless oil.

R_f (SiO_2, *n*-hexane/EtOAc 20:1, $KMnO_4$, UV): 0.26.

FTIR (thin film, neat, cm^{-1}): 2954, 2927, 2856, 1701, 1622, 1471, 1462, 1375, 1252, 1066, 836, 776.

^{1}H NMR (400 MHz, $CDCl_3$) δ 5.73 – 5.69 (m, 1H), 5.67 (t, *J* = 1.6 Hz, 1H), 5.41 – 5.33 (m, 1H), 4.15 – 4.08 (m, 1H), 4.00 (s, 2H), 3.94 (dq, *J* = 13.3, 2.4 Hz, 1H), 3.20 – 3.13 (m, 1H), 2.75 – 2.65 (m, 1H), 2.47 – 2.39 (m, 1H), 2.37 – 2.18 (m, 3H), 2.05 (s, 3H), 2.04 – 1.86 (m, 4H), 1.71 (dd, *J* = 13.7, 10.0 Hz, 1H), 1.61 (s, 3H), 1.58 (s, 3H), 1.57 – 1.49 (m, 1H), 1.31 – 1.26 (m, 1H), 0.98 (s, 3H), 0.91 (s, 9H), 0.84 (s, 9H), 0.06 (s, 6H), 0.00 (s, 3H), -0.04 (s, 3H).

$^{13}C\{^{1}H\}$ NMR (101 MHz, $CDCl_3$) δ 208.5, 179.2, 143.6, 138.7, 133.7, 132.5, 130.7, 128.5, 125.9, 78.4, 72.4, 69.1, 61.2, 58.1, 57.0, 40.5, 36.8, 36.4, 35.4, 30.6, 27.0, 26.2, 26.0, 24.8, 20.5, 18.6, 18.4, 17.5, 13.5, -5.1, -5.5, -5.5.

HRMS (ESI, *m/z*): calc. for $C_{37}H_{62}O_3Si_2Na$ $[M+Na]^+$: 633.4130, found: 633.4139.

$[\alpha]_D^{25}$ = –+60.1 (c = 0.533, $CHCl_3$).

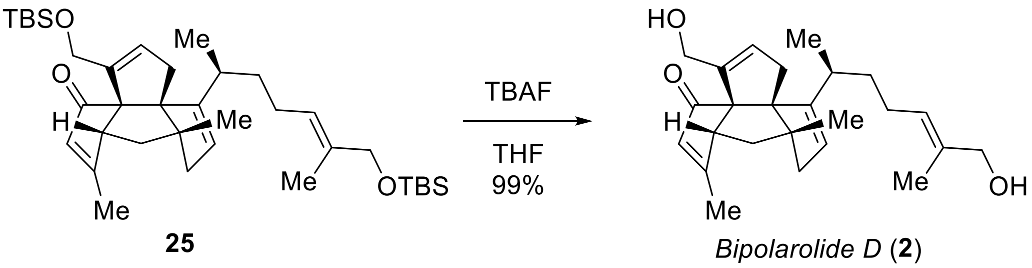

To the solution of **25** (167 mg, 0.273 mmol, 1.00 eq.) in dry THF (2.5 mL) TBAF (1 M in THF, 1.37 mL, 1.37 mmol, 5.00 eq.) was added at RT. After 1 h the reaction was complete based on TLC and was quenched with sat. aq. $NaHCO_3$ soln. (5 mL). The layers were separated, and the aqueous layer was extracted with DCM (3 × 3 mL). The combined organic phase was dried over anhydrous Na_2SO_4 and concentrated. The residue was purified by flash column chromatography (EtOAc) to give bipolarolide D (103 mg, 0.269 mmol, 99%).

Appearance: colorless viscous oil.

R_f (SiO_2, EtOAc, $KMnO_4$, UV): 0.29.

FTIR (thin film, neat, cm^{-1}): 3425, 2922, 2853, 1680, 1617, 1456, 1376, 1301, 1183, 996, 804, 623.

^{1}H NMR (400 MHz, CD_3OD) δ 5.86 – 5.81 (m, 1H), 5.74 – 5.70 (m, 1H), 5.43 – 5.37 (m, 1H), 5.25 (t, *J* = 2.4 Hz, 1H), 3.96 – 3.83 (m, 2H), 3.92 (s, 2H), 3.45 – 3.38 (m, 1H), 2.45 – 2.31 (m, 2H), 2.23 – 2.15 (m, 1H), 2.15 (dd, *J* = 16.4, 2.6 Hz, 1H), 2.10 (t, *J* = 1.1 Hz, 3H), 2.08 – 1.92 (m, 2H), 1.99 (dd, *J* = 16.4, 2.4 Hz, 2H), 1.89 (dd, *J* = 12.7, 9.0 Hz, 1H), 1.73 (dd, *J* = 12.7, 7.2 Hz, 1H), 1.67 (s, 3H), 1.40 (dddd, *J* = 13.9, 10.7, 6.3, 3.4 Hz, 1H), 1.20 (dtd, *J* = 13.3, 10.2, 5.1 Hz, 1H), 1.07 (s, 3H), 1.01 (d, *J* = 6.8 Hz, 3H).

^{13}C{^{1}H} NMR (126 MHz, CD_3OD) δ 212.4, 183.2, 156.8, 145.7, 135.4, 129.8, 129.1, 127.4, 123.0, 78.8, 75.7, 69.2, 59.9, 57.7, 56.4, 44.9, 42.0, 39.2, 36.9, 32.7, 27.0, 23.7, 23.3, 17.9, 13.8.

HRMS (ESI, *m/z*): calc. for $C_{25}H_{34}O_3Na$ $[M+Na]^+$: 405.2400, found: 405.2401.

$[\alpha]_D^{25}$ = –-89.1 (c = 0.493, MeOH).

Comparison of NMR spectra of natural and synthetic samples of Bipolarolide D

^{1}H NMR

Natural Bipolarolide D<br>400 MHz<br>δ_H (*J*), ppm (Hz)	Synthetic Bipolarolide D<br>400 MHz<br>δ_H (*J*), ppm (Hz)	$\Delta\delta_{H,}$ ppm
5.84, m, 1H	5.84, m, 1H	0.00
5.72 (1.4), t, 1H	5.72, m, 1H	0.00
5.40, m, 1H	5.40, m, 1H	0.00
5.25 (2.4), t, 1H	5.25 (2.4), t, 1H	0.00
3.92, s, 1H	3.92, s, 2H	0.00
3.90, m, 2H	3.90, m, 2H	0.00
3.42, m, 1H	3.42, m, 1H	0.00
2.38, m, 2H	2.38, m, 2H	0.00
2.18, m, 1H	2.19, m, 1H	+0.01
2.15 (16.4, 2.5), dd, 1H	2.15 (16.4, 2.6), dd, 1H	0.00
2.10 (1.4), t, 3H	2.10 (1.1), t, 3H	0.00
2.01, m, 2H	2.00, m, 2H	-0.01
1.99 (16.4, 2.4), dd, 1H	1.99 (16.4, 2.4), dd, 1H	0.00
1.89 (12.7, 8.9), dd, 1H	1.89 (12.7, 9.0), dd, 1H	0.00
1.73 (12.7, 7.2), dd, 1H	1.73 (12.7, 7.2), dd, 1H	0.00
1.66, s, 3H	1.67, s, 3H	+0.01
1.40, m, 1H	1.40 (13.9, 10.7, 6.3, 3.4), dddd, 1H	0.00
1.21, m, 1H	1.20 (13.3, 10.2, 5.1), dtd, 1H	-0.01
1.07, s, 3H	1.07, s, 3H	0.00
1.01 (6.8), d, 3H	1.01 (6.8), d, 3H	0.00

^{13}C NMR

Natural Bipolarolide D<br>101 MHz<br>δ_C, ppm	Synthetic Bipolarolide D<br>126 MHz<br>δ_C, ppm	$\Delta\delta_C$, ppm
212.5	212.4	-0.1
183.2	183.2	0.0
156.8	156.8	0.0
145.7	145.7	0.0
135.4	135.4	0.0
129.8	129.8	0.0
129.2	129.1	-0.1
127.4	127.4	0.0
123.0	123.0	0.0
78.8	78.8	0.0
75.7	75.7	0.0
69.2	69.2	0.0
59.9	59.9	0.0
57.7	57.7	0.0
56.4	56.4	0.0
44.9	44.9	0.0
42.0	42.0	0.0
39.2	39.2	0.0
36.9	36.9	0.0
32.7	32.7	0.0
27.0	27.0	0.0
23.7	23.7	0.0
23.2	23.3	+0.1
17.9	17.9	0.0
13.8	13.8	0.0

NMR Spectra

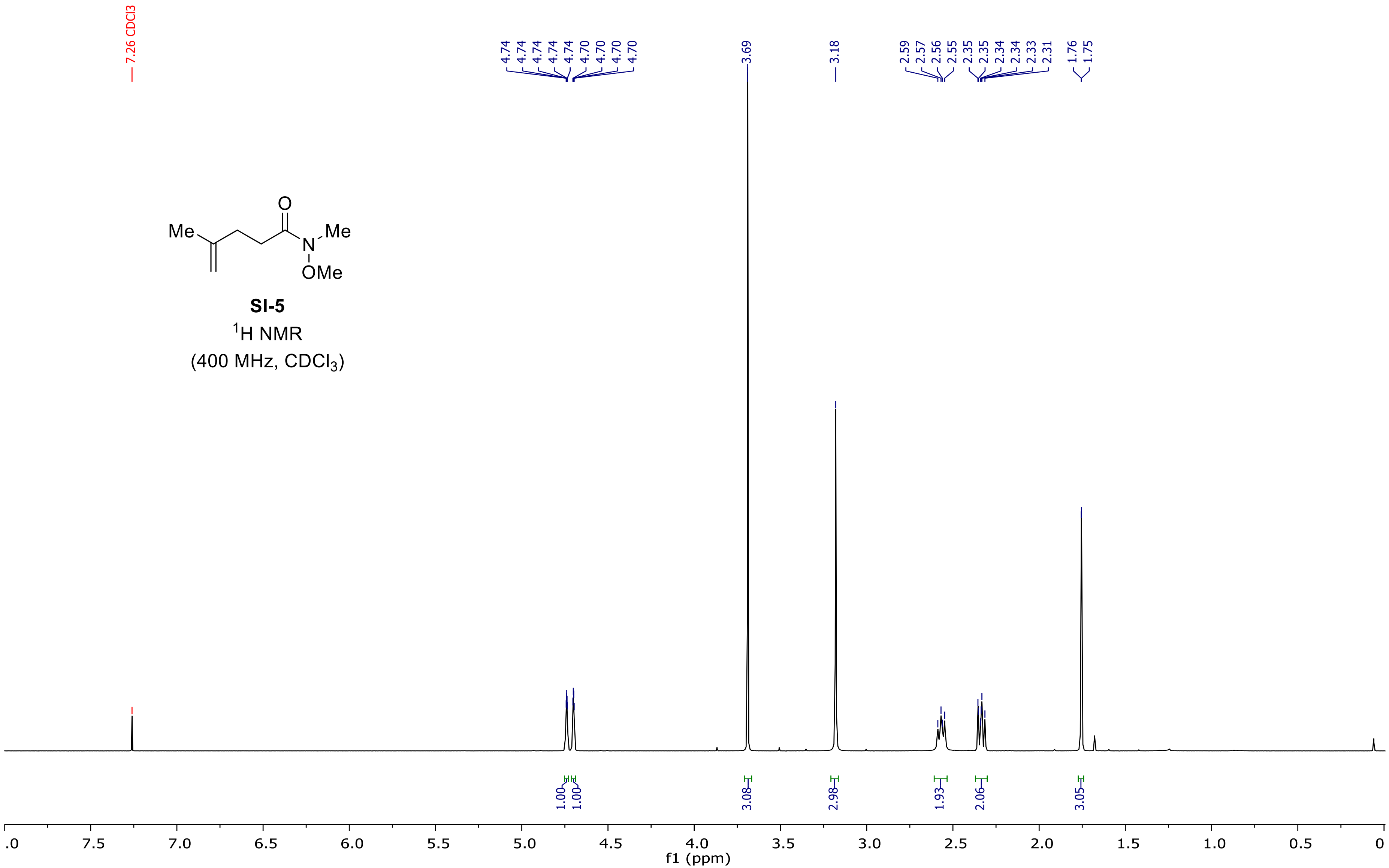
7.26 CDCl3
4.74
4.74
4.74
4.74
4.74
4.70
4.70
4.70
4.70
3.69
3.18
2.59
2.57
2.56
2.55
2.35
2.35
2.34
2.34
2.33
2.31
1.76
1.75
Me
O
N
Me
OMe
SI-5
1H NMR
(400 MHz, CDCl3)
1.00
1.00
3.08
2.98
1.93
2.06
3.05
f1 (ppm)

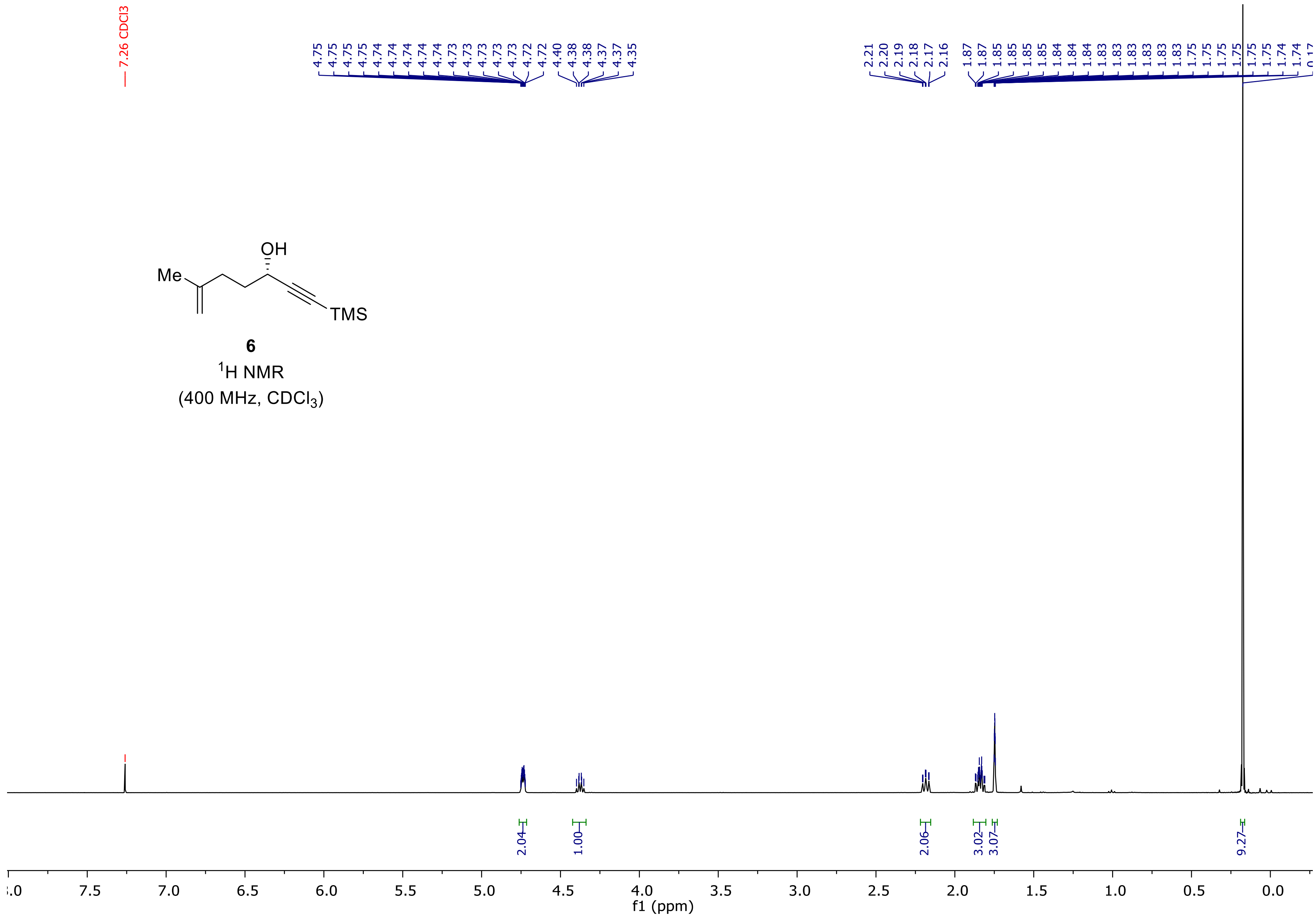
7.26 CDCl3
4.75
4.75
4.75
4.75
4.74
4.74
4.74
4.74
4.73
4.73
4.73
4.73
4.73
4.72
4.72
4.40
4.38
4.38
4.37
4.37
4.35
2.21
2.20
2.19
2.18
2.17
2.16
1.87
1.87
1.85
1.85
1.85
1.85
1.84
1.84
1.84
1.83
1.83
1.83
1.83
1.83
1.83
1.75
1.75
1.75
1.75
1.75
1.74
OH
Me
TMS
6
1H NMR
(400 MHz, CDCl3)
2.04
1.00
2.06
3.02
3.07
9.27
7.5
7.0
6.5
6.0
5.5
5.0
4.5
4.0
3.5
3.0
2.5
2.0
1.5
1.0
0.5
0.0
f1 (ppm)

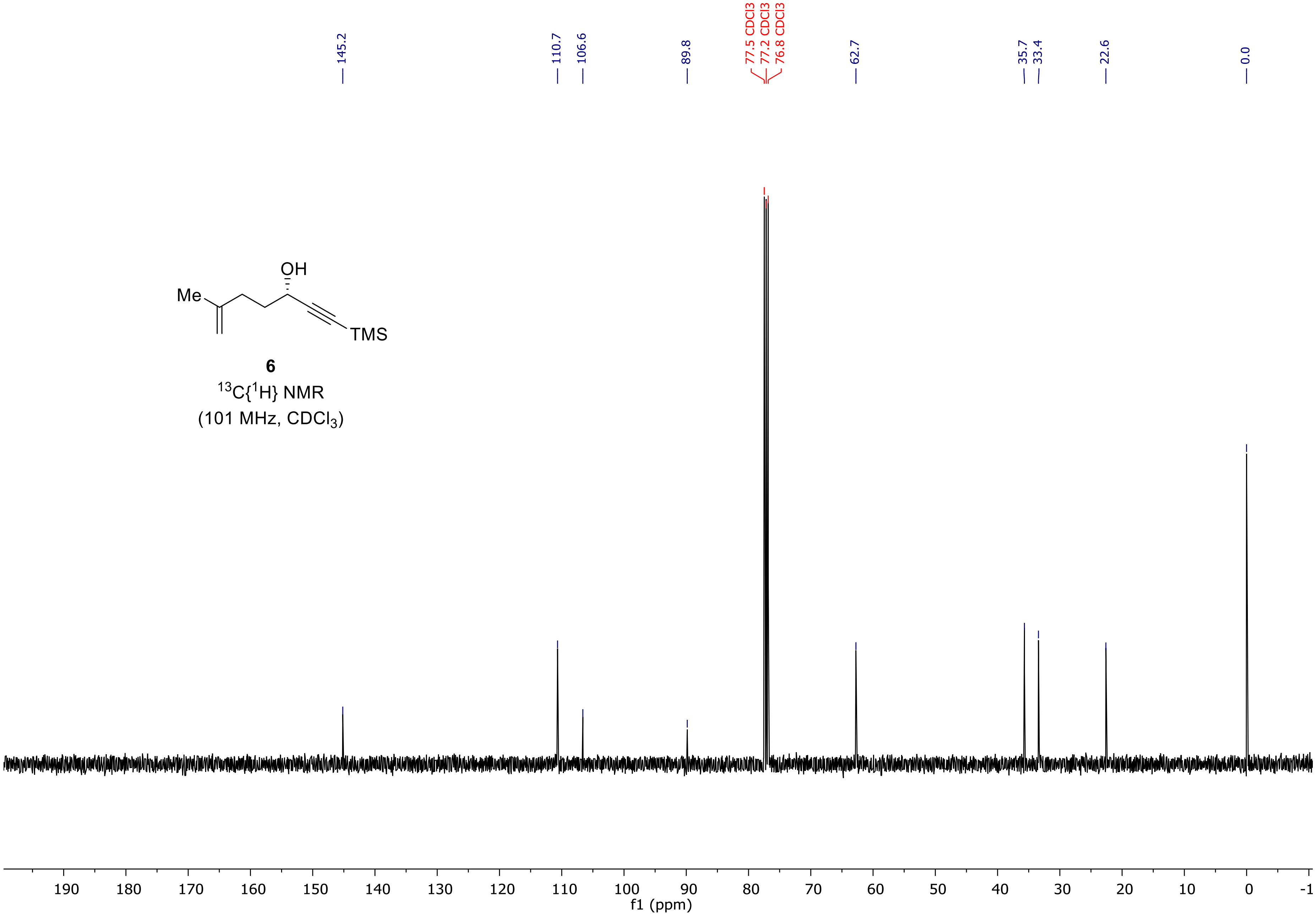

6

$^{13}C\{^{1}H\}$ NMR

(101 MHz, $CDCl_3$)

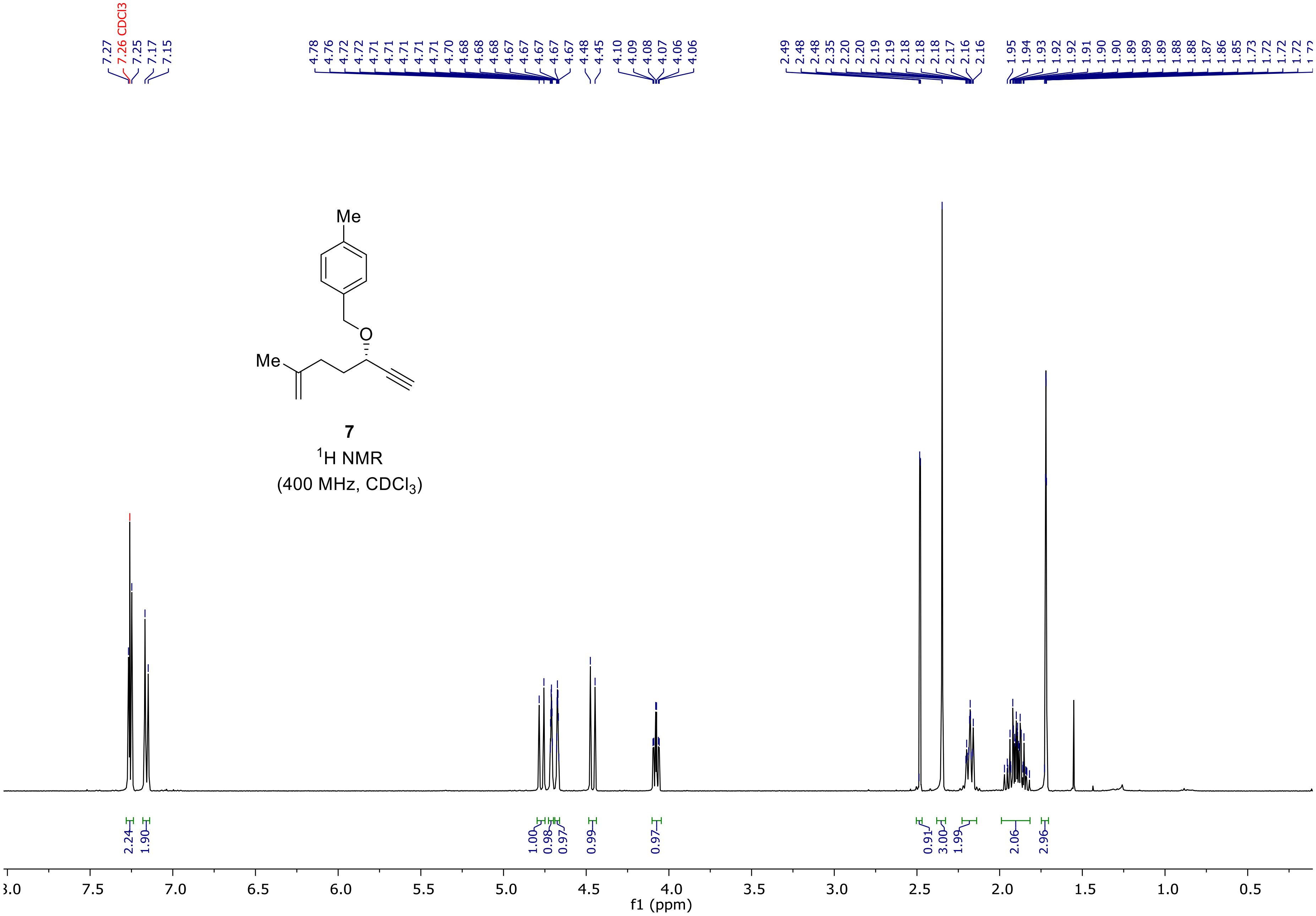
7.27
7.26 CDCl3
7.25
7.17
7.15
4.78
4.76
4.72
4.72
4.71
4.71
4.71
4.71
4.70
4.68
4.68
4.67
4.67
4.67
4.67
4.67
4.48
4.45
4.10
4.09
4.08
4.07
4.06
4.06
2.49
2.48
2.48
2.35
2.20
2.20
2.19
2.19
2.18
2.18
2.18
2.17
2.16
2.16
1.95
1.94
1.93
1.92
1.92
1.91
1.90
1.90
1.89
1.89
1.89
1.88
1.88
1.87
1.86
1.85
1.73
1.72
1.72
Me
O
Me
7
1H NMR
(400 MHz, CDCl3)
2.24
1.90
1.00
0.98
0.97
0.99
0.97
0.91
3.00
1.99
2.06
2.96
7.5
7.0
6.5
6.0
5.5
5.0
4.5
4.0
3.5
3.0
2.5
2.0
1.5
1.0
0.5
f1 (ppm)

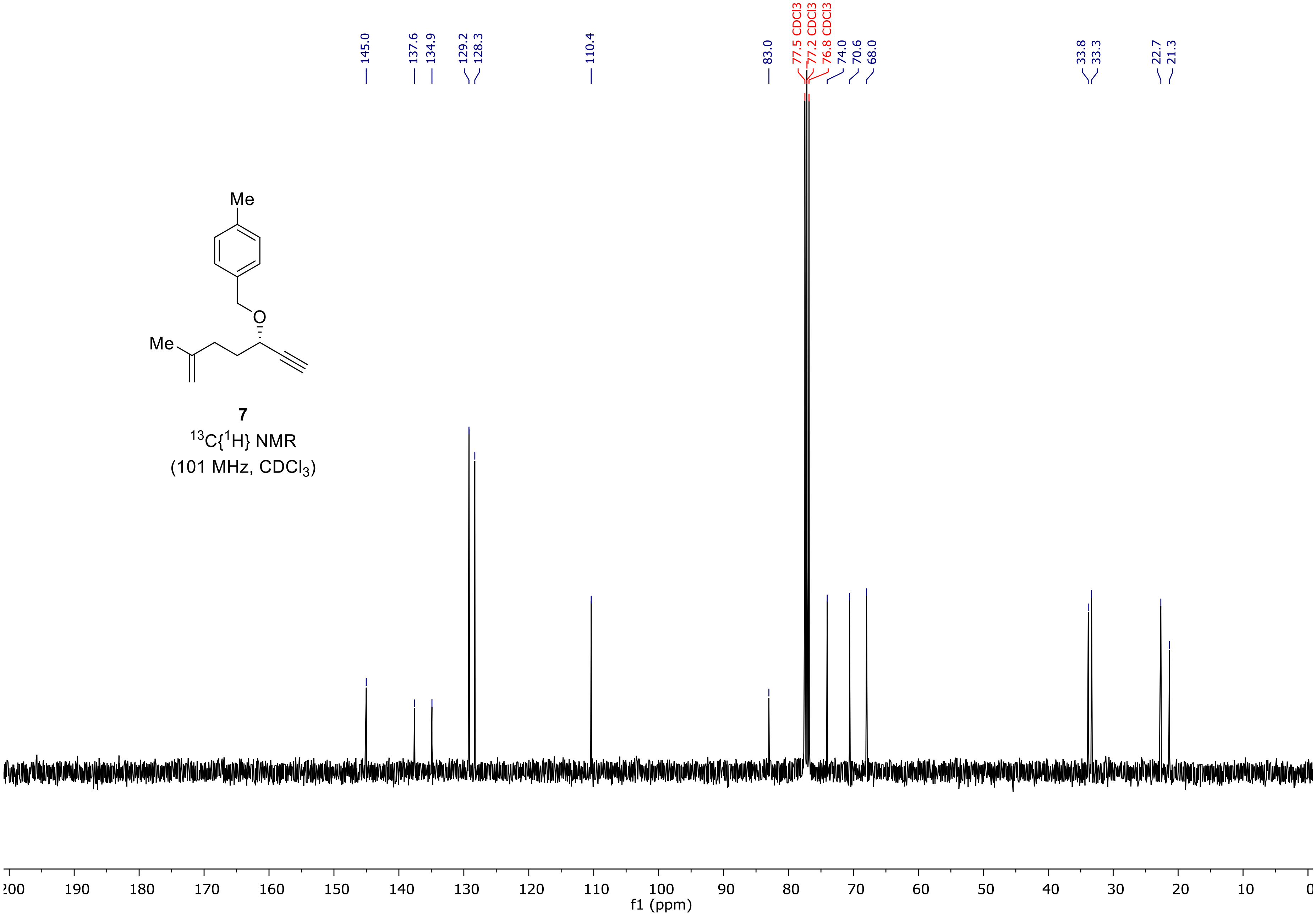

7

$^{13}C\{^{1}H\}$ NMR

(101 MHz, $CDCl_3$)

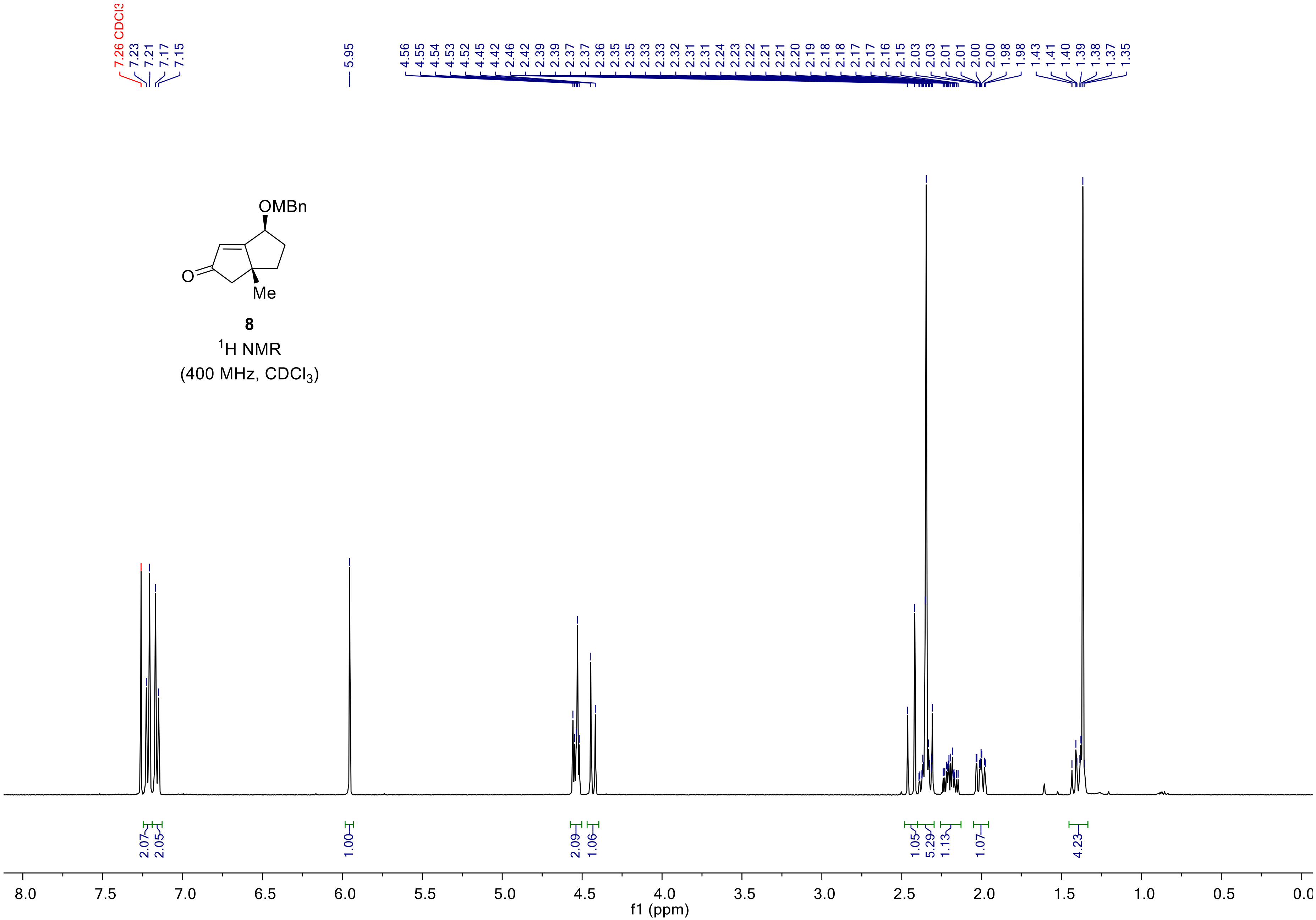
7.26 CDCl3
7.23
7.21
7.17
7.15
5.95
4.56
4.55
4.54
4.53
4.52
4.45
4.42
2.46
2.42
2.39
2.39
2.37
2.37
2.36
2.35
2.35
2.33
2.33
2.32
2.31
2.31
2.24
2.23
2.22
2.21
2.21
2.20
2.19
2.18
2.18
2.17
2.17
2.16
2.15
2.03
2.03
2.01
2.01
2.00
2.00
1.98
1.98
1.43
1.41
1.40
1.39
1.38
1.37
1.35
OMBn
O
Me
8
1H NMR
(400 MHz, CDCl3)
2.07
2.05
1.00
2.09
1.06
1.05
5.29
1.13
1.07
4.23
f1 (ppm)

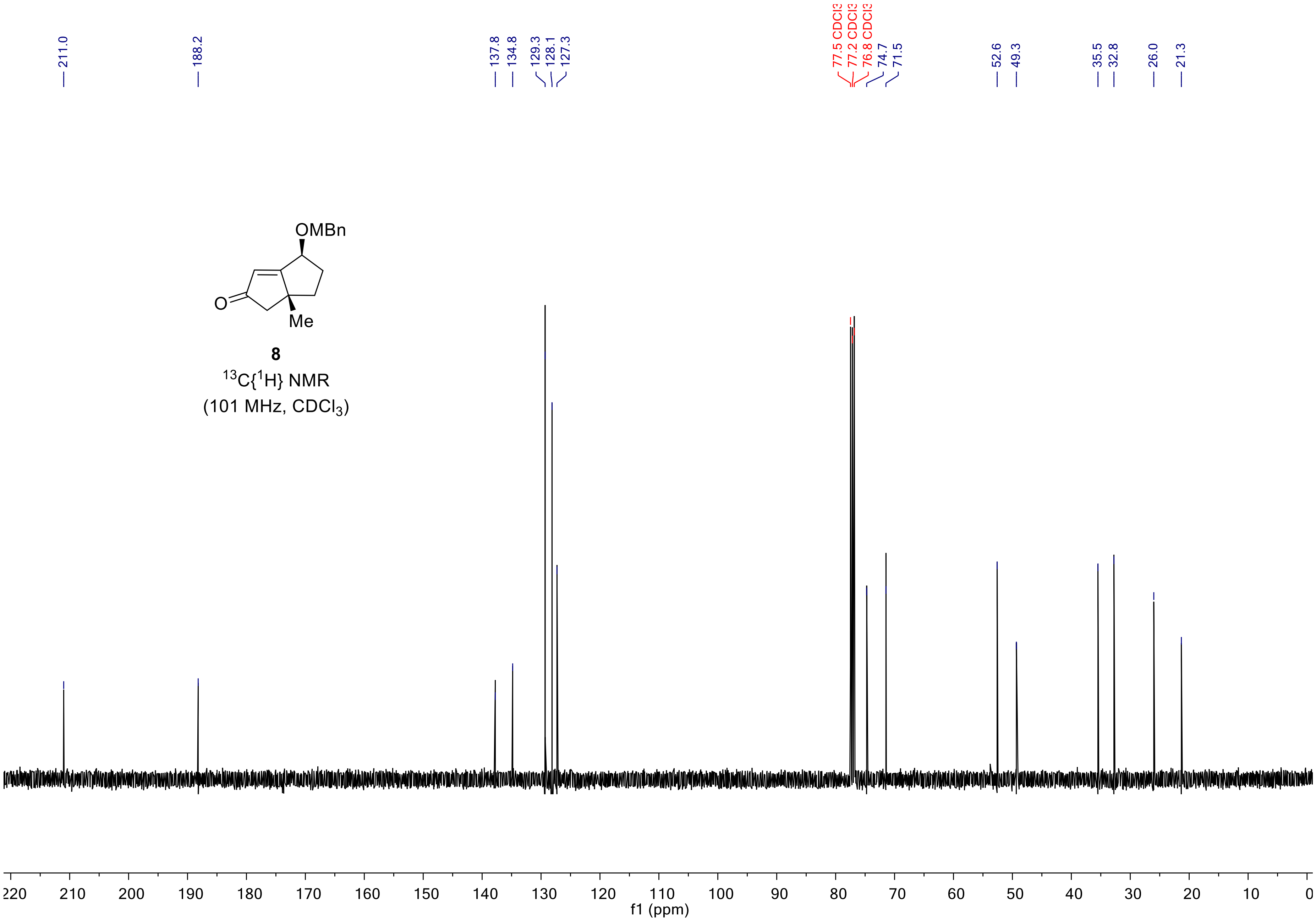
211.0
188.2
137.8
134.8
129.3
128.1
127.3
77.5 CDCl3
77.2 CDCl3
76.8 CDCl3
74.7
71.5
52.6
49.3
35.5
32.8
26.0
21.3
OMBn
O
Me
8
$^{13}C\{^1H\}$ NMR
(101 MHz, $CDCl_3$)
220 210 200 190 180 170 160 150 140 130 120 110 100 90 80 70 60 50 40 30 20 10 0
f1 (ppm)

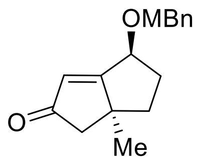

SI-6

^{1}H NMR

(400 MHz, $CDCl_3$)

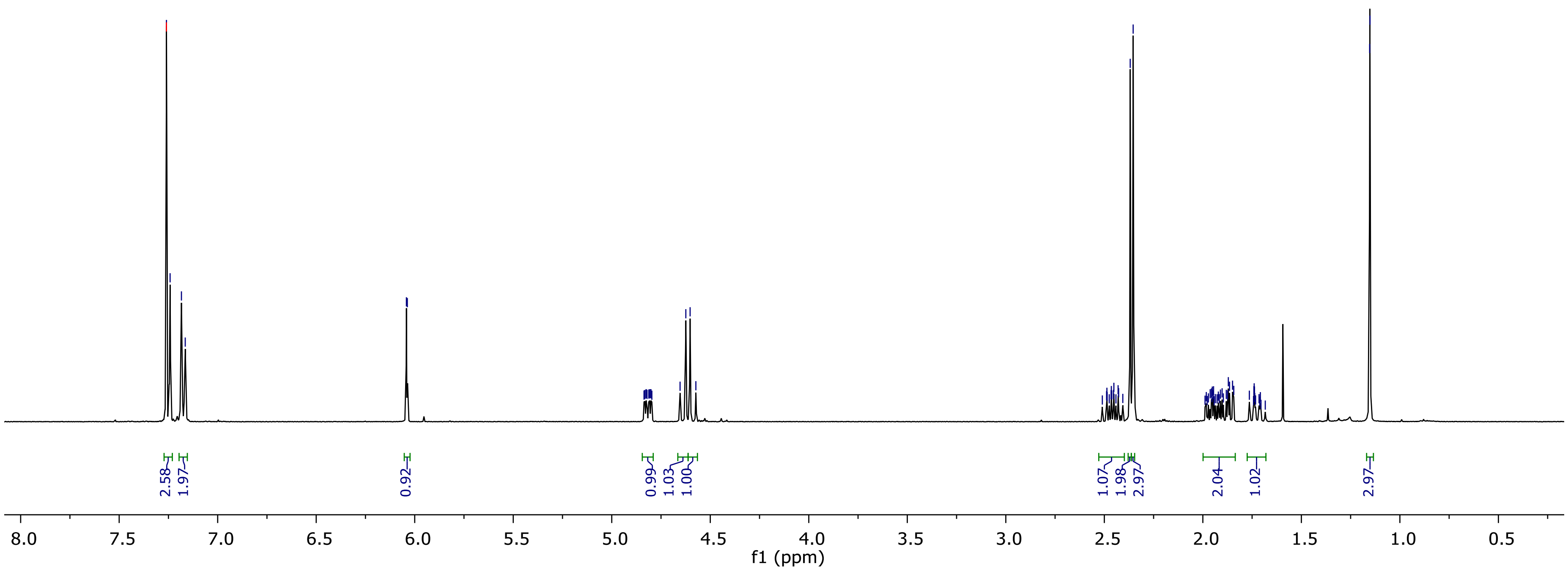

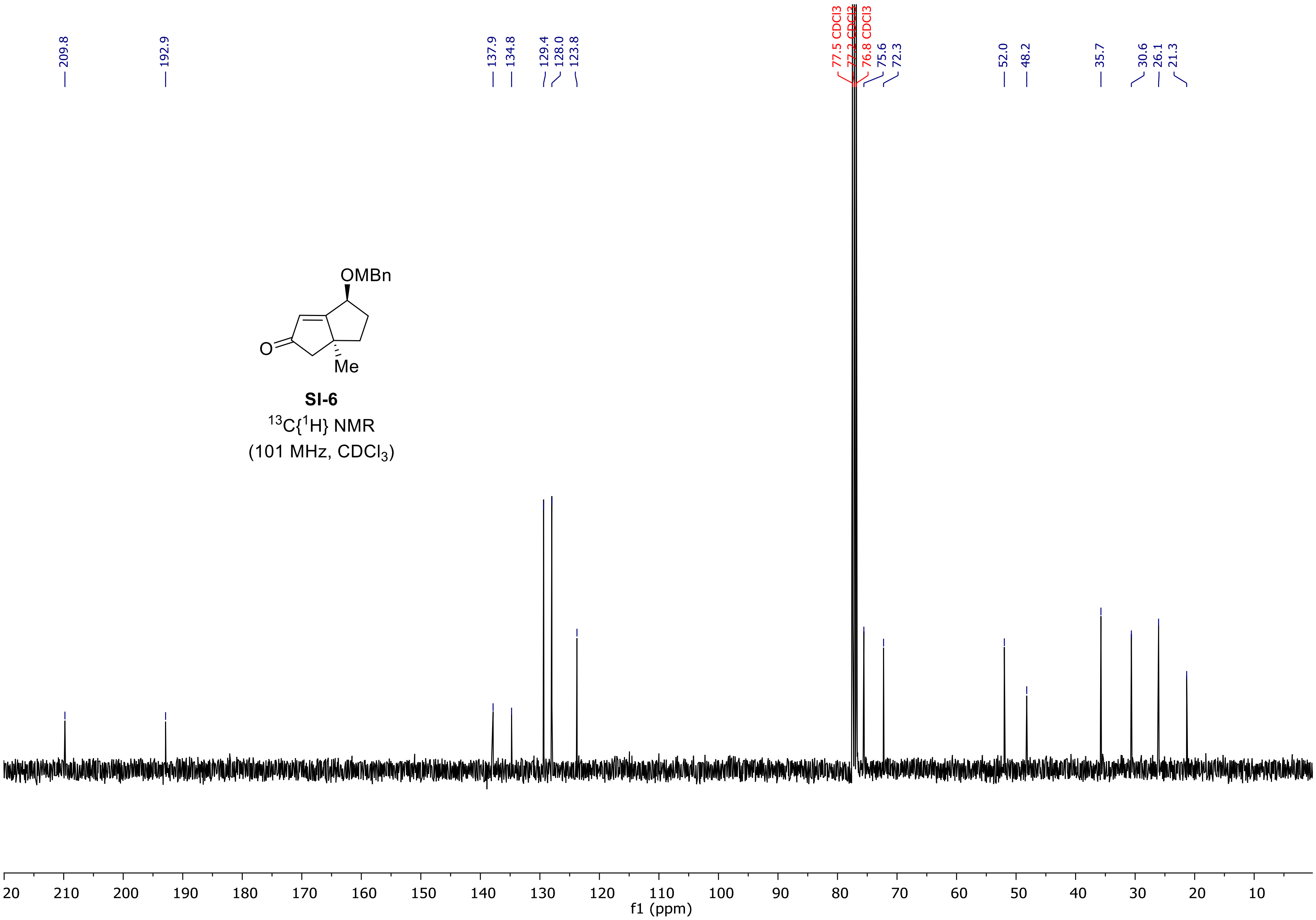

SI-6

$^{13}C\{^1H\}$ NMR

(101 MHz, $CDCl_3$)

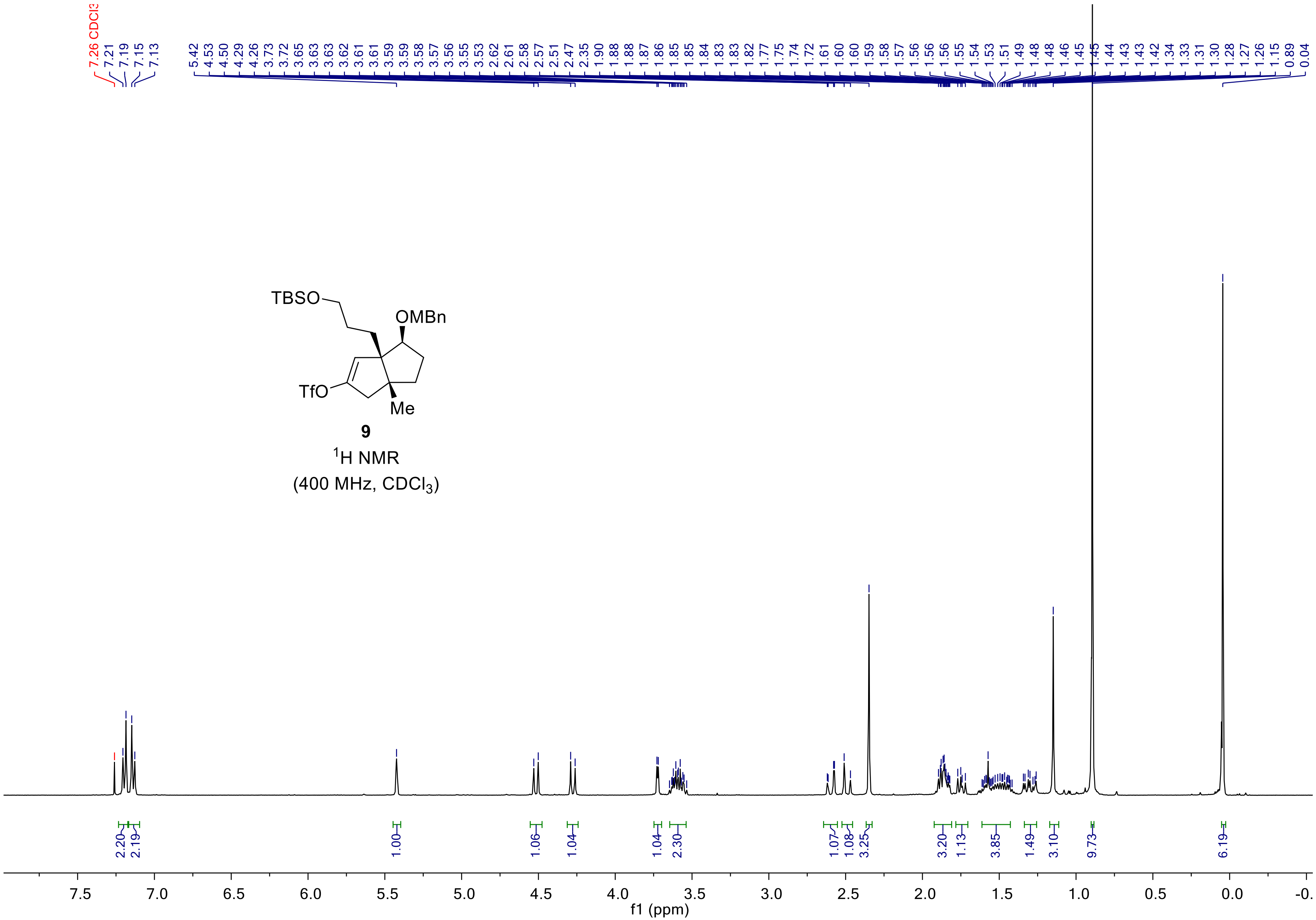

9

^{1}H NMR

(400 MHz, $CDCl_3$)

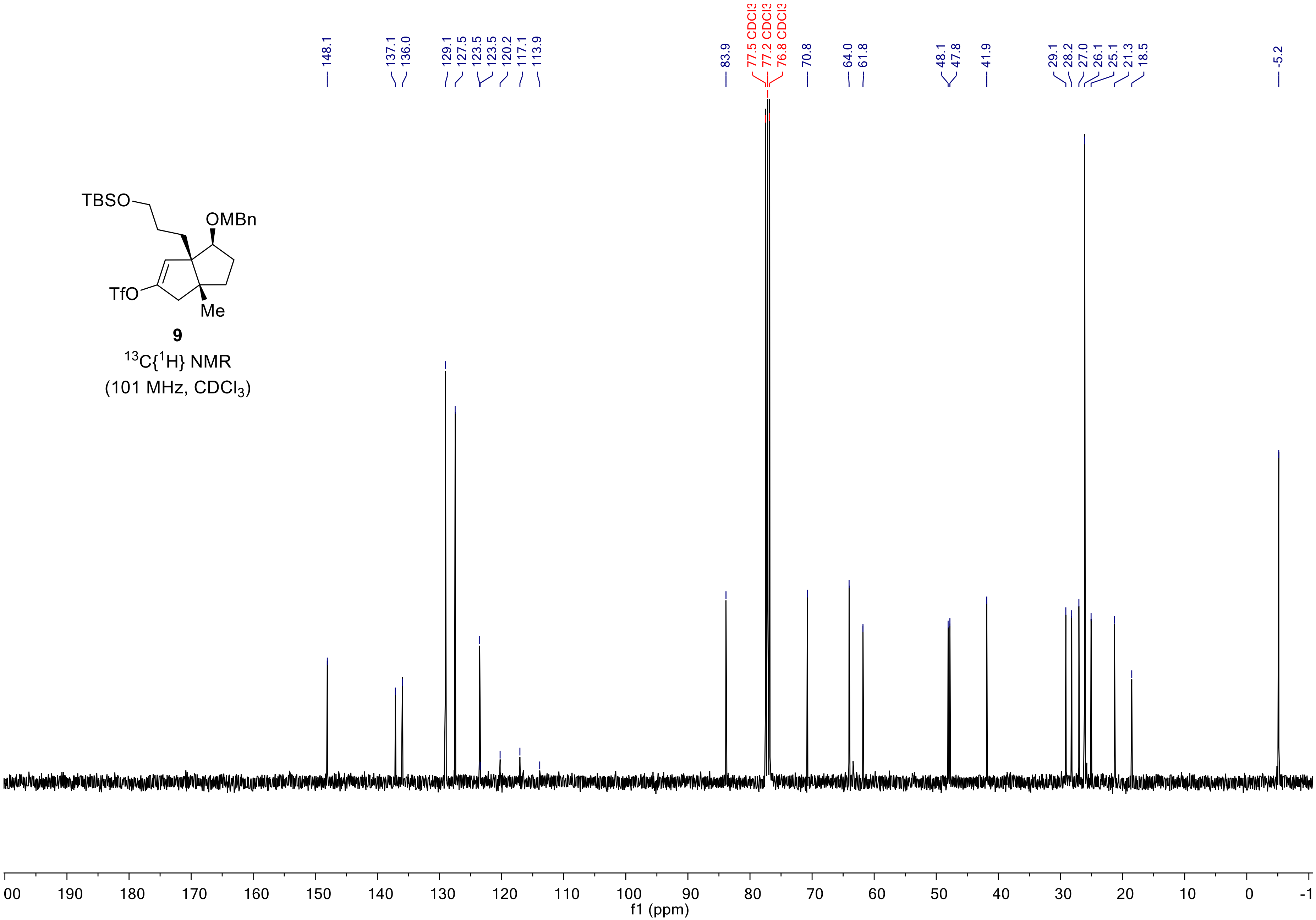
148.1
137.1
136.0
129.1
127.5
123.5
123.5
120.2
117.1
113.9
83.9
77.5 CDCl3
77.2 CDCl3
76.8 CDCl3
70.8
64.0
61.8
48.1
47.8
41.9
29.1
28.2
27.0
26.1
25.1
21.3
18.5
-5.2
TBSO
OMBn
TfO
Me
9
13C{1H} NMR
(101 MHz, CDCl3)
00
190
180
170
160
150
140
130
120
110
100
90
80
70
60
50
40
30
20
10
0
-1
f1 (ppm)

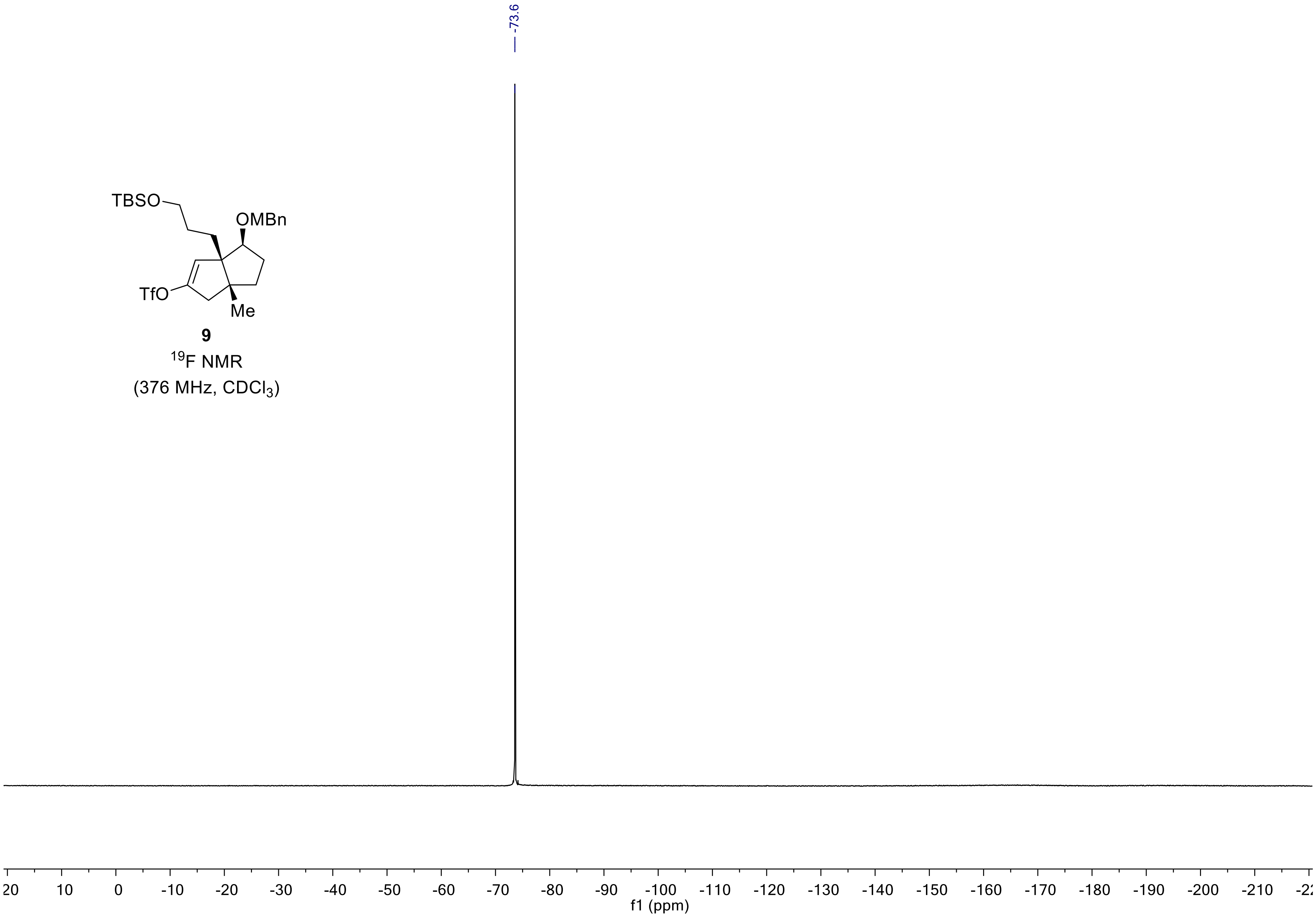
-73.6
TBSO
OMBn
TfO
Me
9
19F NMR
(376 MHz, CDCl3)
20 10 0 -10 -20 -30 -40 -50 -60 -70 -80 -90 -100 -110 -120 -130 -140 -150 -160 -170 -180 -190 -200 -210
f1 (ppm)

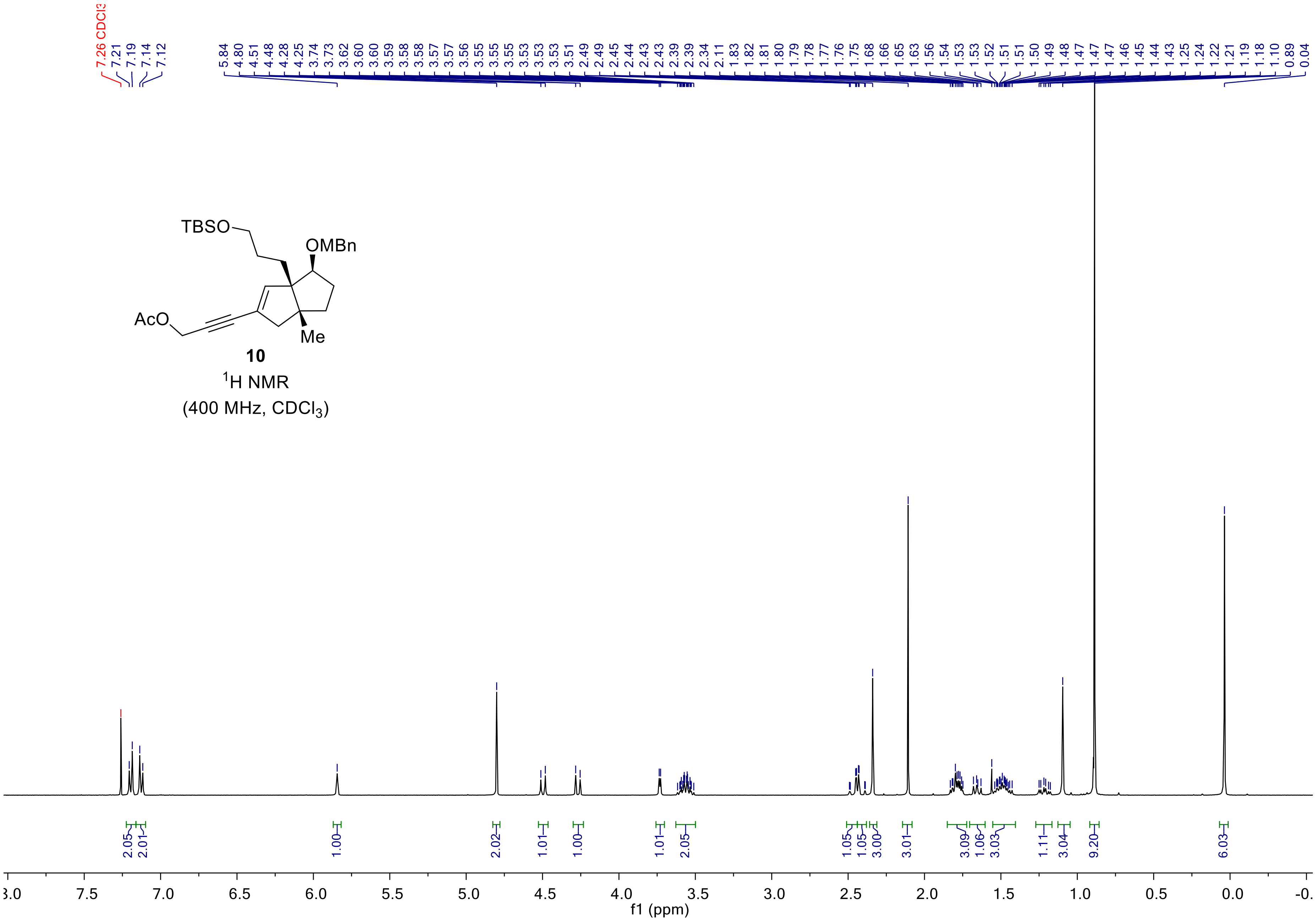
TBSO
OMBn
AcO
Me
10
^{1}H NMR
(400 MHz, $CDCl_3$)
f1 (ppm)

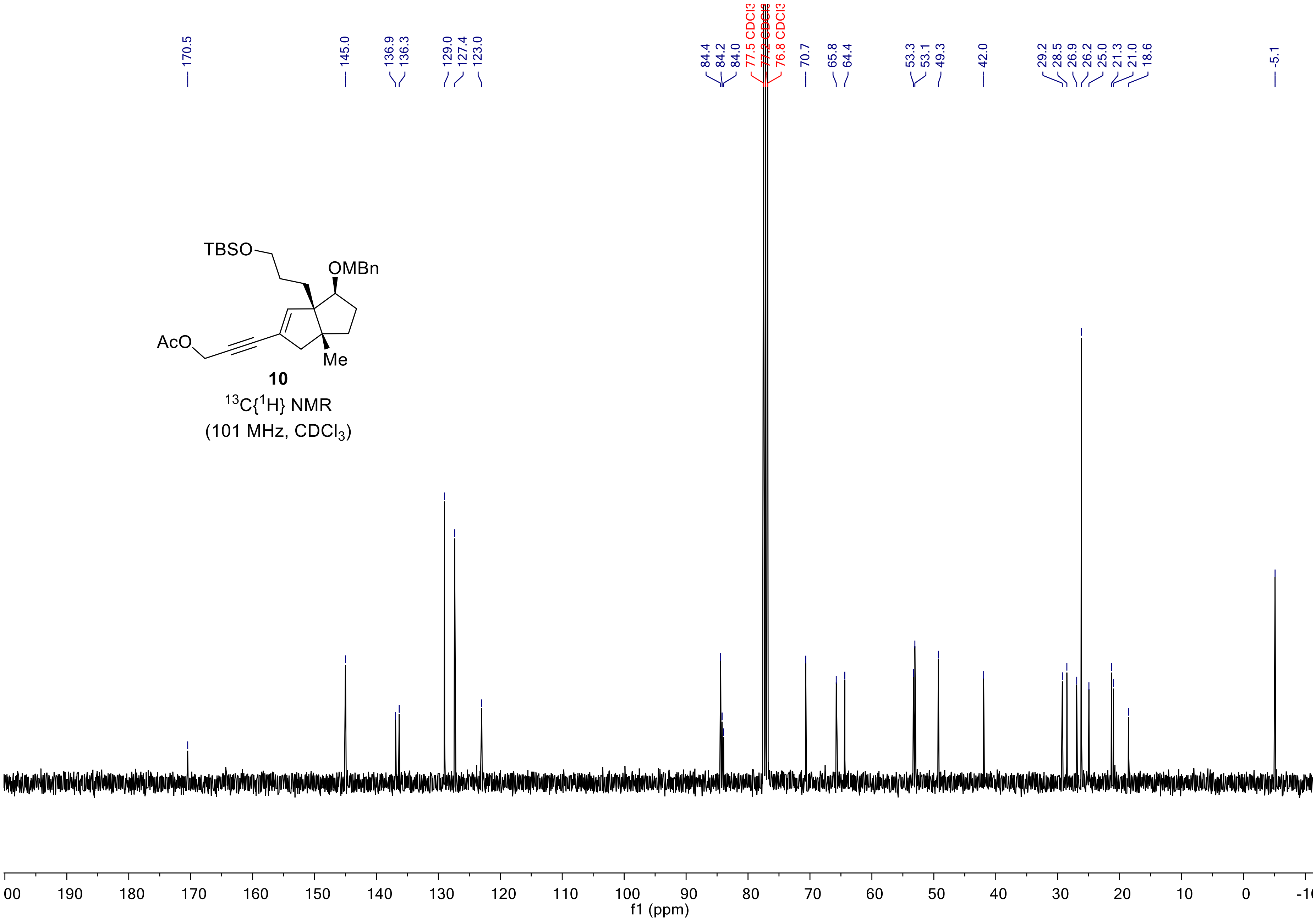
170.5
145.0
136.9
136.3
129.0
127.4
123.0
84.4
84.2
84.0
77.5 CDCl3
77.1 CDCl3
76.8 CDCl3
70.7
65.8
64.4
53.3
53.1
49.3
42.0
29.2
28.5
26.9
26.2
25.0
21.3
21.0
18.6
-5.1
TBSO
OMBn
AcO
Me
10
$^{13}C\{^1H\}$ NMR
(101 MHz, $CDCl_3$)
f1 (ppm)

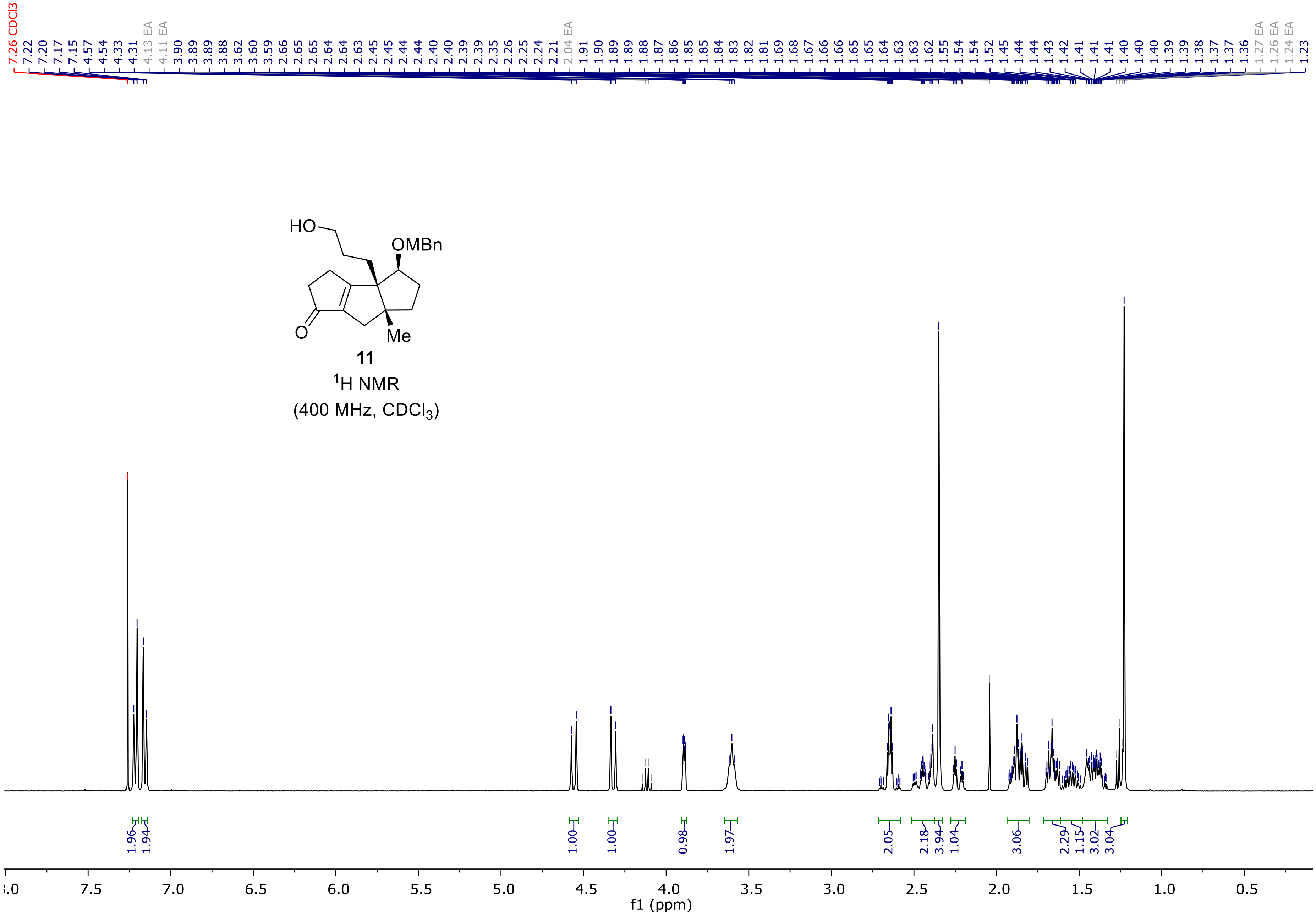

7.26 CDCl3
7.22 7.20 7.17 7.15 4.57 4.54 4.33 4.31
4.13 EA 4.11 EA
3.90 3.89 3.89 3.88 3.62 3.60 3.59 2.66 2.65 2.65 2.64 2.64 2.63 2.45 2.45 2.44 2.44 2.40 2.40 2.39 2.39 2.35 2.26 2.25 2.24 2.21
2.04 EA
1.91 1.90 1.89 1.89 1.88 1.87 1.86 1.85 1.85 1.84 1.83 1.82 1.81 1.69 1.68 1.67 1.66 1.66 1.65 1.65 1.64 1.63 1.63 1.62 1.55 1.54 1.54 1.52 1.45 1.44 1.44 1.43 1.42 1.41 1.41 1.41 1.40 1.40 1.40 1.39 1.39 1.38 1.37 1.37 1.36
1.27 EA 1.26 EA 1.24 EA
1.23
HO
OMBn
O
Me
11
1H NMR
(400 MHz, CDCl3)
1.96 1.94 1.00 1.00 0.98 1.97 2.05 2.18 3.94 1.04 3.06 2.29 1.15 3.02 3.04
8.0 7.5 7.0 6.5 6.0 5.5 5.0 4.5 4.0 3.5 3.0 2.5 2.0 1.5 1.0 0.5
f1 (ppm)

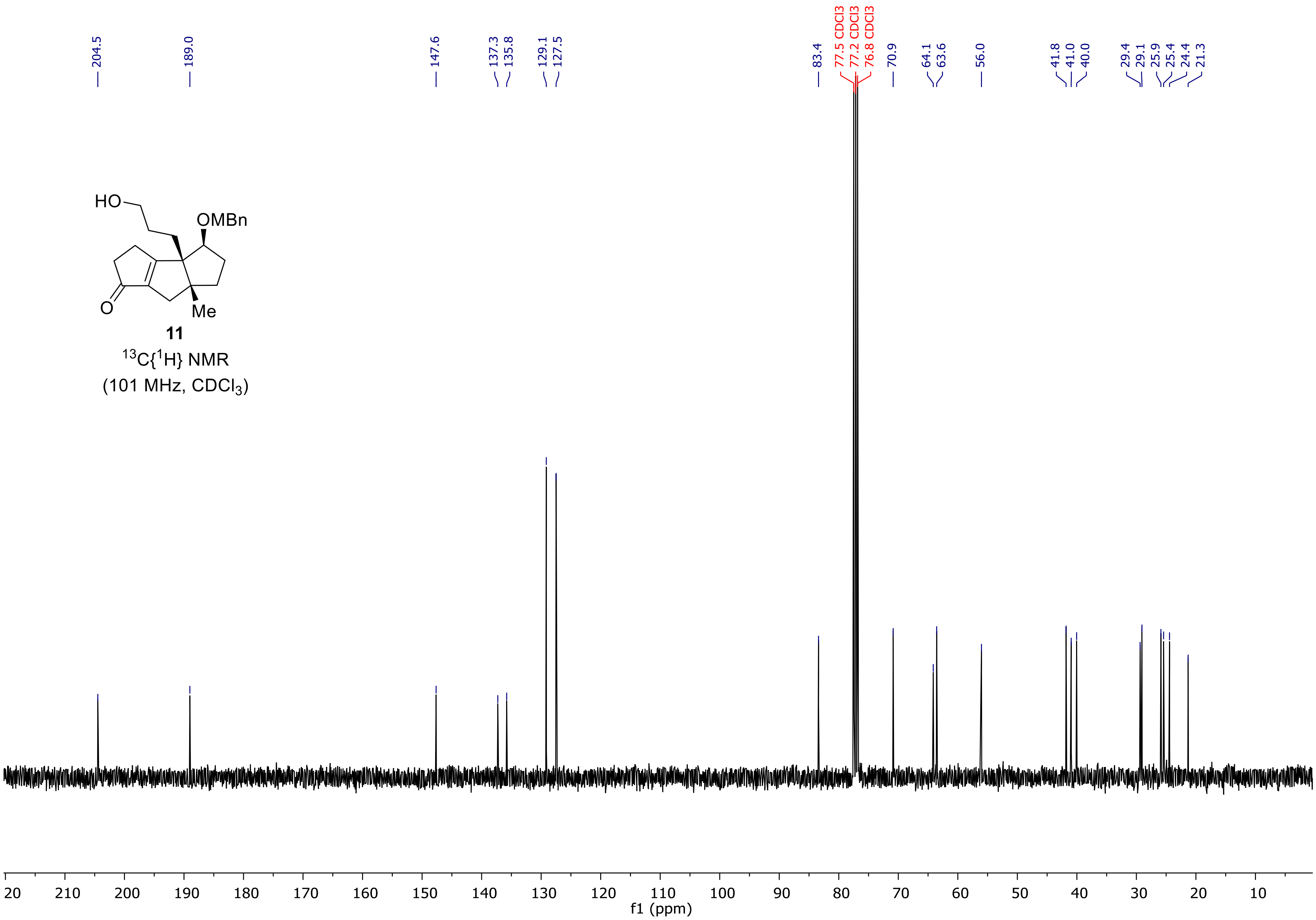

204.5
189.0
147.6
137.3
135.8
129.1
127.5
83.4
77.5 CDCl3
77.2 CDCl3
76.8 CDCl3
70.9
64.1
63.6
56.0
41.8
41.0
40.0
29.4
29.1
25.9
25.4
24.4
21.3
HO
OMBn
O
Me
11
$^{13}C\{^1H\}$ NMR
(101 MHz, $CDCl_3$)
f1 (ppm)

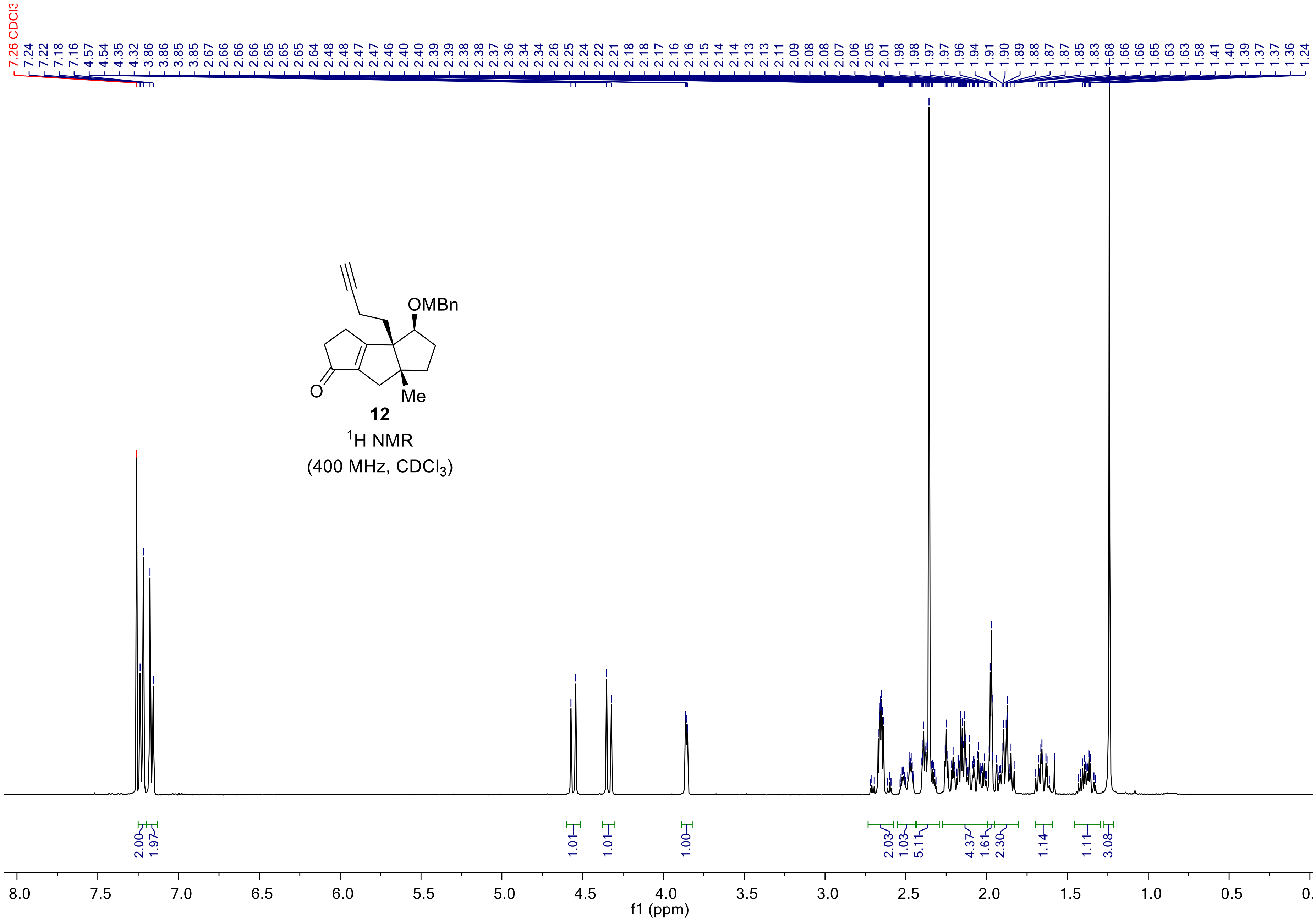

7.26 CDCl3
7.24 7.22 7.18 7.16 4.57 4.54 4.35 4.32 3.86 3.86 3.85 3.85 2.67 2.66 2.66 2.66 2.65 2.65 2.65 2.64 2.48 2.48 2.47 2.47 2.46 2.40 2.40 2.39 2.39 2.38 2.38 2.37 2.36 2.34 2.34 2.26 2.25 2.24 2.22 2.21 2.18 2.18 2.17 2.16 2.16 2.15 2.14 2.14 2.13 2.13 2.11 2.09 2.08 2.08 2.07 2.06 2.05 2.01 1.98 1.98 1.97 1.97 1.96 1.94 1.91 1.90 1.89 1.88 1.87 1.87 1.85 1.83 1.68 1.66 1.66 1.65 1.63 1.63 1.58 1.41 1.40 1.39 1.37 1.37 1.36 1.24
OMBn
O
Me
12
1H NMR
(400 MHz, CDCl3)
2.00 1.97 1.01 1.01 1.00 2.03 1.03 5.11 4.37 1.61 2.30 1.14 1.11 3.08
8.0 7.5 7.0 6.5 6.0 5.5 5.0 4.5 4.0 3.5 3.0 2.5 2.0 1.5 1.0 0.5 0.
f1 (ppm)

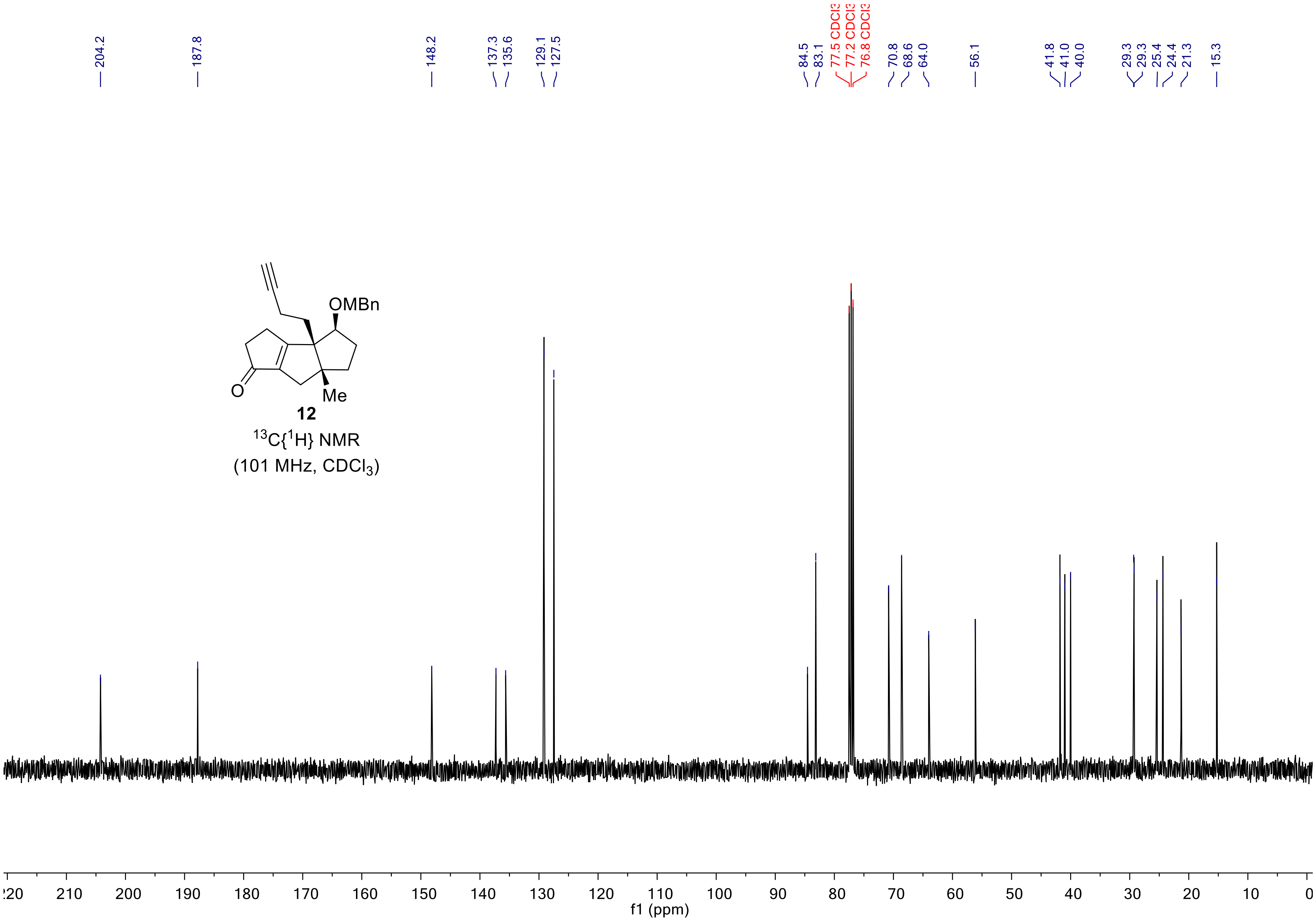
204.2
187.8
148.2
137.3
135.6
129.1
127.5
84.5
83.1
77.5 CDCl3
77.2 CDCl3
76.8 CDCl3
70.8
68.6
64.0
56.1
41.8
41.0
40.0
29.3
29.3
25.4
24.4
21.3
15.3
OMBn
O
Me
12
13C{1H} NMR
(101 MHz, CDCl3)
f1 (ppm)

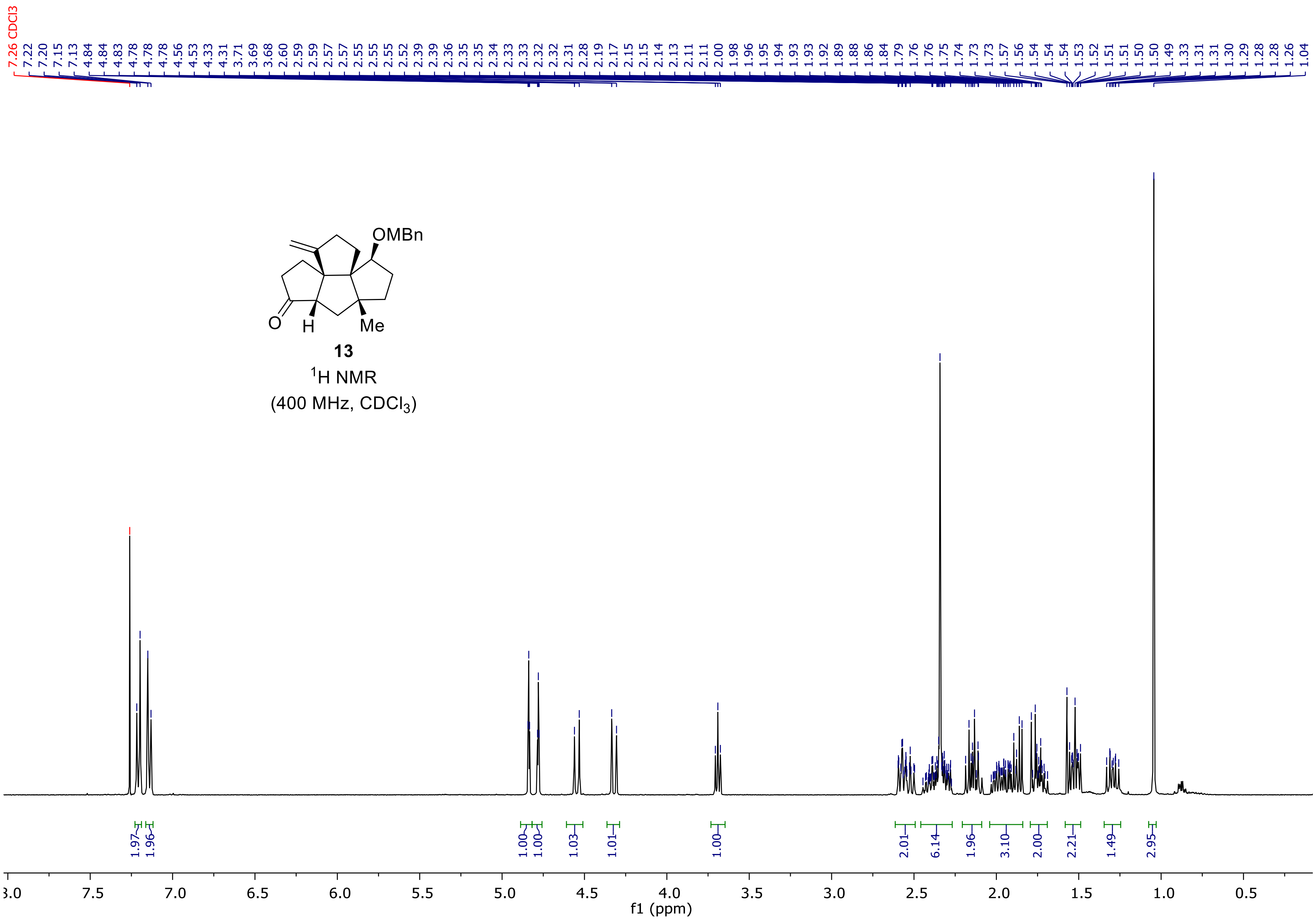
OMBn
O
H
Me
13
¹H NMR
(400 MHz, CDCl3)
f1 (ppm)

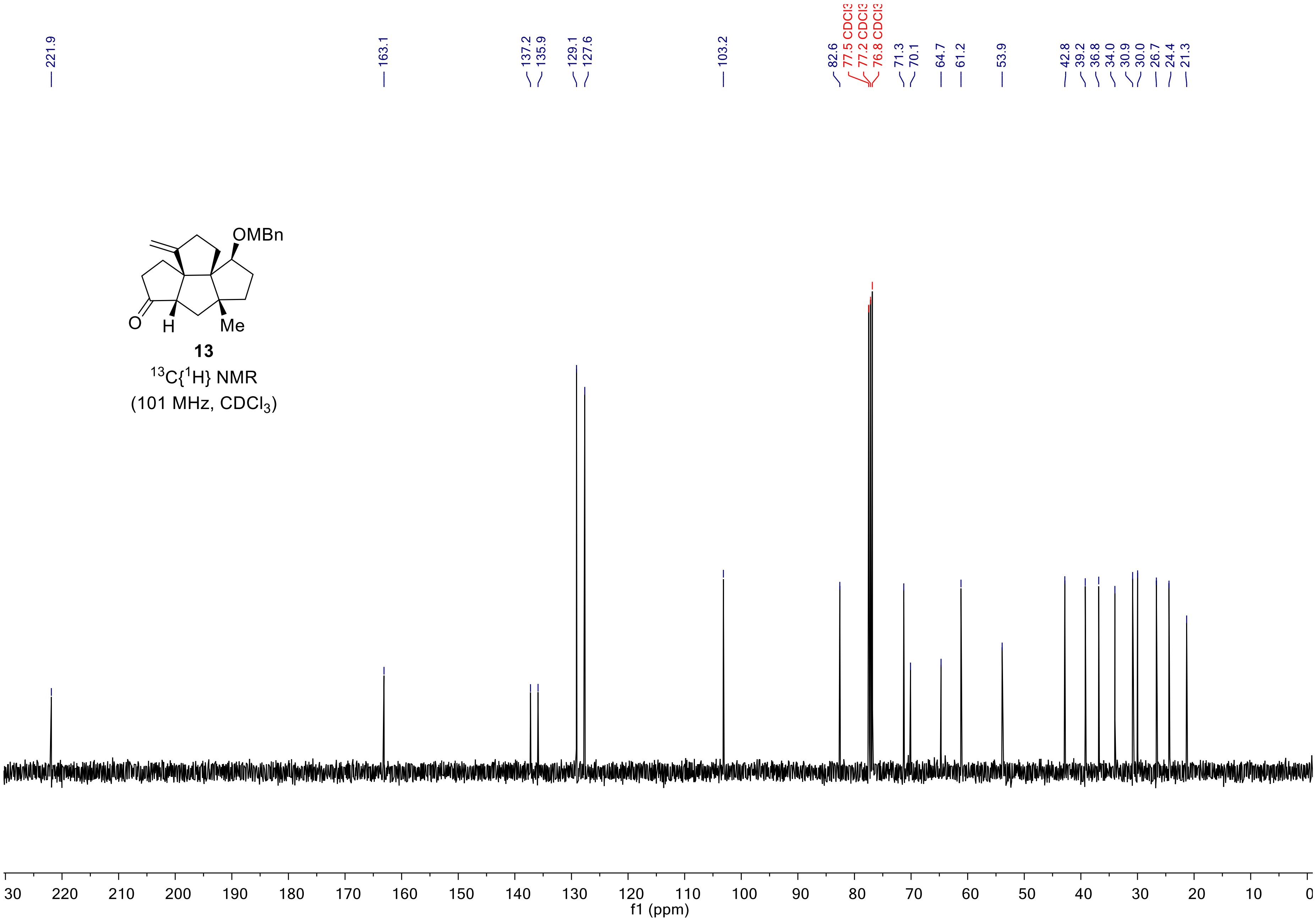

13

$^{13}C\{^{1}H\}$ NMR

(101 MHz, $CDCl_3$)

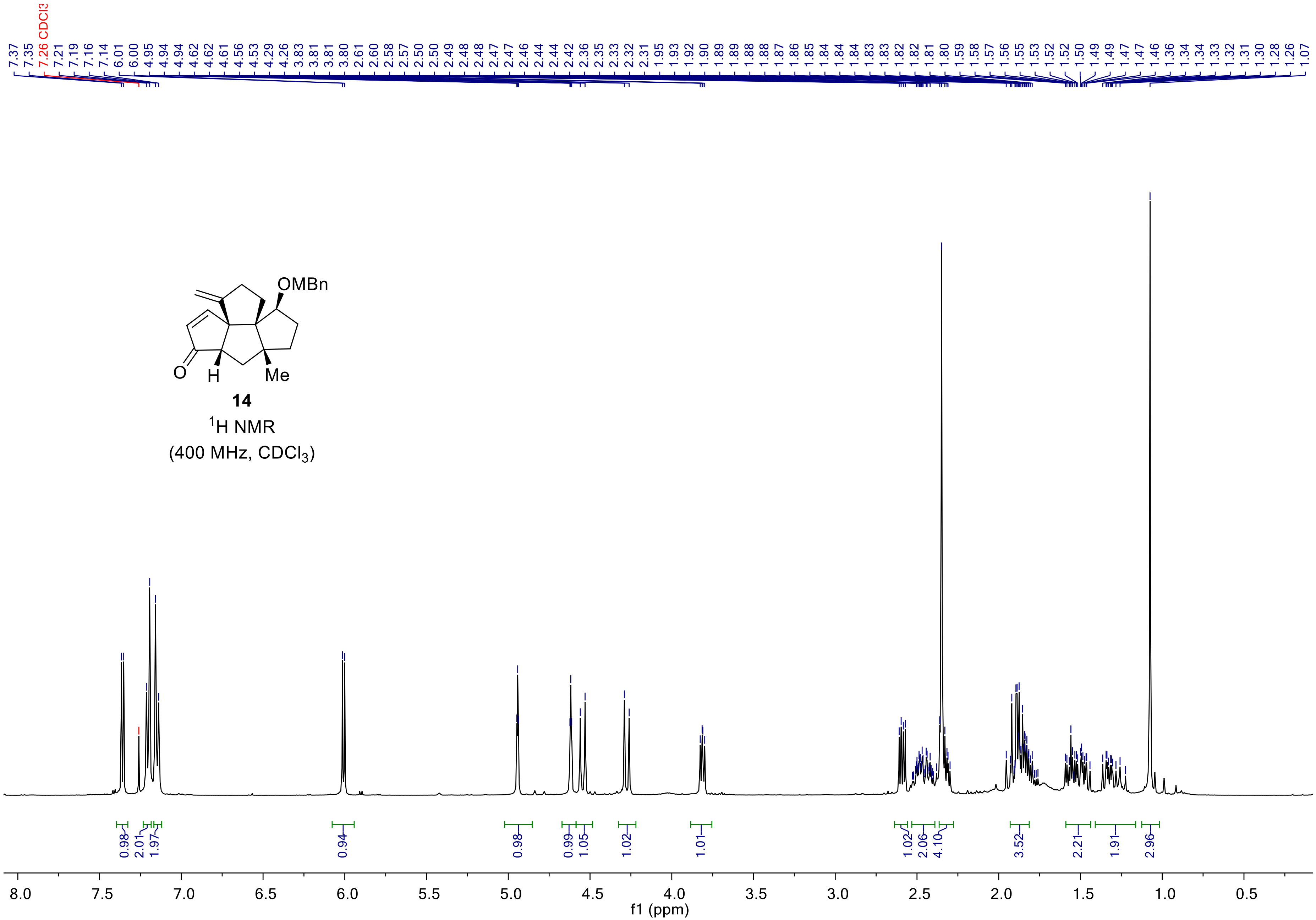

14
^{1}H NMR
(400 MHz, $CDCl_3$)

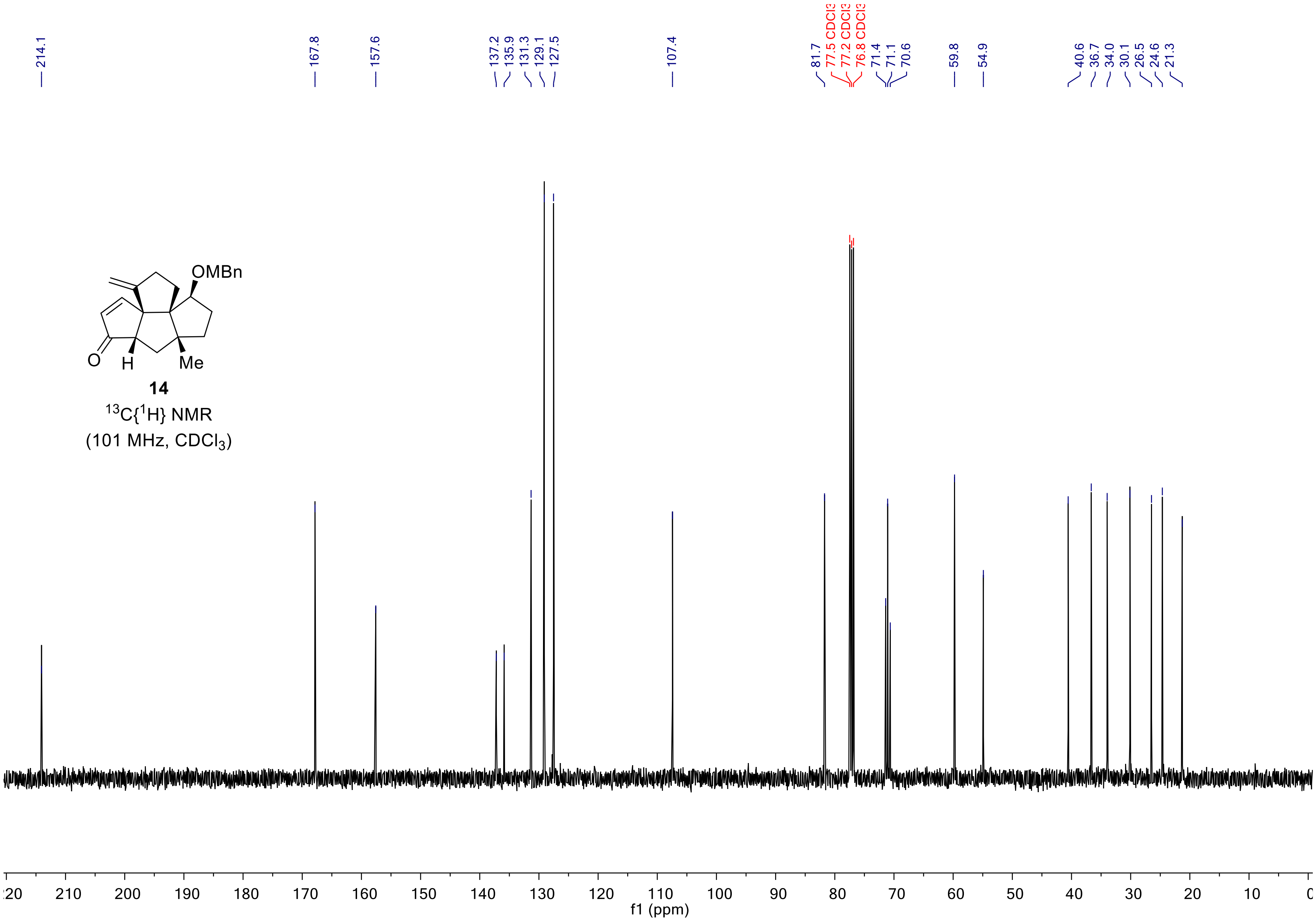

14

$^{13}C\{^{1}H\}$ NMR

(101 MHz, $CDCl_3$)

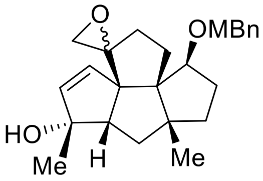

15

^{1}H NMR

(400 MHz, $CDCl_3$)

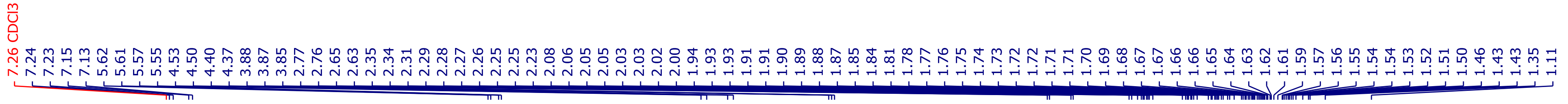

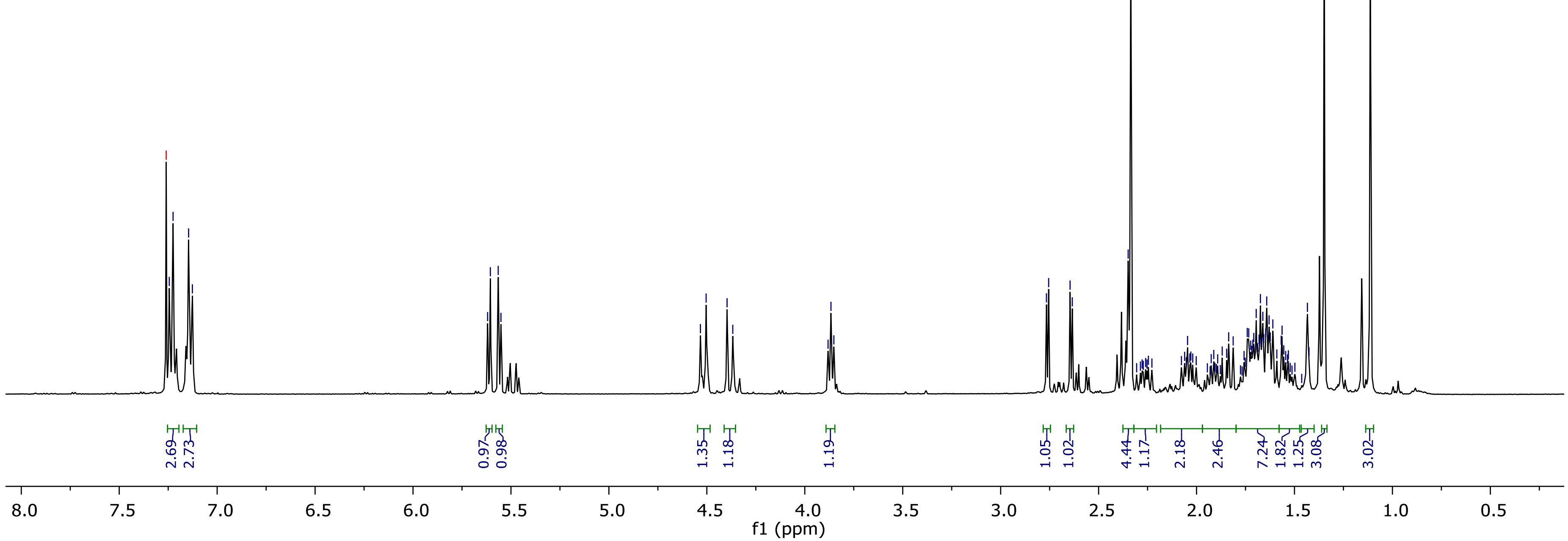

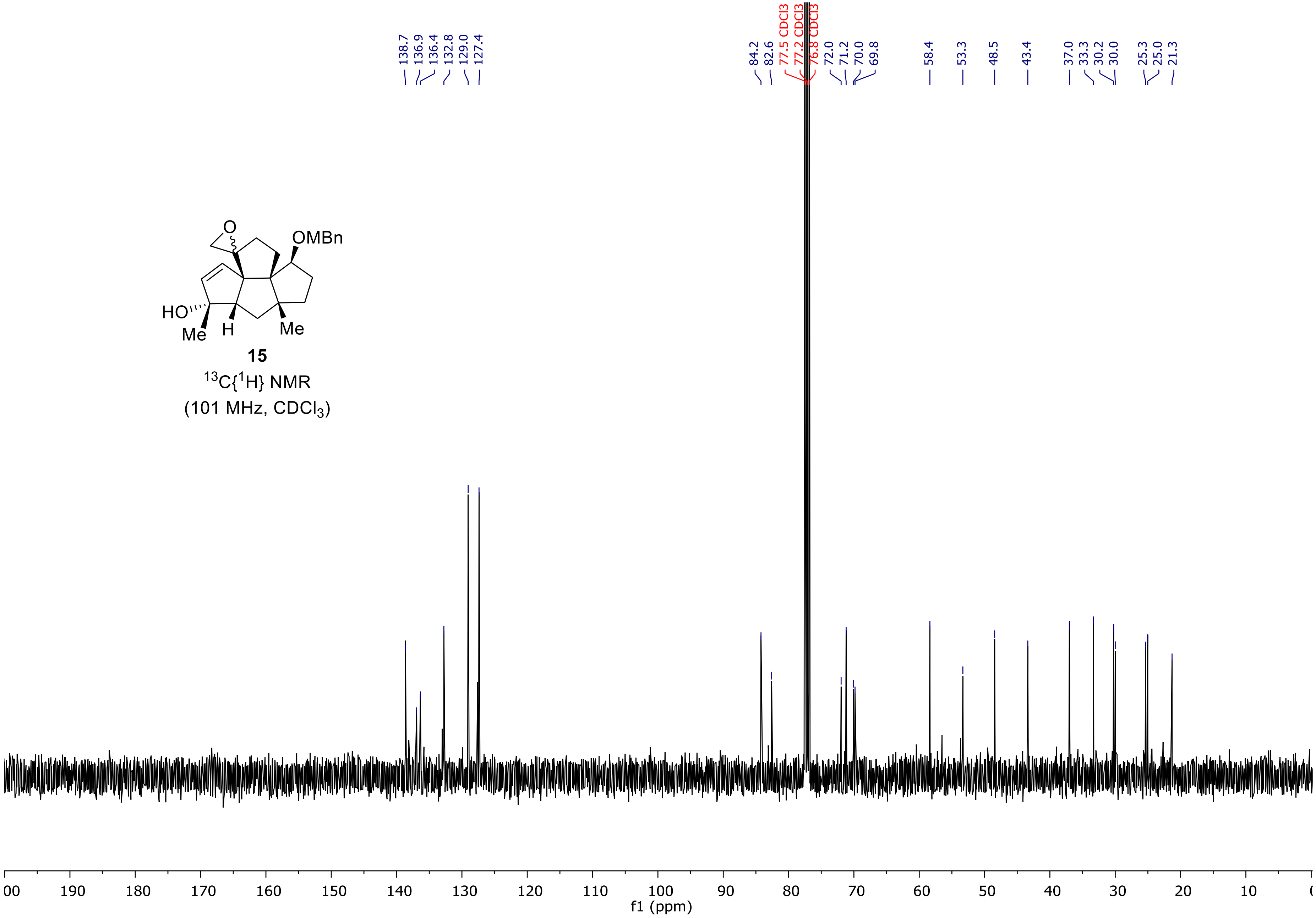

15

$^{13}C\{^1H\}$ NMR

(101 MHz, $CDCl_3$)

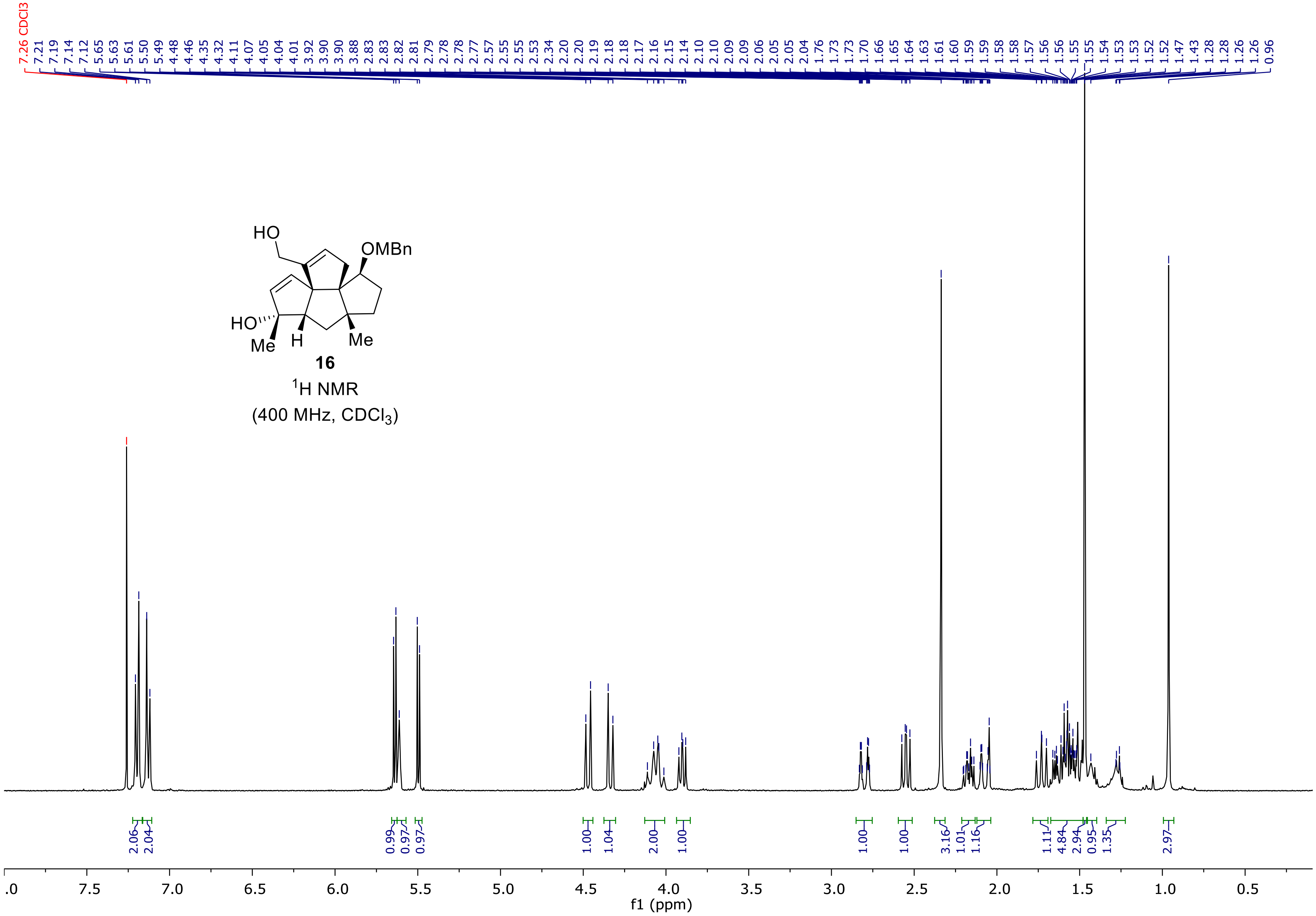
7.26 CDCl3
7.21 7.19 7.14 7.12 5.65 5.63 5.61 5.50 5.49 4.48 4.46 4.35 4.32 4.11 4.07 4.05 4.04 4.01 3.92 3.90 3.90 3.88 2.83 2.82 2.81 2.79 2.78 2.78 2.77 2.57 2.55 2.55 2.53 2.34 2.20 2.20 2.19 2.18 2.18 2.17 2.16 2.15 2.14 2.10 2.10 2.09 2.09 2.06 2.05 2.05 2.04 1.76 1.73 1.73 1.70 1.66 1.65 1.64 1.63 1.61 1.60 1.59 1.59 1.58 1.58 1.57 1.56 1.56 1.55 1.55 1.54 1.53 1.53 1.52 1.52 1.47 1.43 1.28 1.28 1.26 0.96
HO
OMBn
HO
Me
H
Me
16
1H NMR
(400 MHz, CDCl3)
2.06 2.04 0.99 0.97 0.97 1.00 1.04 2.00 1.00 1.00 1.00 3.16 1.01 1.16 1.11 4.84 2.94 0.95 1.35 2.97
.0 7.5 7.0 6.5 6.0 5.5 5.0 4.5 4.0 3.5 3.0 2.5 2.0 1.5 1.0 0.5
f1 (ppm)

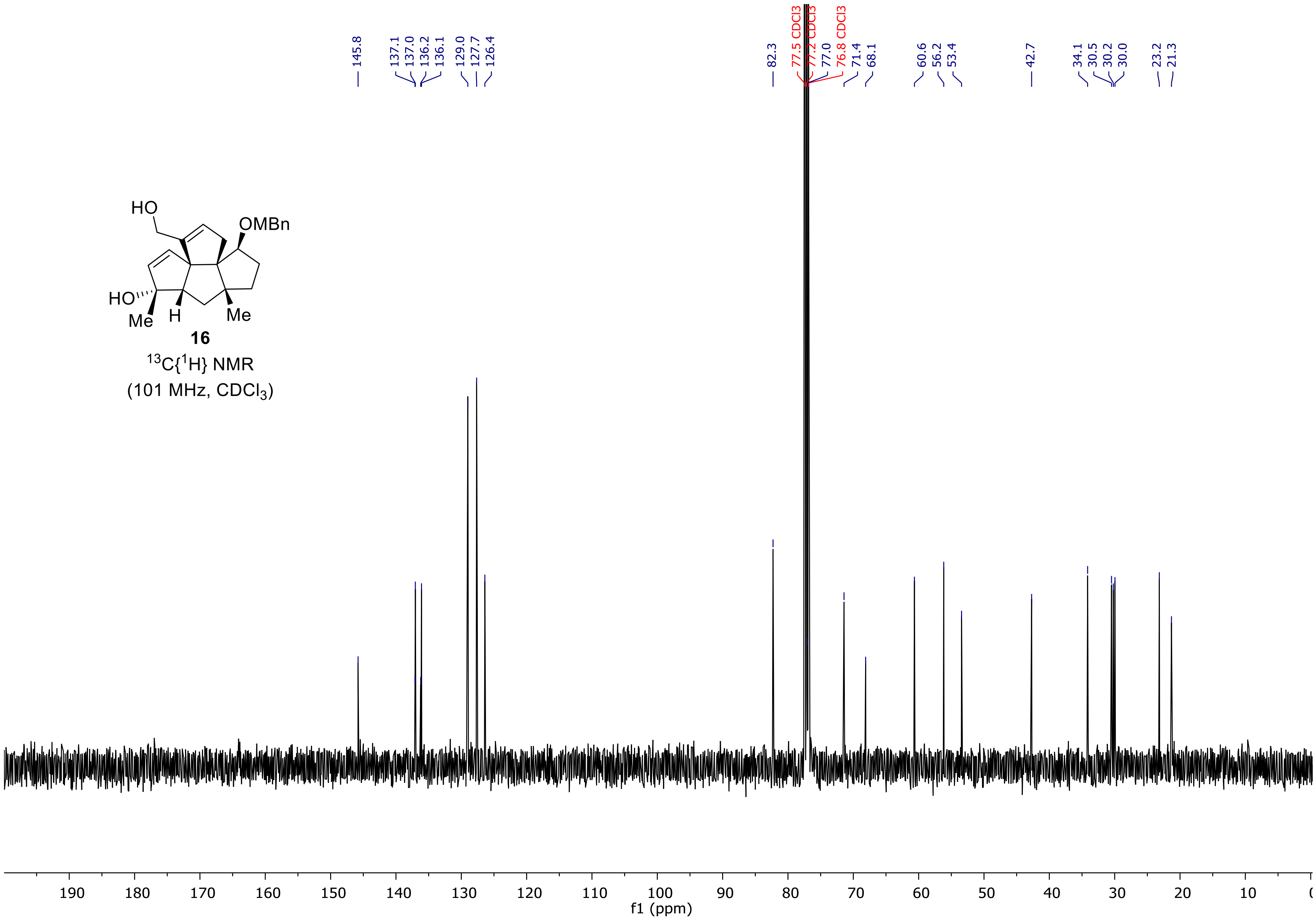

16

$^{13}C\{^{1}H\}$ NMR

(101 MHz, $CDCl_3$)

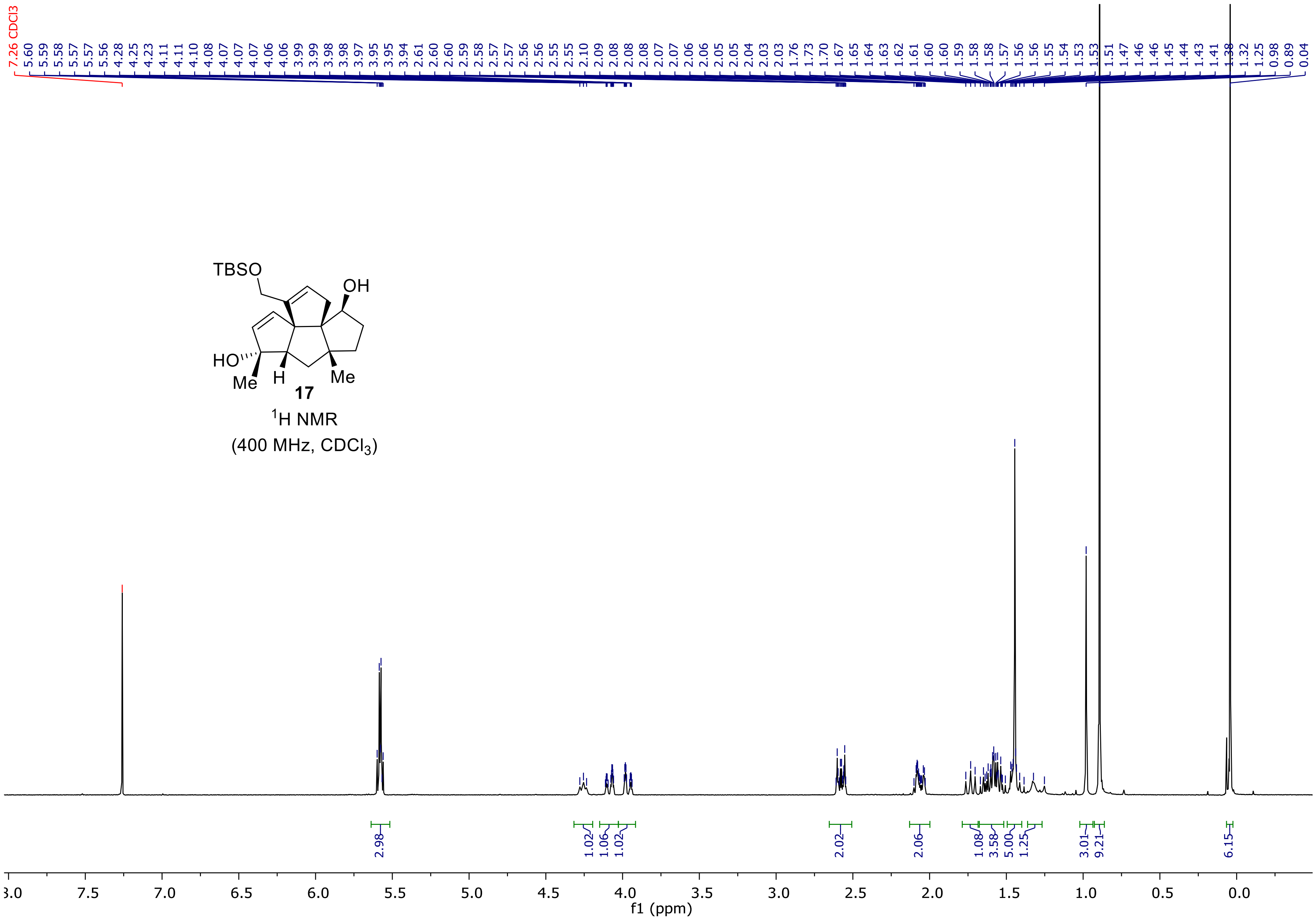

TBSO
OH
HO
Me
H
Me
17
1H NMR
(400 MHz, CDCl3)
7.26 CDCl3
f1 (ppm)

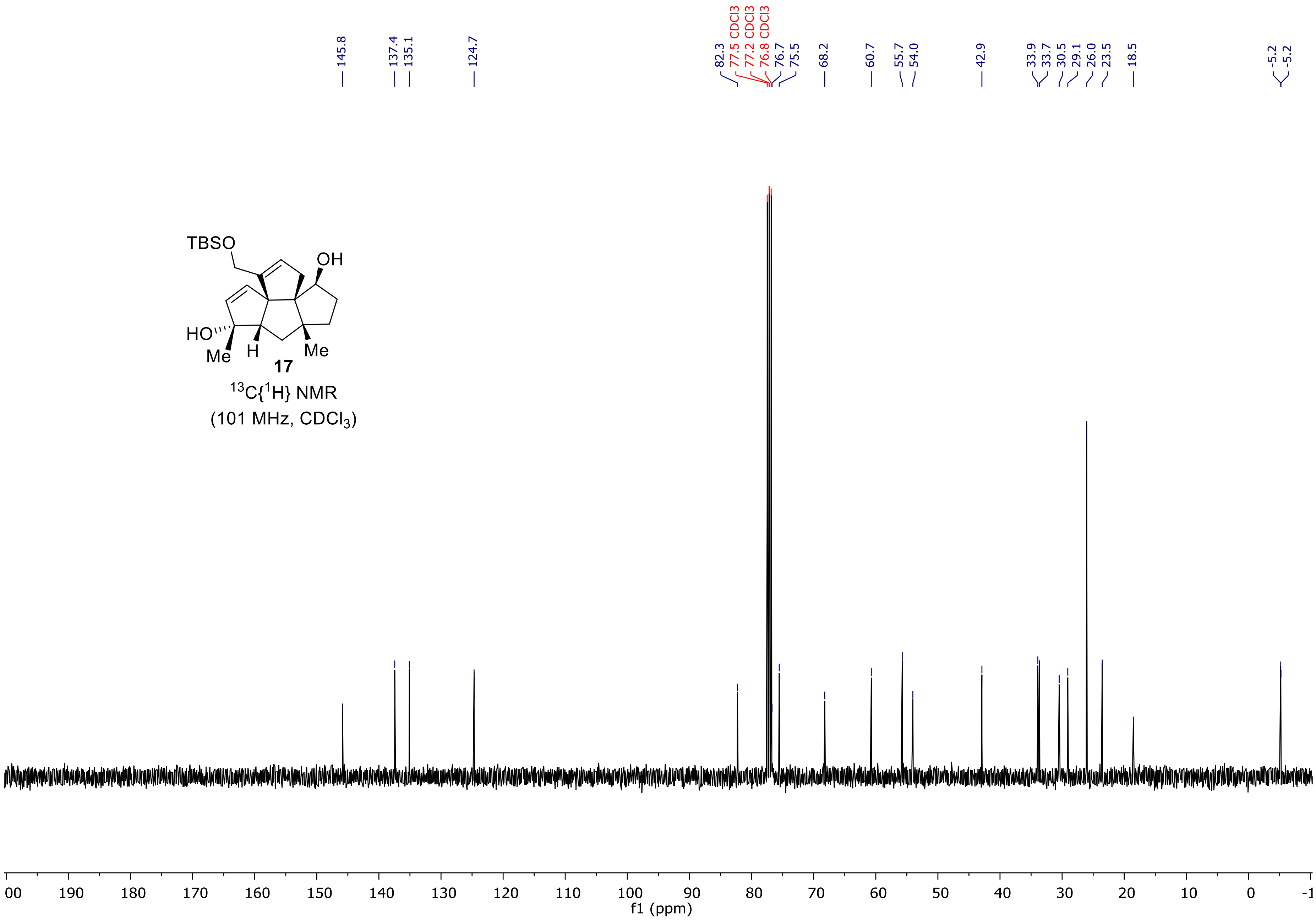
145.8
137.4
135.1
124.7
82.3
77.5 CDCl3
77.2 CDCl3
76.8 CDCl3
76.7
75.5
68.2
60.7
55.7
54.0
42.9
33.9
33.7
30.5
29.1
26.0
23.5
18.5
-5.2
-5.2
TBSO
OH
HO
Me
H
Me
17
13C{1H} NMR
(101 MHz, CDCl3)
f1 (ppm)

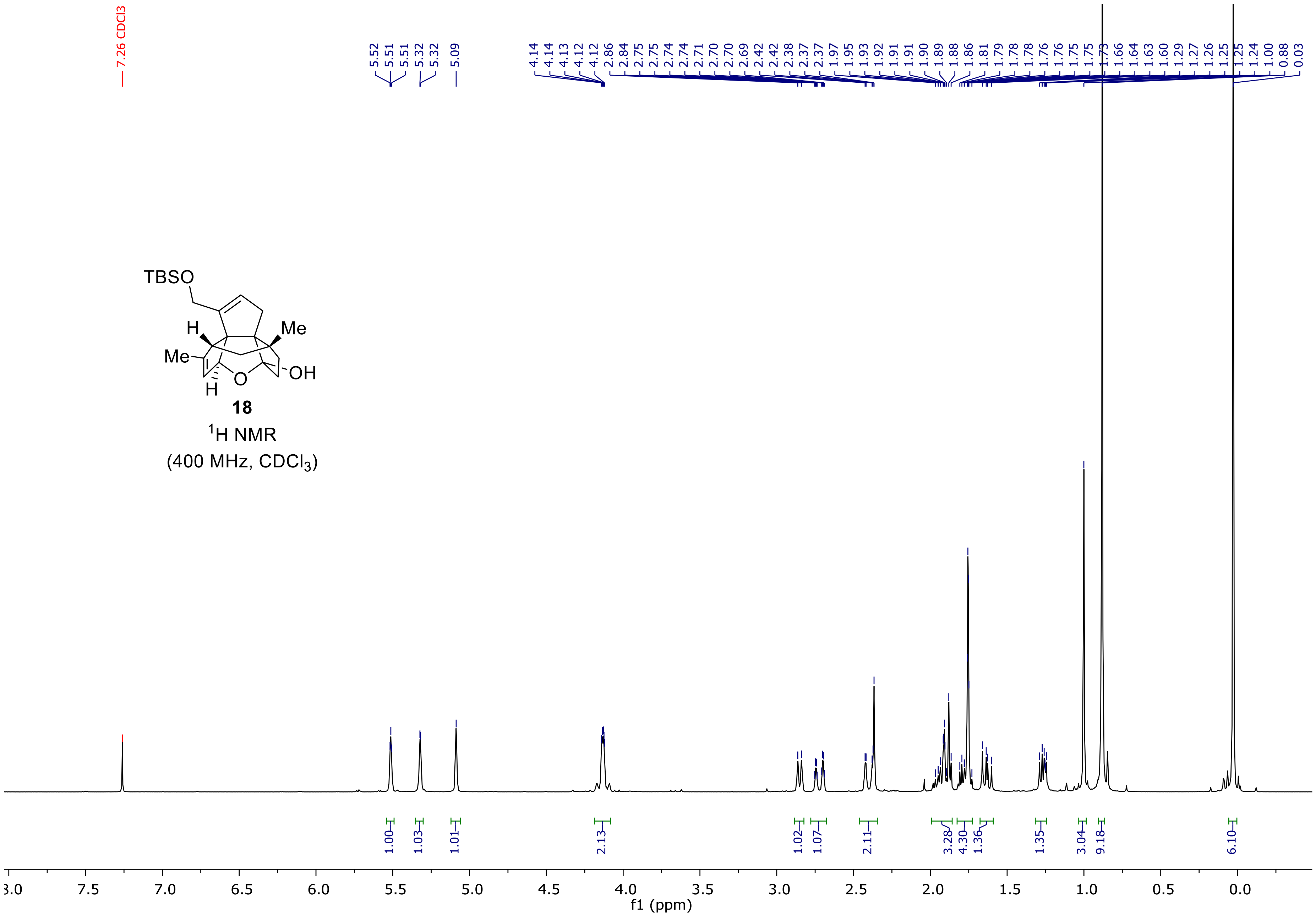

TBSO
H
Me
Me
OH
O
H
18
1H NMR
(400 MHz, CDCl3)
7.26 CDCl3
f1 (ppm)

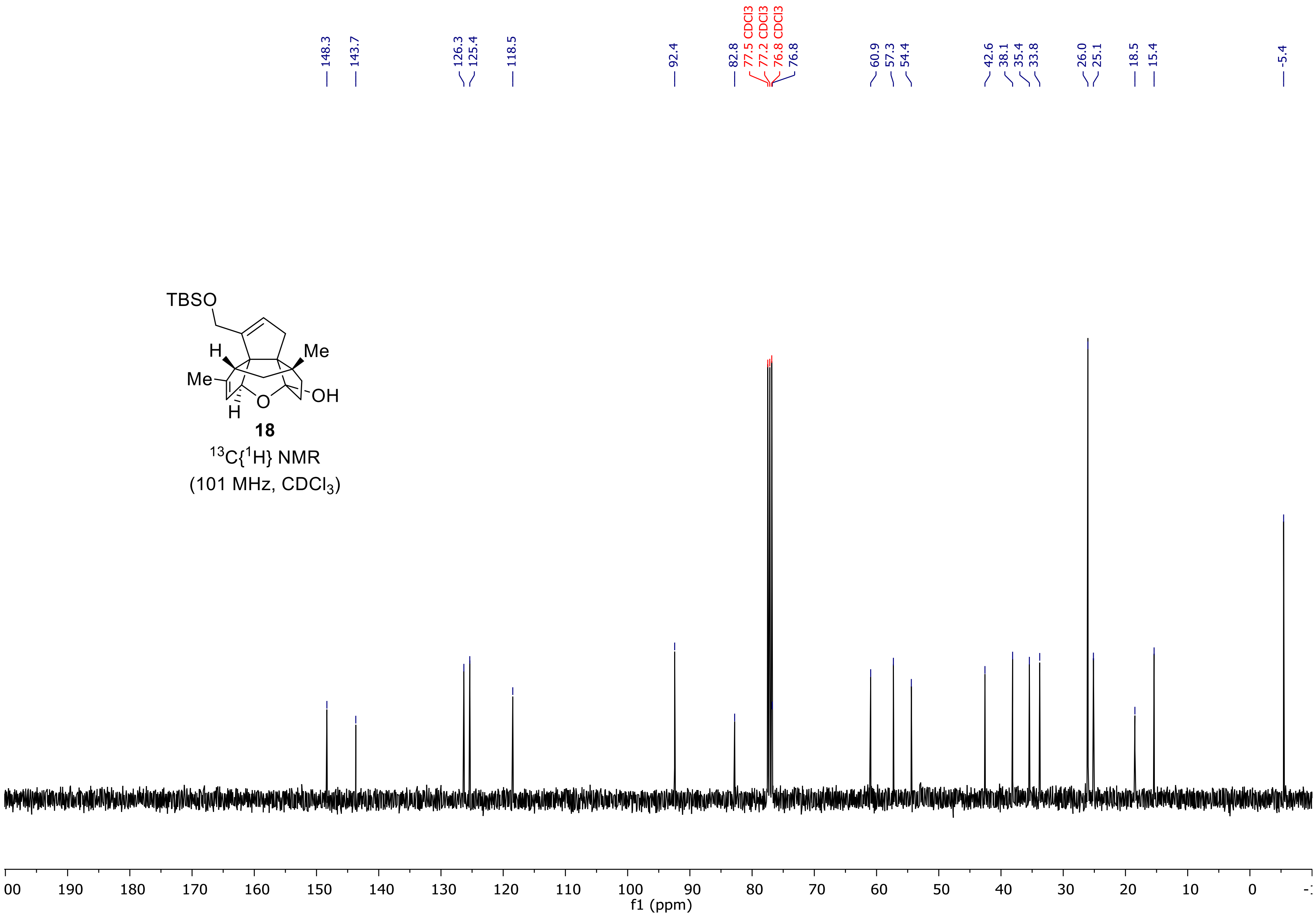
148.3
143.7
126.3
125.4
118.5
92.4
82.8
77.5 CDCl3
77.2 CDCl3
76.8 CDCl3
76.8
60.9
57.3
54.4
42.6
38.1
35.4
33.8
26.0
25.1
18.5
15.4
-5.4
TBSO
H
Me
Me
OH
H
O
18
13C{1H} NMR
(101 MHz, CDCl3)
f1 (ppm)

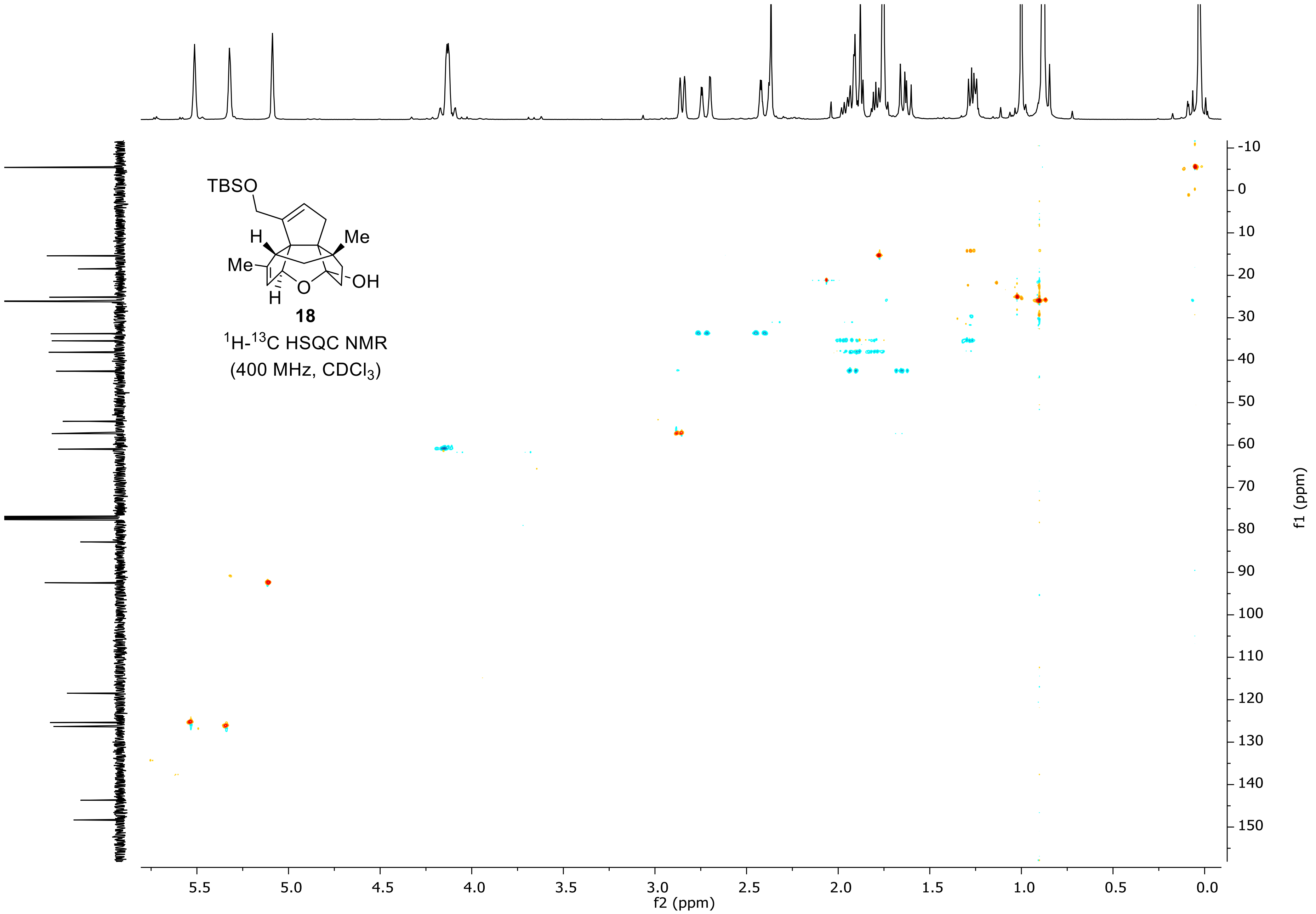
TBSO
H
Me
Me
OH
O
H
18
1H-13C HSQC NMR
(400 MHz, CDCl3)
f2 (ppm)
f1 (ppm)

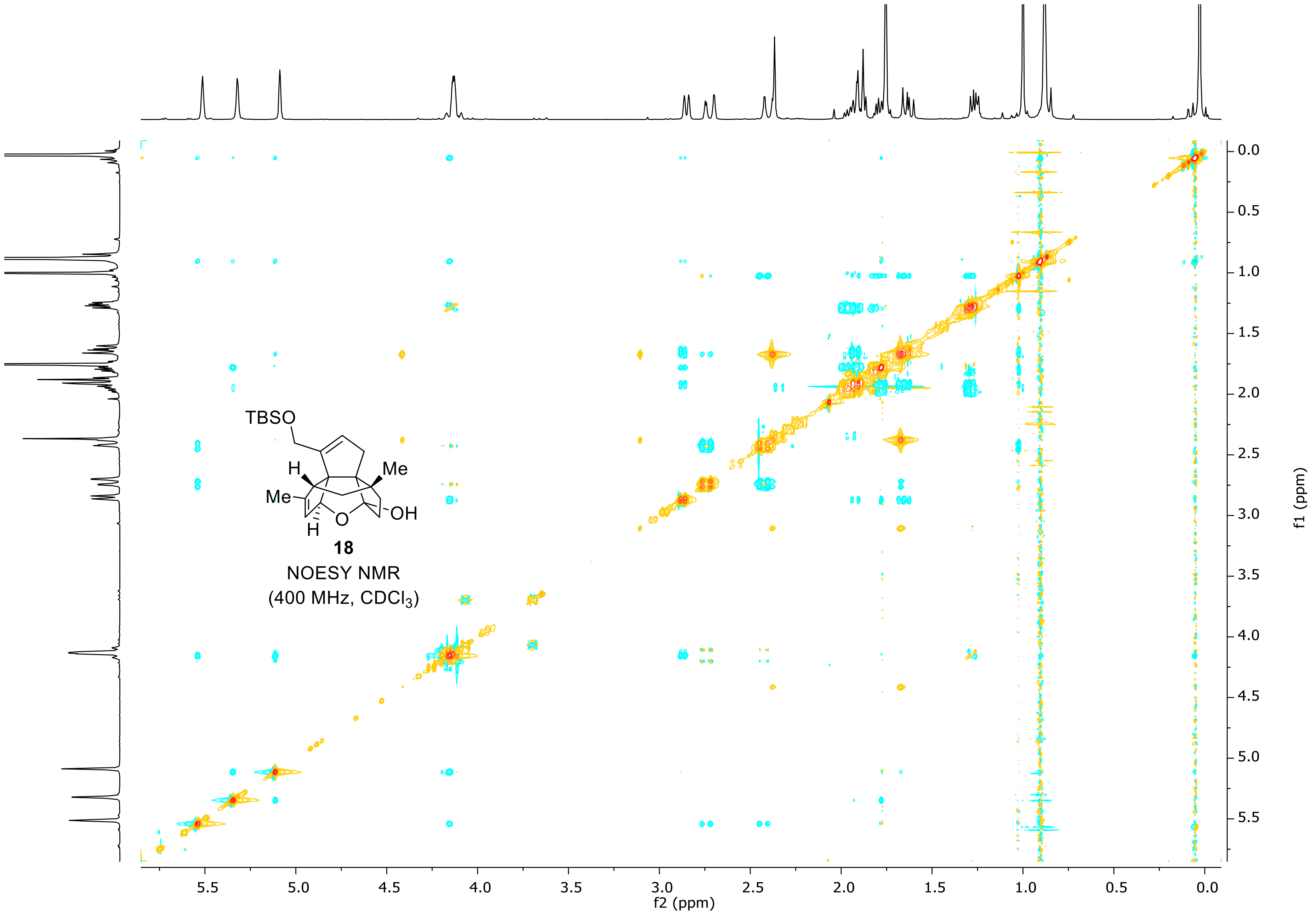
TBSO
H
Me
Me
OH
O
H
18
NOESY NMR
(400 MHz, $CDCl_3$)
f2 (ppm)
f1 (ppm)
5.5
5.0
4.5
4.0
3.5
3.0
2.5
2.0
1.5
1.0
0.5
0.0

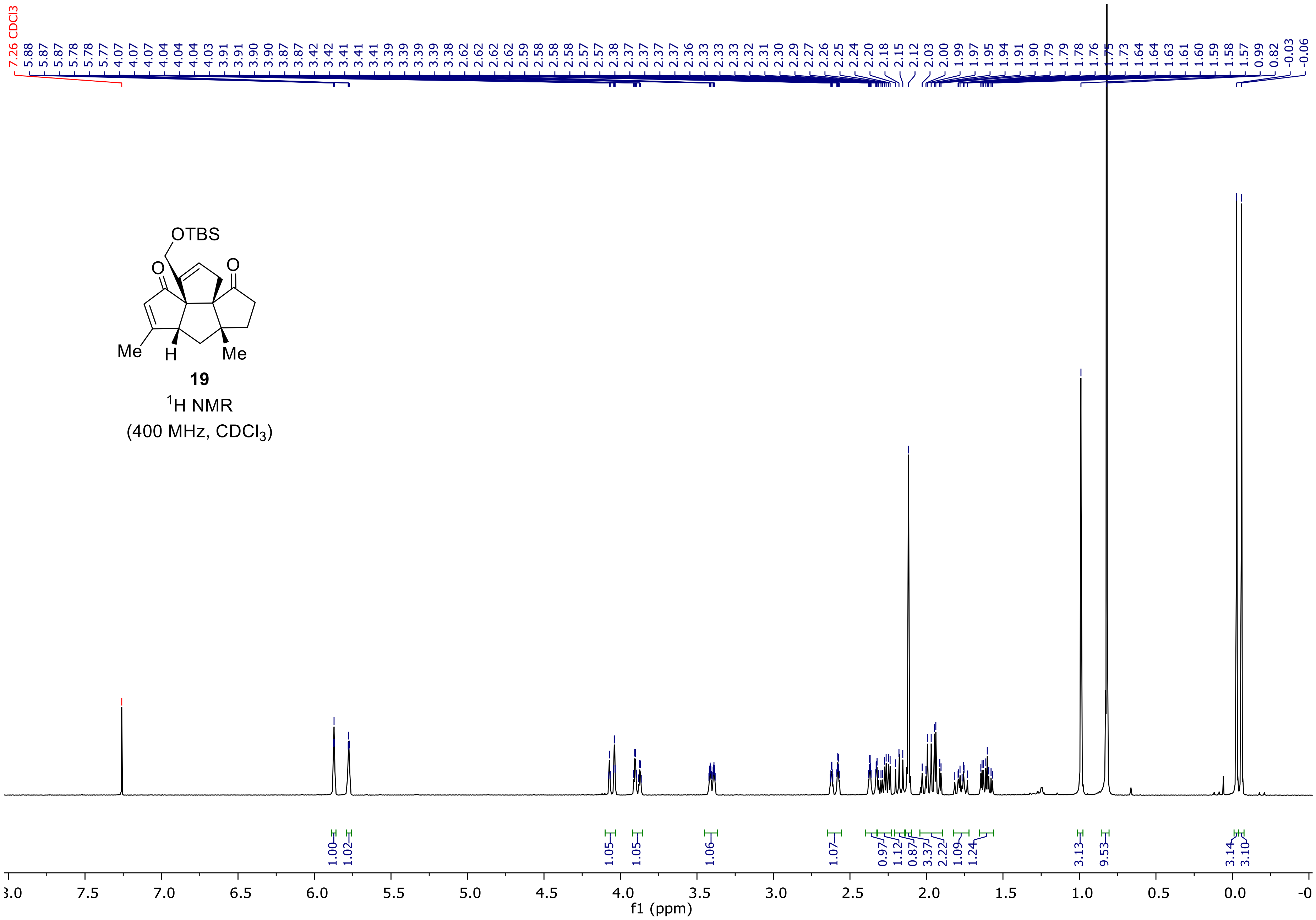
7.26 CDCl3
5.88 5.87 5.78 5.78 5.77 4.07 4.07 4.07 4.04 4.04 4.03 3.91 3.91 3.90 3.87 3.87 3.42 3.41 3.41 3.41 3.39 3.39 3.39 3.38 2.62 2.62 2.62 2.59 2.58 2.58 2.58 2.57 2.38 2.37 2.37 2.37 2.37 2.36 2.33 2.33 2.32 2.31 2.30 2.29 2.27 2.26 2.25 2.24 2.20 2.18 2.15 2.12 2.03 2.00 1.99 1.97 1.95 1.94 1.91 1.90 1.79 1.79 1.78 1.76 1.75 1.73 1.64 1.64 1.63 1.61 1.60 1.59 1.58 1.57 0.99 0.82 -0.03 -0.06
OTBS
O
O
Me
H
Me
19
^{1}H NMR
(400 MHz, $CDCl_3$)
1.00 1.02 1.05 1.05 1.06 1.07 0.97 1.12 0.87 3.37 2.22 1.09 1.24 3.13 9.53 3.14 3.10
7.5 7.0 6.5 6.0 5.5 5.0 4.5 4.0 3.5 3.0 2.5 2.0 1.5 1.0 0.5 0.0
f1 (ppm)

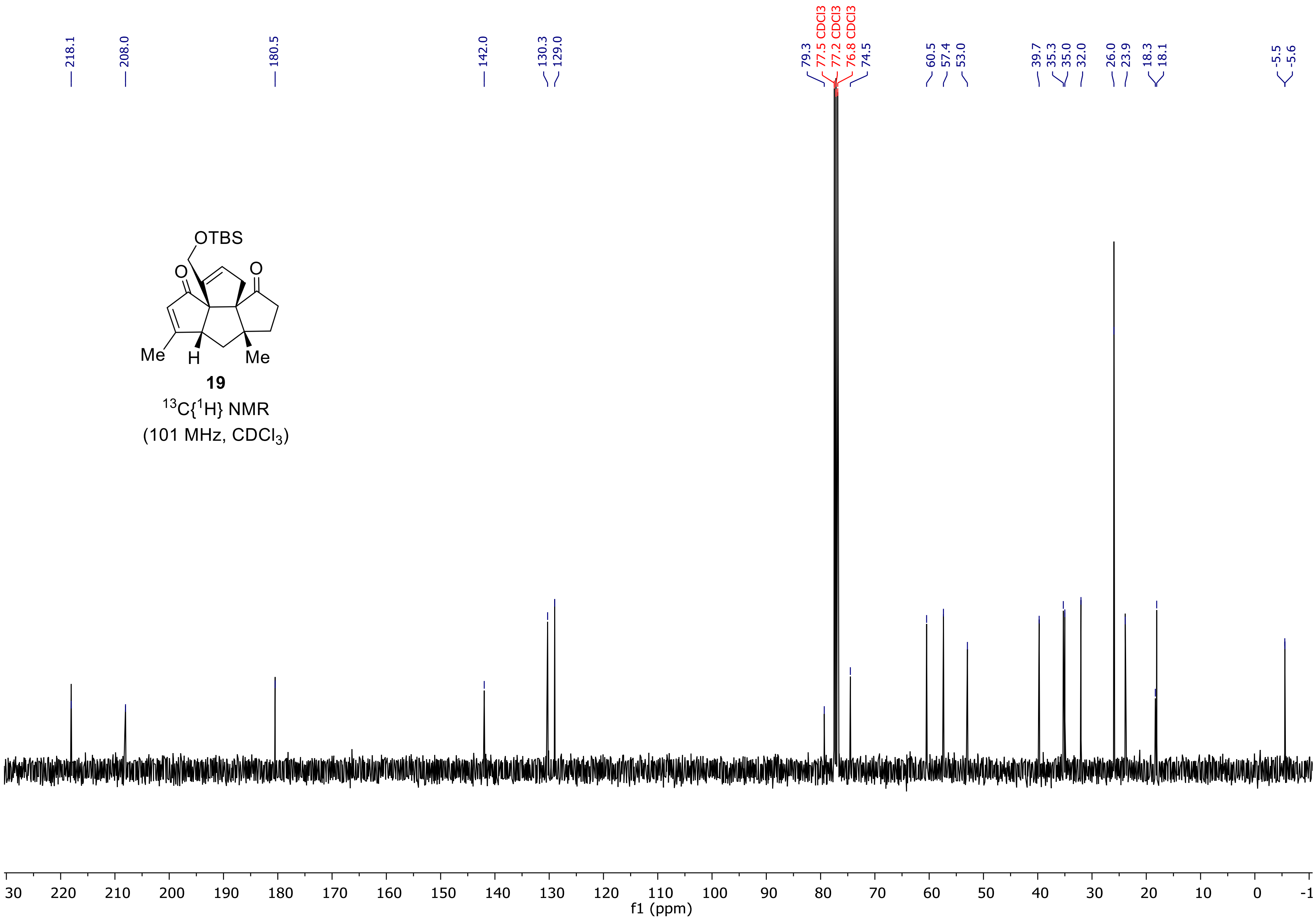

19

$^{13}C\{^1H\}$ NMR

(101 MHz, $CDCl_3$)

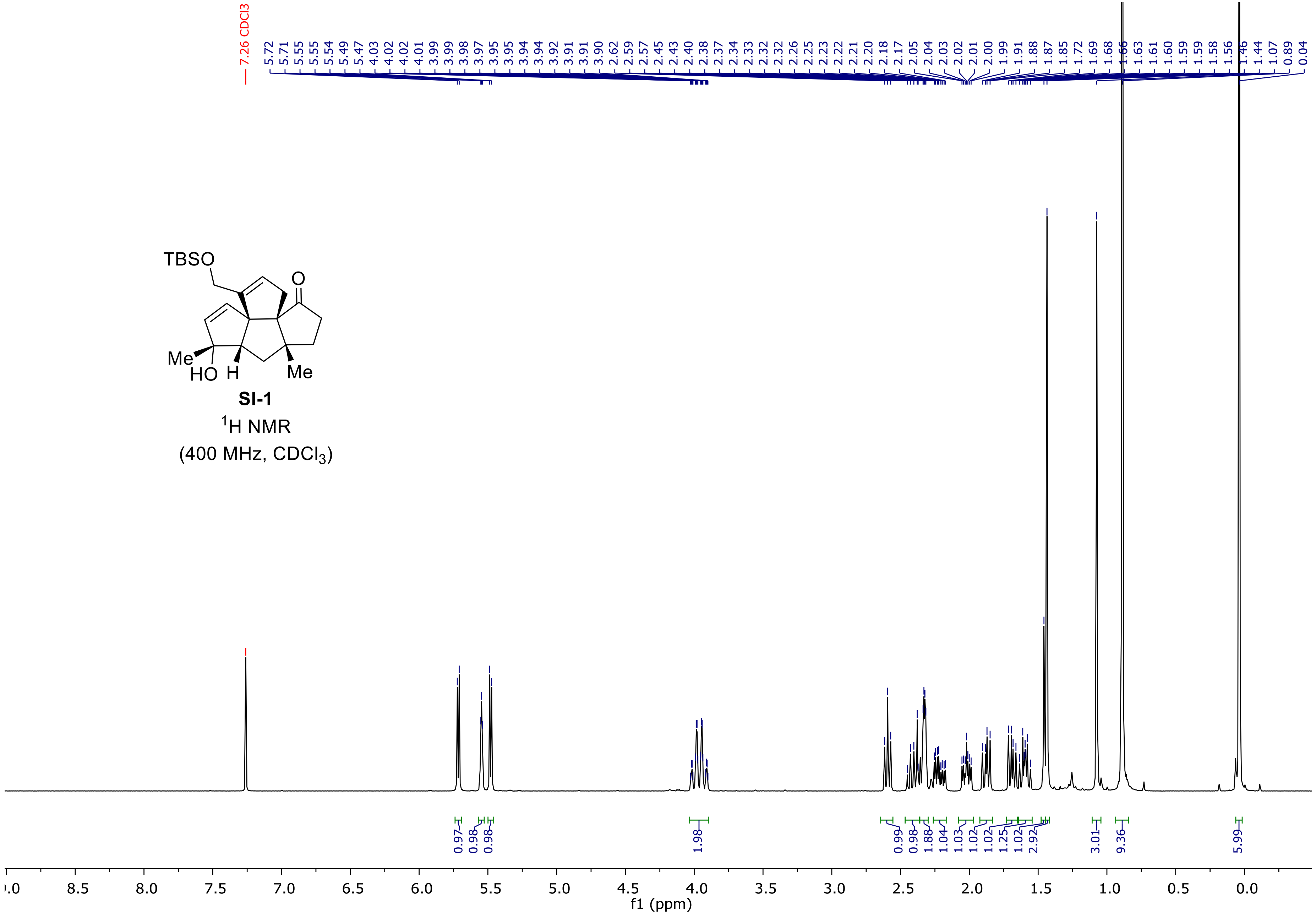

SI-1

^{1}H NMR

(400 MHz, $CDCl_3$)

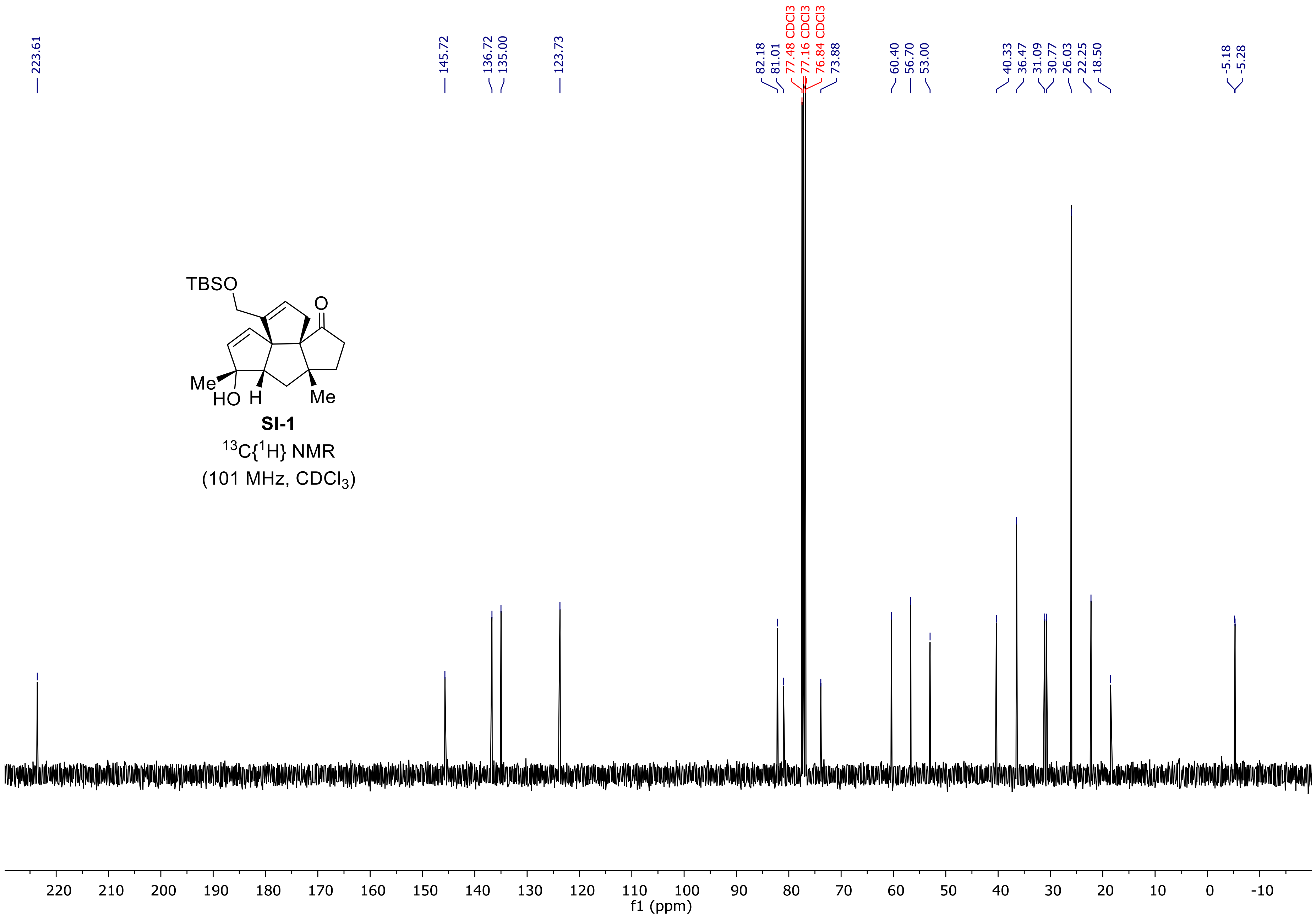

SI-1

$^{13}C\{^{1}H\}$ NMR

(101 MHz, $CDCl_3$)

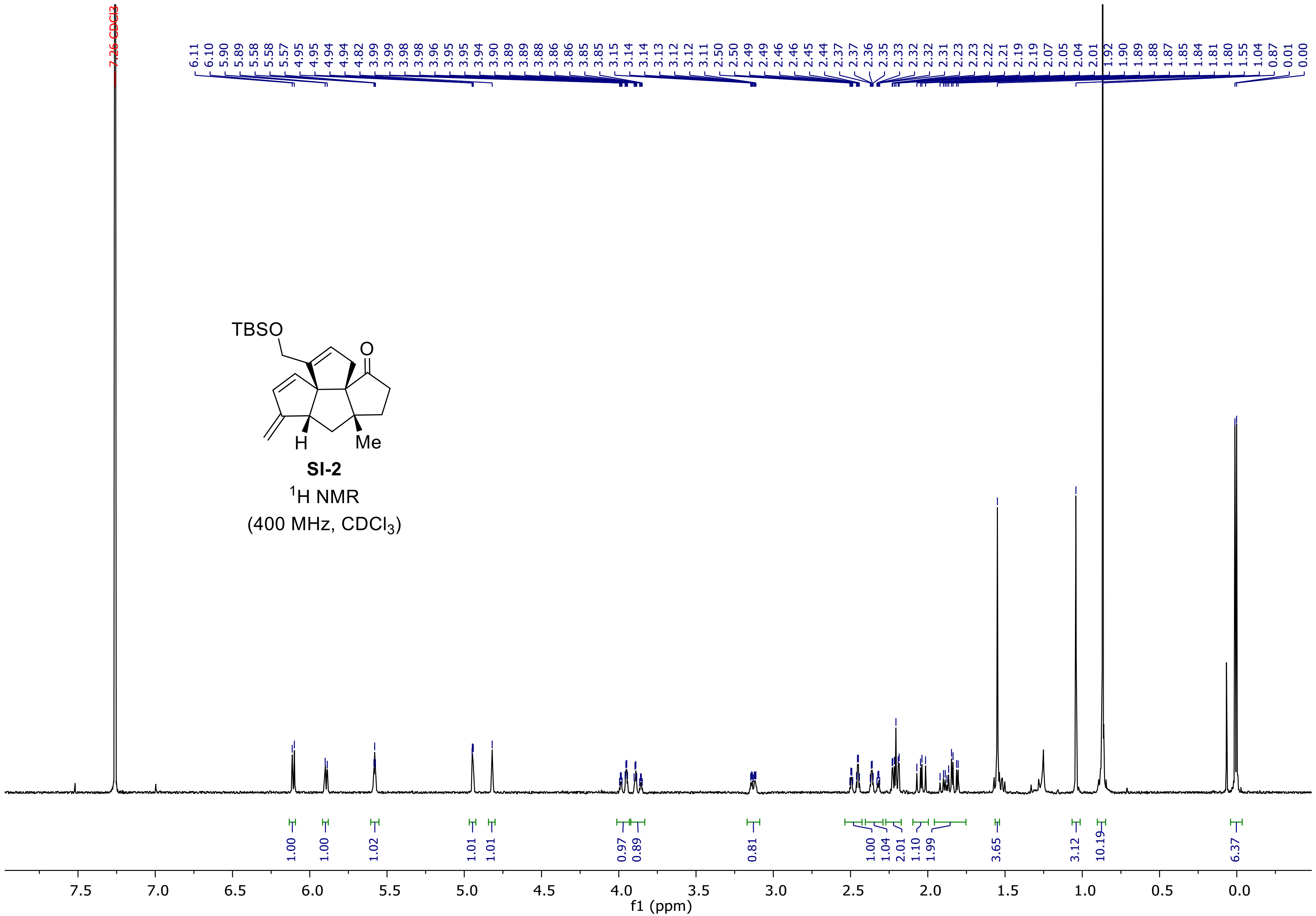
TBSO
O
H
Me
SI-2
^{1}H NMR
(400 MHz, $CDCl_3$)
f1 (ppm)

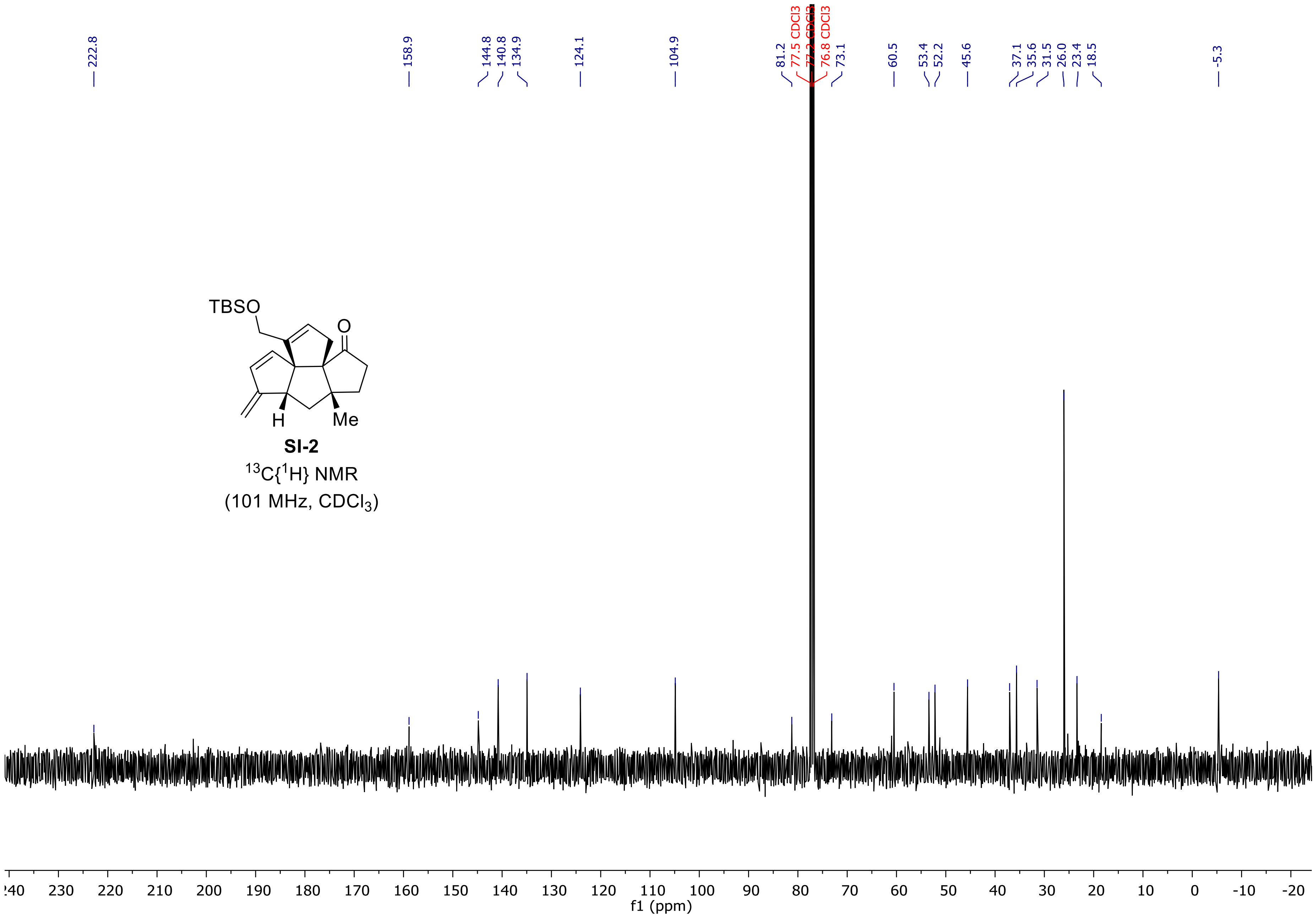

SI-2

$^{13}C\{^{1}H\}$ NMR

(101 MHz, $CDCl_3$)

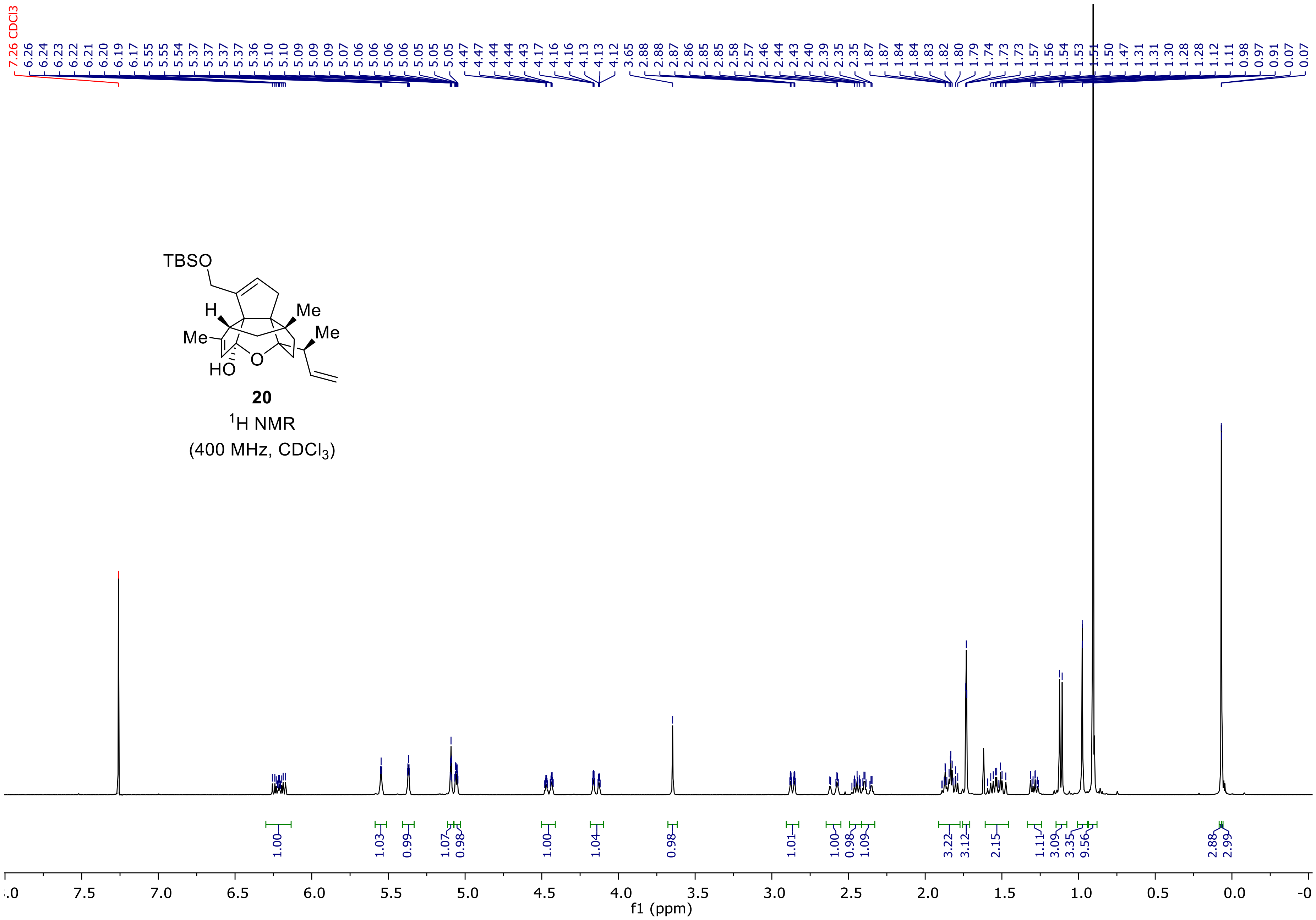
TBSO
H
Me
Me
Me
HO
O
20
1H NMR
(400 MHz, CDCl3)
7.26 CDCl3
f1 (ppm)

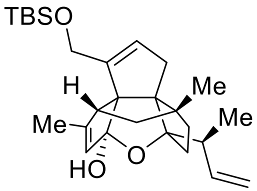

20

$^{13}C\{^{1}H\}$ NMR

(101 MHz, $CDCl_3$)

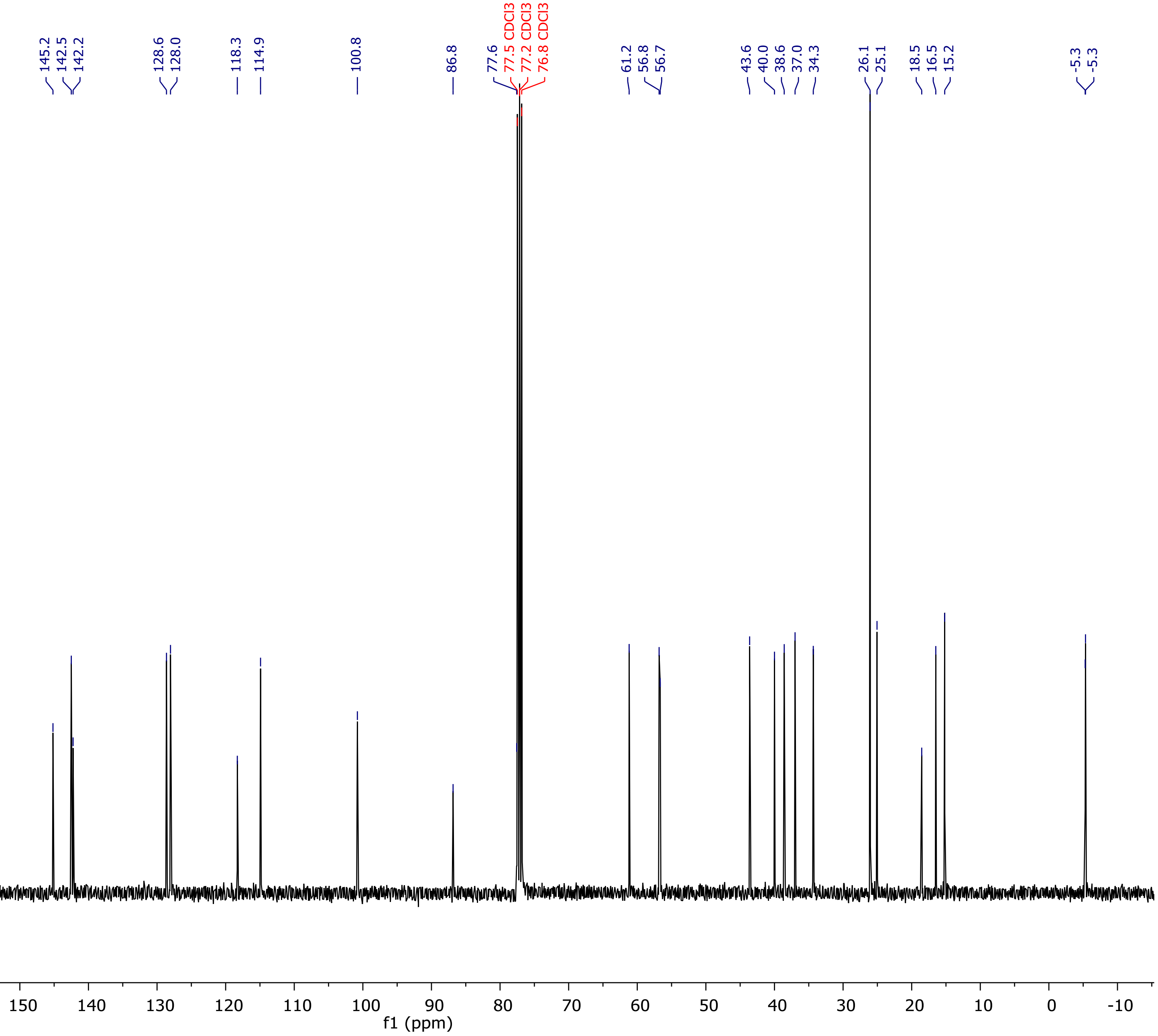

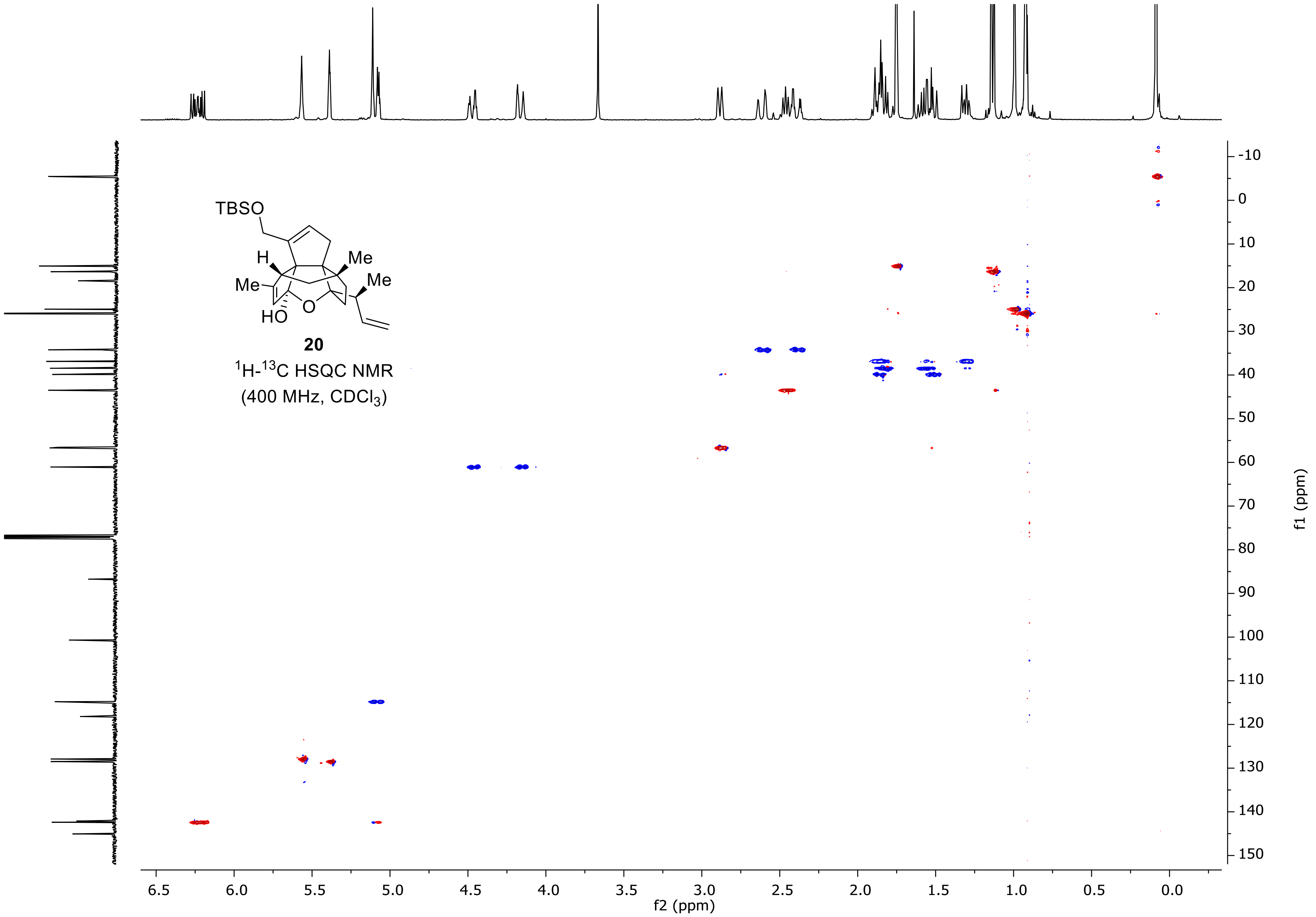

TBSO
H
Me
Me
Me
HO
O
20
^{1}H-^{13}C HSQC NMR
(400 MHz, CDCl$_3$)
f2 (ppm)
f1 (ppm)

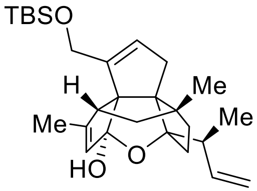

20

NOESY NMR

(400 MHz, $CDCl_3$)

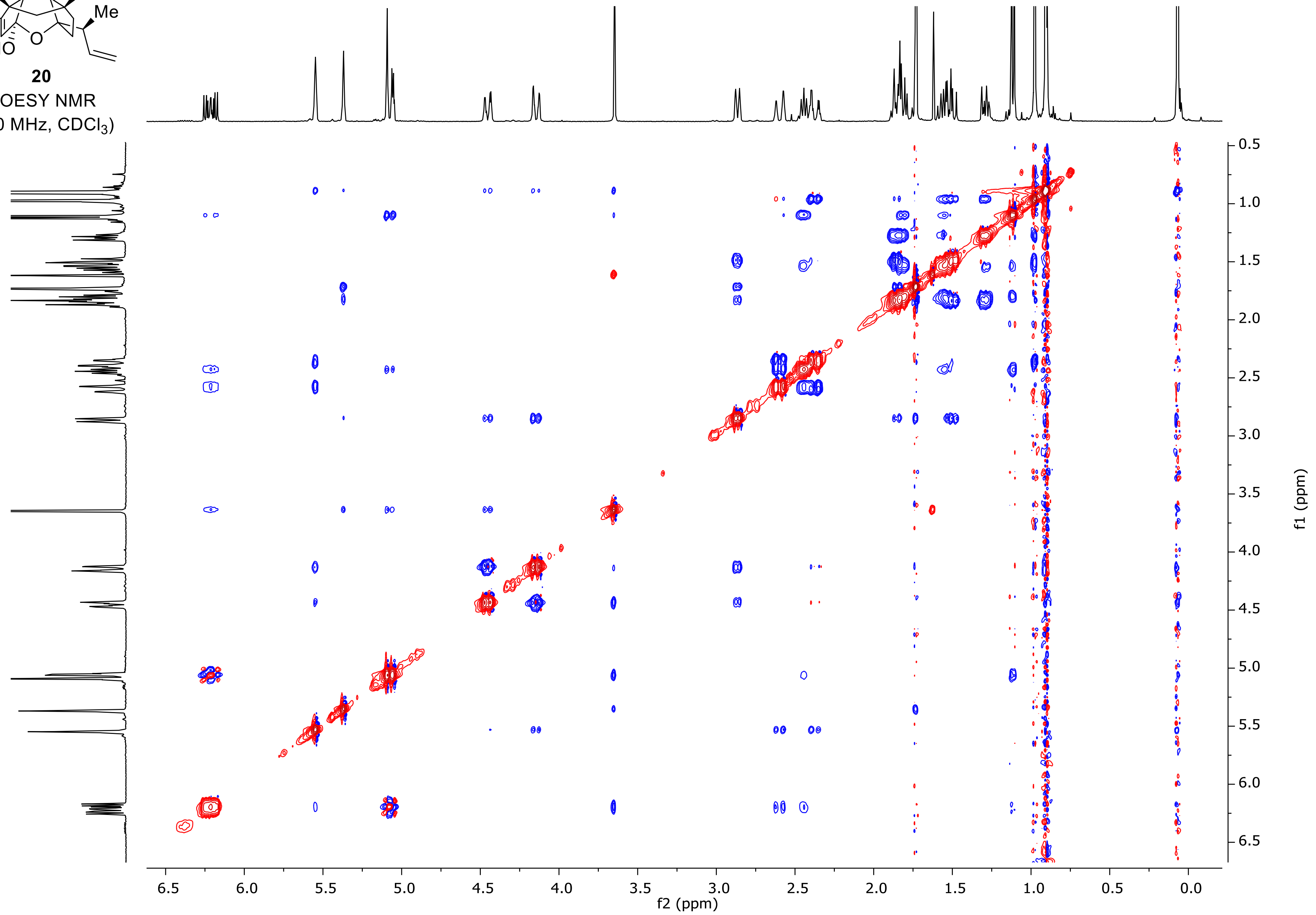

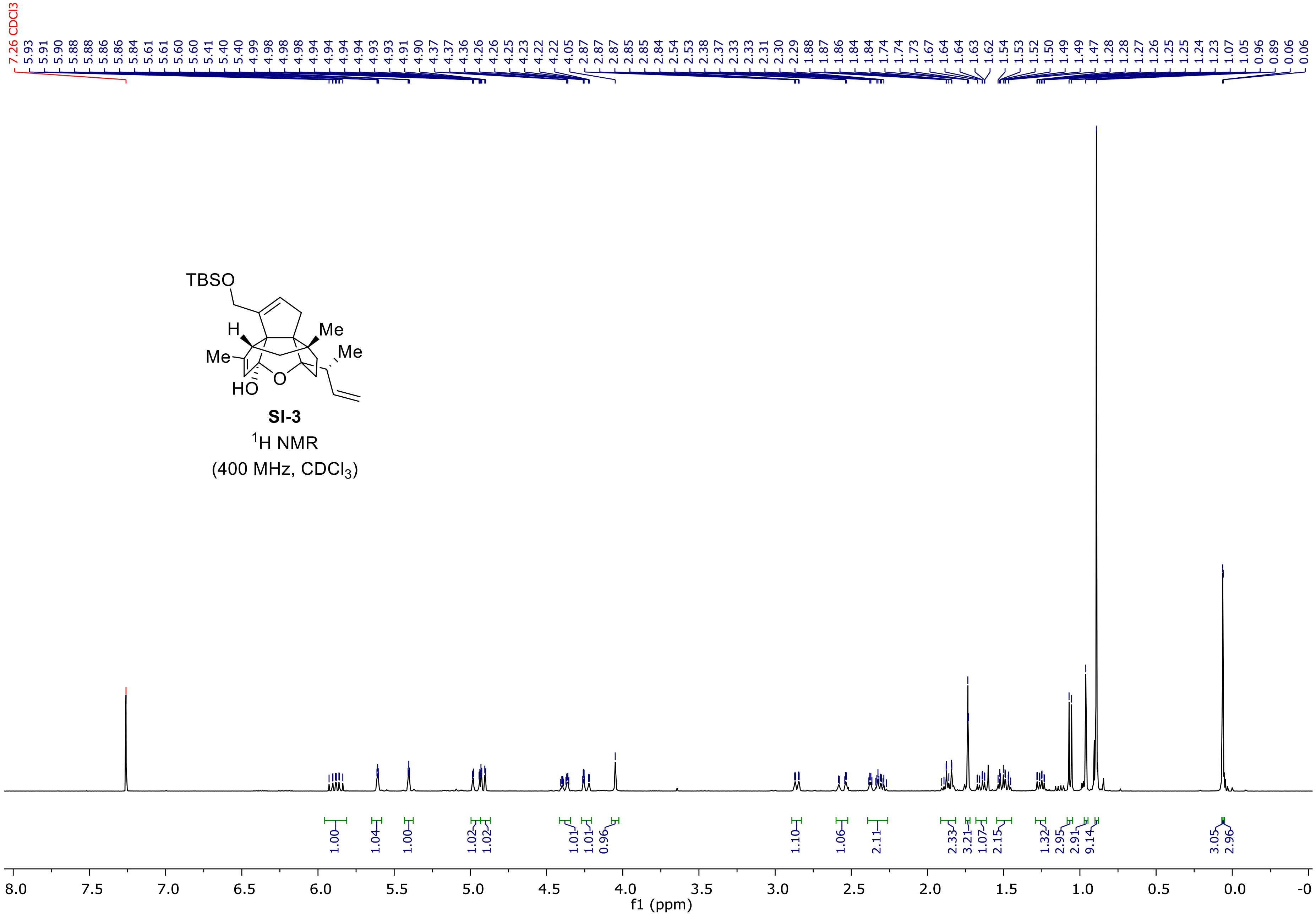
7.26 CDCl3
5.93 5.91 5.90 5.88 5.88 5.86 5.86 5.84 5.61 5.61 5.60 5.60 5.41 5.40 5.40 4.99 4.98 4.98 4.98 4.94 4.94 4.94 4.94 4.93 4.93 4.91 4.90 4.37 4.37 4.36 4.26 4.26 4.25 4.23 4.22 4.22 4.05 2.87 2.87 2.87 2.85 2.85 2.84 2.54 2.53 2.38 2.37 2.33 2.33 2.31 2.30 2.29 1.88 1.87 1.86 1.84 1.84 1.74 1.74 1.73 1.67 1.64 1.64 1.63 1.62 1.54 1.53 1.52 1.50 1.49 1.49 1.47 1.28 1.28 1.27 1.26 1.25 1.25 1.24 1.23 1.07 1.05 0.96 0.89 0.06 0.06
TBSO
H
Me
Me
Me
HO
O
SI-3
^{1}H NMR
(400 MHz, CDCl$_3$)
1.00 1.04 1.00 1.02 1.02 1.01 1.01 0.96 1.10 1.06 2.11 2.33 3.21 1.07 2.15 1.32 2.95 2.91 9.14 3.05 2.96
8.0 7.5 7.0 6.5 6.0 5.5 5.0 4.5 4.0 3.5 3.0 2.5 2.0 1.5 1.0 0.5 0.0 -0
f1 (ppm)

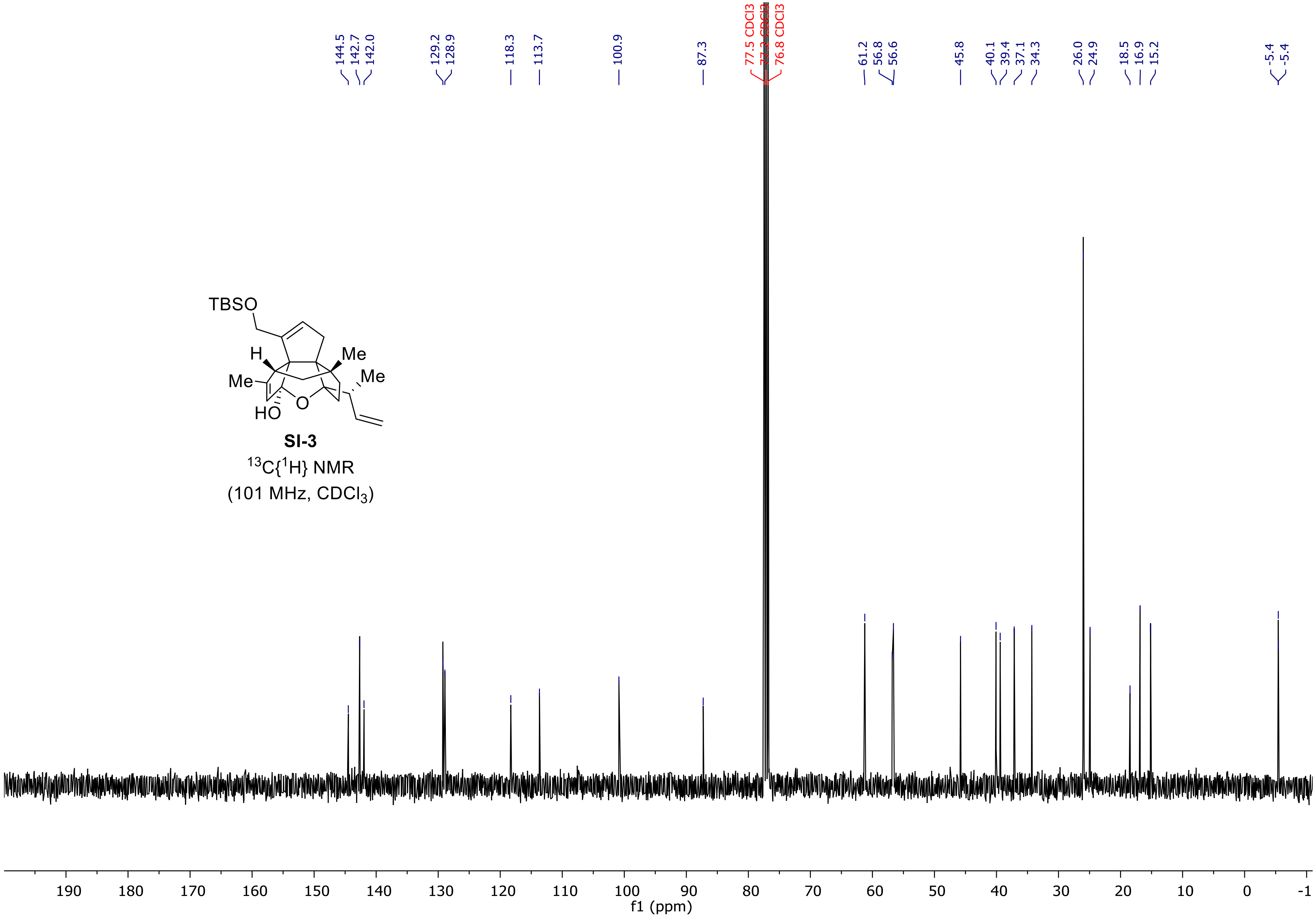

SI-3

$^{13}C\{^{1}H\}$ NMR

(101 MHz, $CDCl_3$)

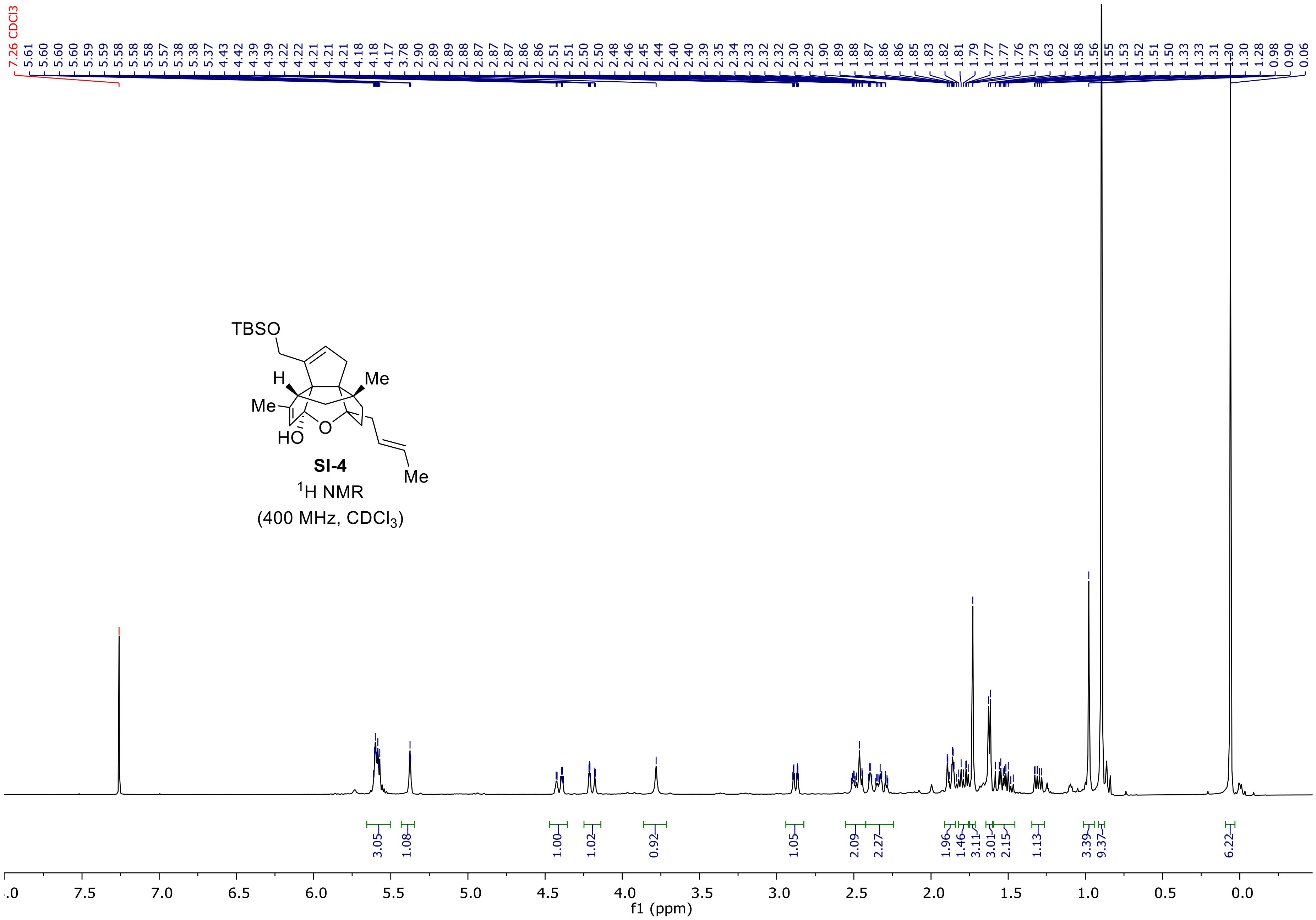
TBSO
H
Me
Me
HO
O
Me
SI-4
^{1}H NMR
(400 MHz, CDCl$_3$)
f1 (ppm)

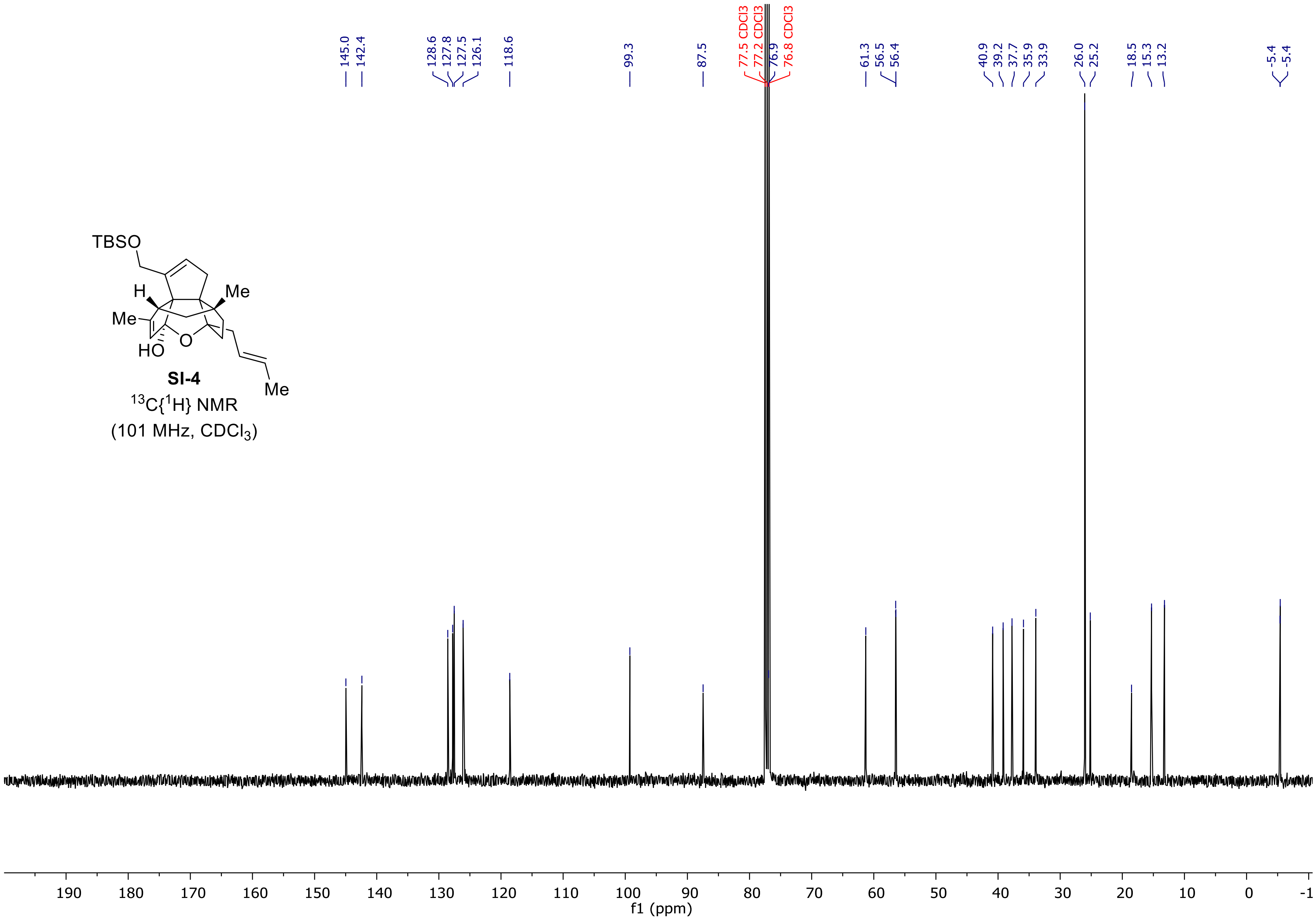
TBSO
H
Me
Me
HO
O
Me
SI-4
$^{13}C\{^1H\}$ NMR
(101 MHz, $CDCl_3$)
145.0
142.4
128.6
127.8
127.5
126.1
118.6
99.3
87.5
77.5 CDCl3
77.2 CDCl3
76.9
76.8 CDCl3
61.3
56.5
56.4
40.9
39.2
37.7
35.9
33.9
26.0
25.2
18.5
15.3
13.2
-5.4
-5.4
f1 (ppm)

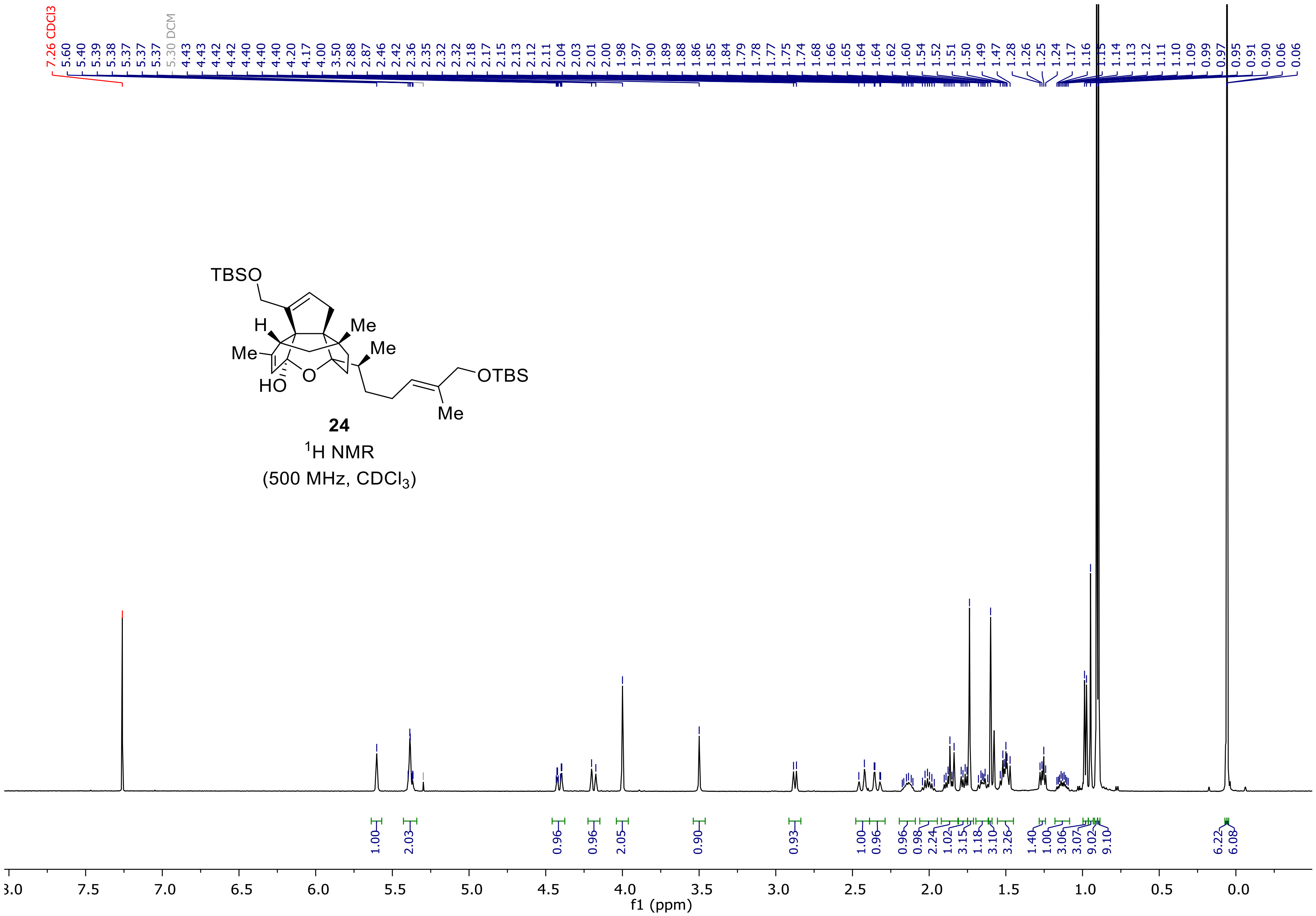
7.26 CDCl3
5.30 DCM
TBSO
H
Me
Me
Me
HO
O
OTBS
Me
24
1H NMR
(500 MHz, CDCl3)
f1 (ppm)

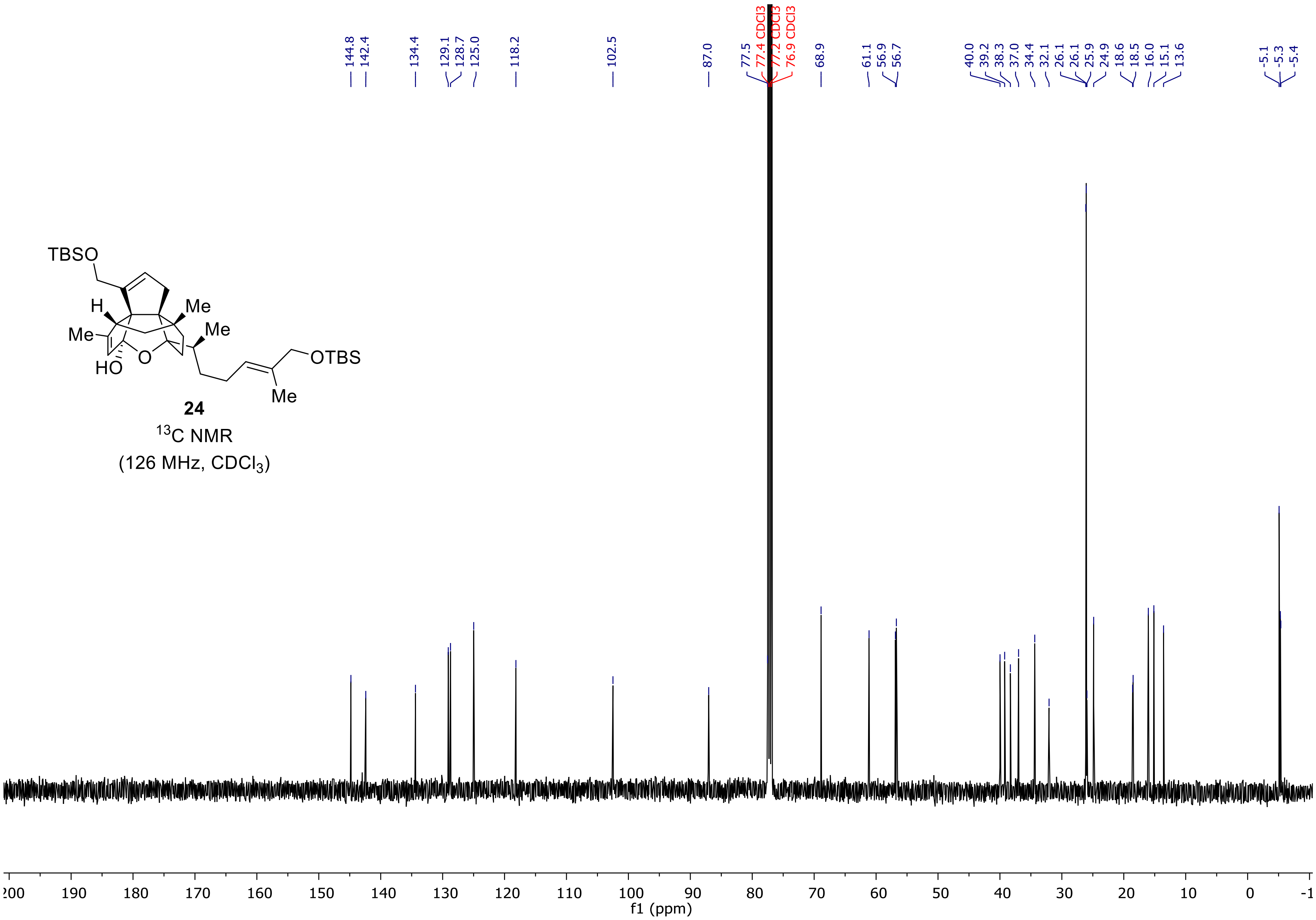

24

^{13}C NMR

(126 MHz, $CDCl_3$)

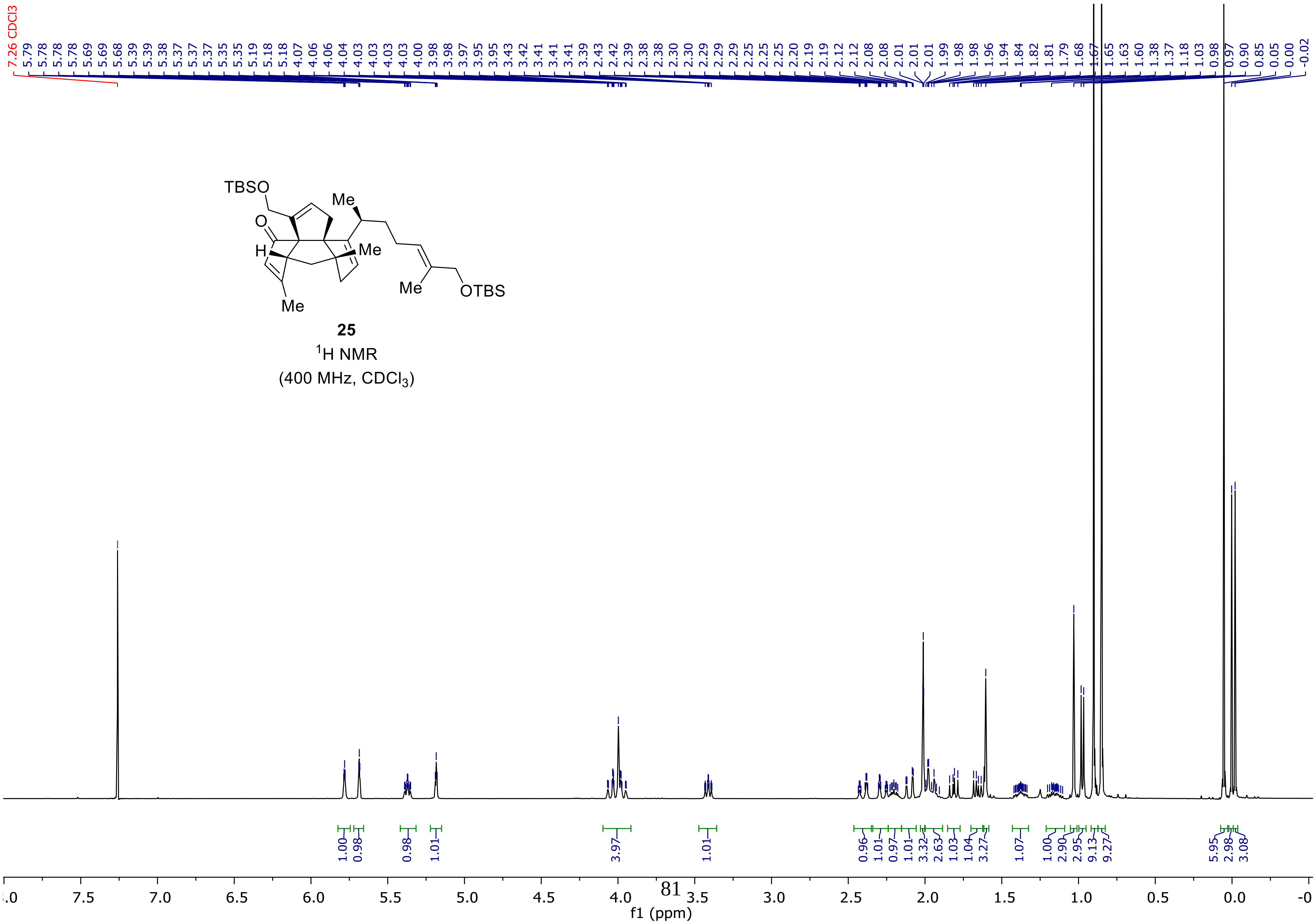

25

^{1}H NMR

(400 MHz, $CDCl_3$)

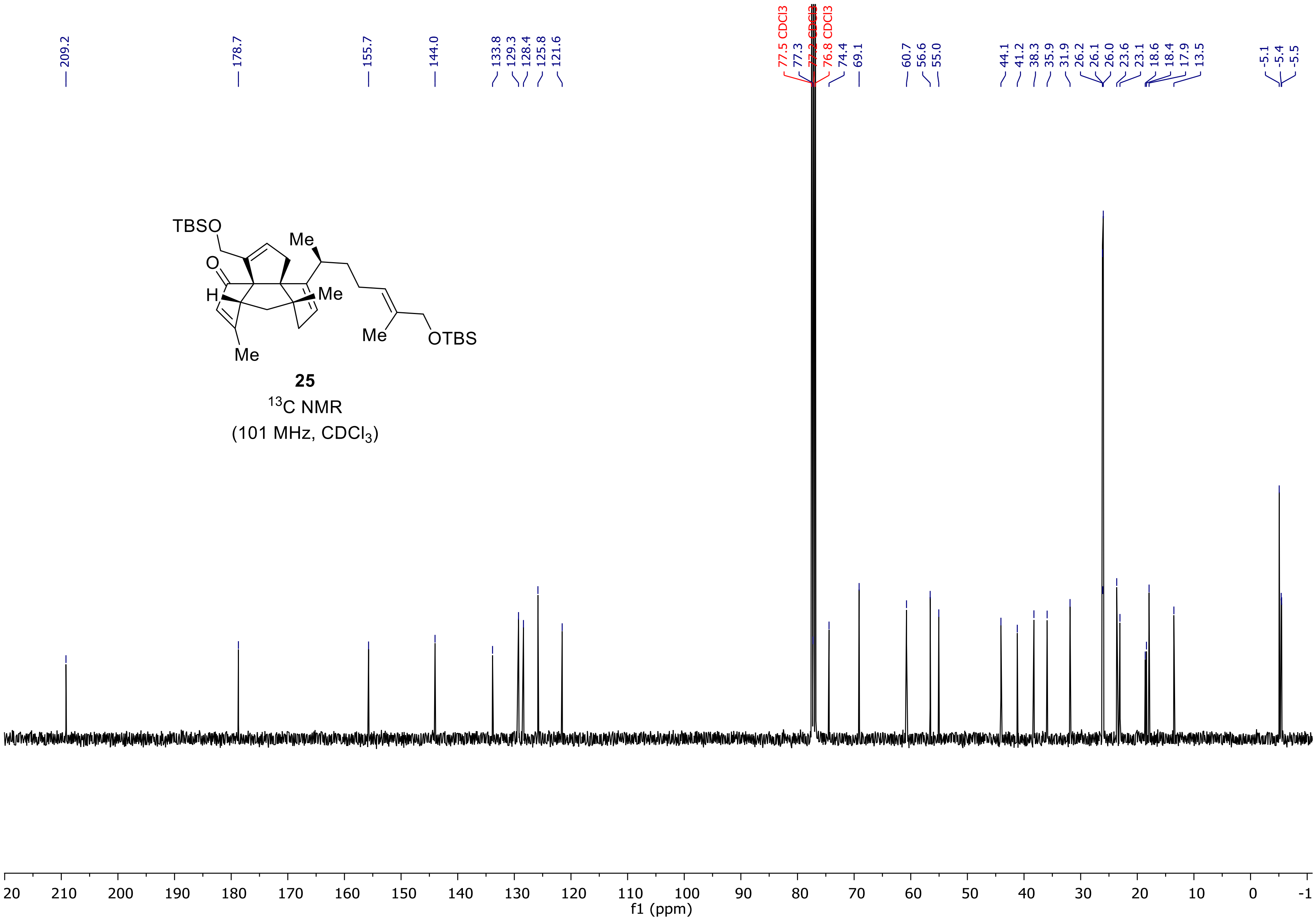

209.2
178.7
155.7
144.0
133.8
129.3
128.4
125.8
121.6
77.5 CDCl3
77.3
77.0 CDCl3
76.8 CDCl3
74.4
69.1
60.7
56.6
55.0
44.1
41.2
38.3
35.9
31.9
26.2
26.1
26.0
23.6
23.1
18.6
18.4
17.9
13.5
-5.1
-5.4
-5.5
TBSO
O
H
Me
Me
Me
Me
OTBS
25
^{13}C NMR
(101 MHz, $CDCl_3$)
f1 (ppm)

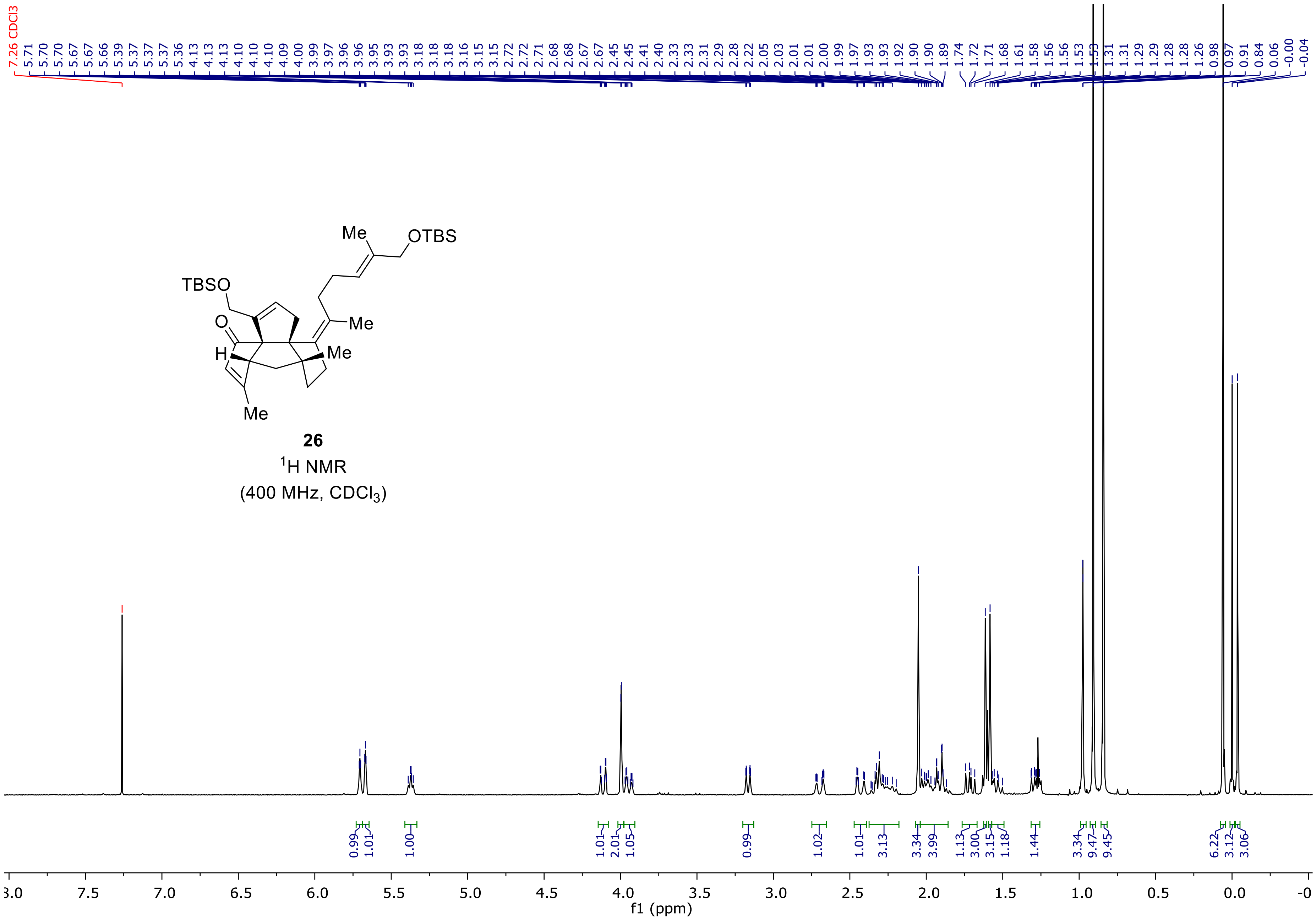

26

^{1}H NMR

(400 MHz, $CDCl_3$)

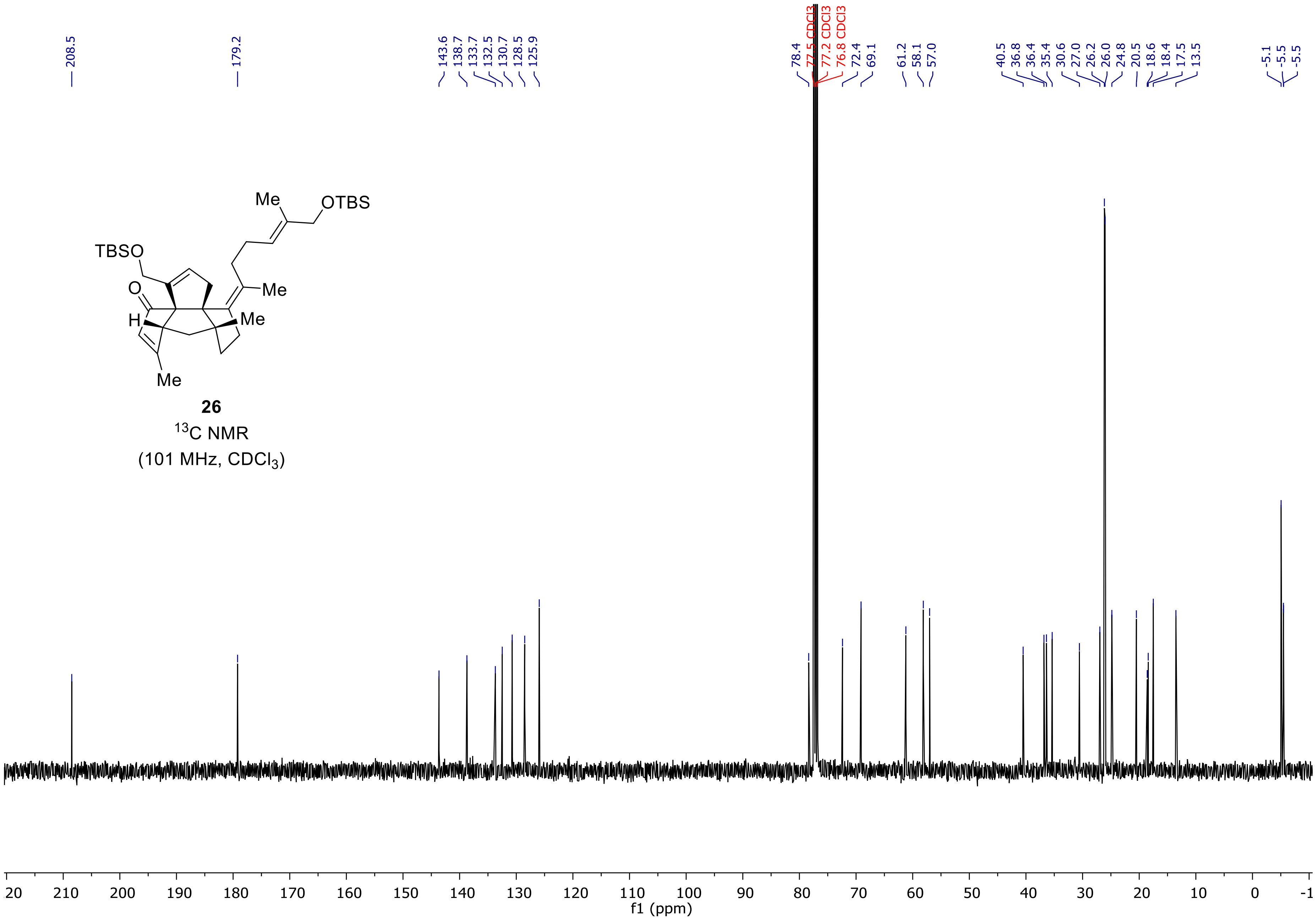
208.5
179.2
143.6
138.7
133.7
132.5
130.7
128.5
125.9
78.4
77.5 CDCl3
77.2 CDCl3
76.8 CDCl3
72.4
69.1
61.2
58.1
57.0
40.5
36.8
36.4
35.4
30.6
27.0
26.2
26.0
24.8
20.5
18.6
18.4
17.5
13.5
-5.1
-5.5
-5.5
Me
OTBS
TBSO
O
H
Me
Me
Me
26
^{13}C NMR
(101 MHz, $CDCl_3$)
f1 (ppm)

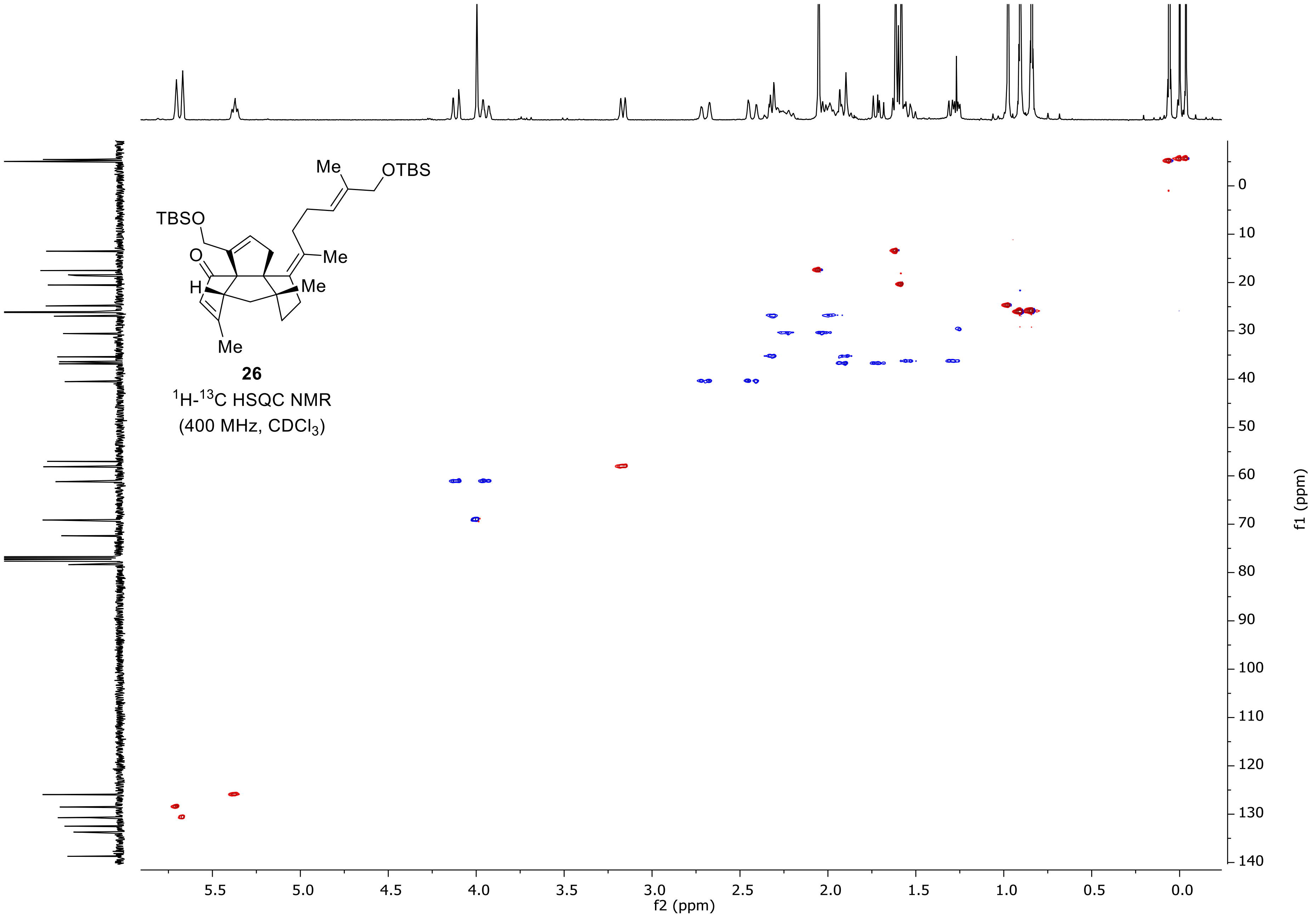
Me
OTBS
TBSO
O
H
Me
Me
Me
26
^{1}H-^{13}C HSQC NMR
(400 MHz, $CDCl_3$)
f2 (ppm)
f1 (ppm)

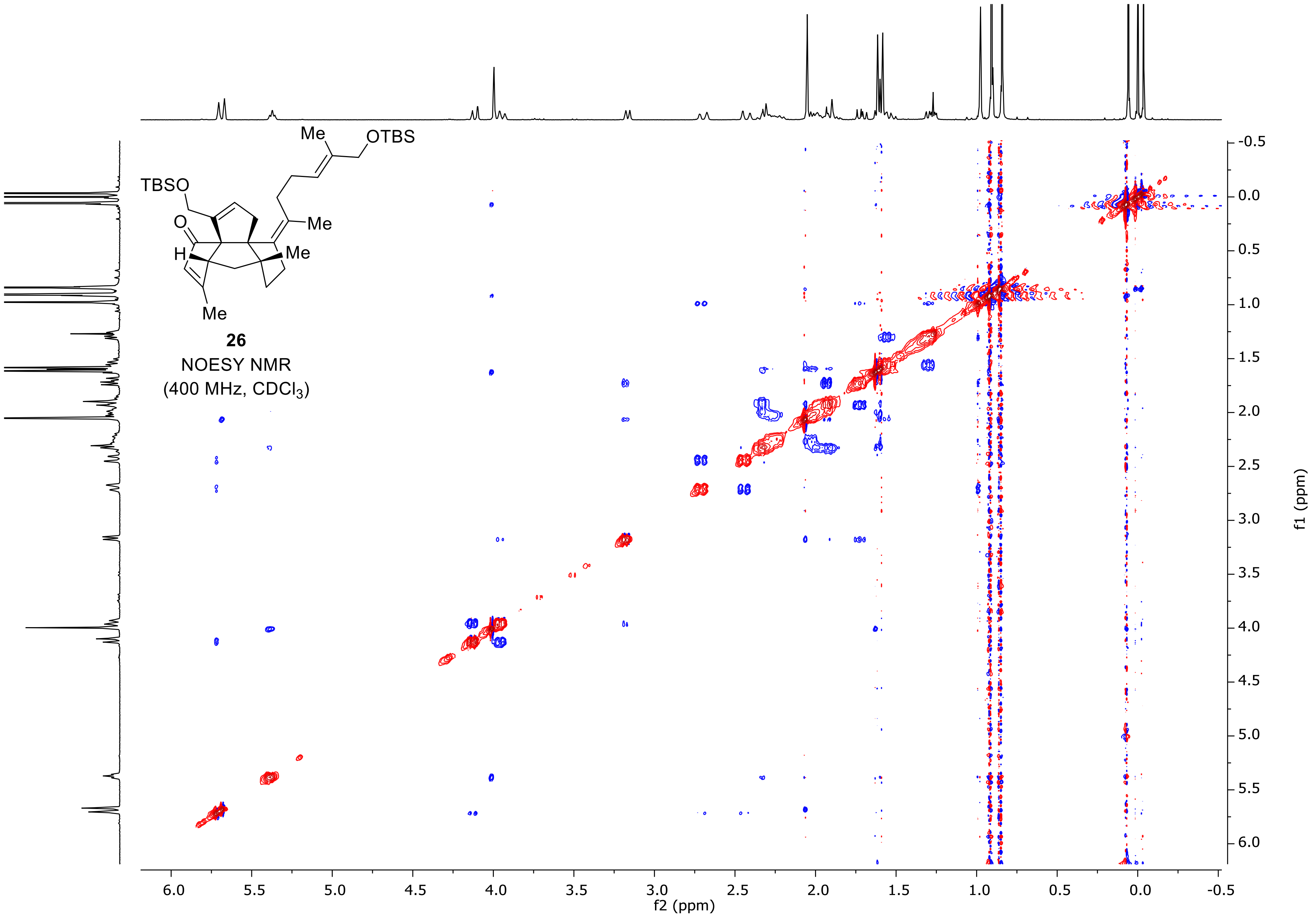
Me
OTBS
TBSO
O
Me
H
Me
Me
26
NOESY NMR
(400 MHz, $CDCl_3$)
f2 (ppm)
f1 (ppm)
6.0
5.5
5.0
4.5
4.0
3.5
3.0
2.5
2.0
1.5
1.0
0.5
0.0
-0.5

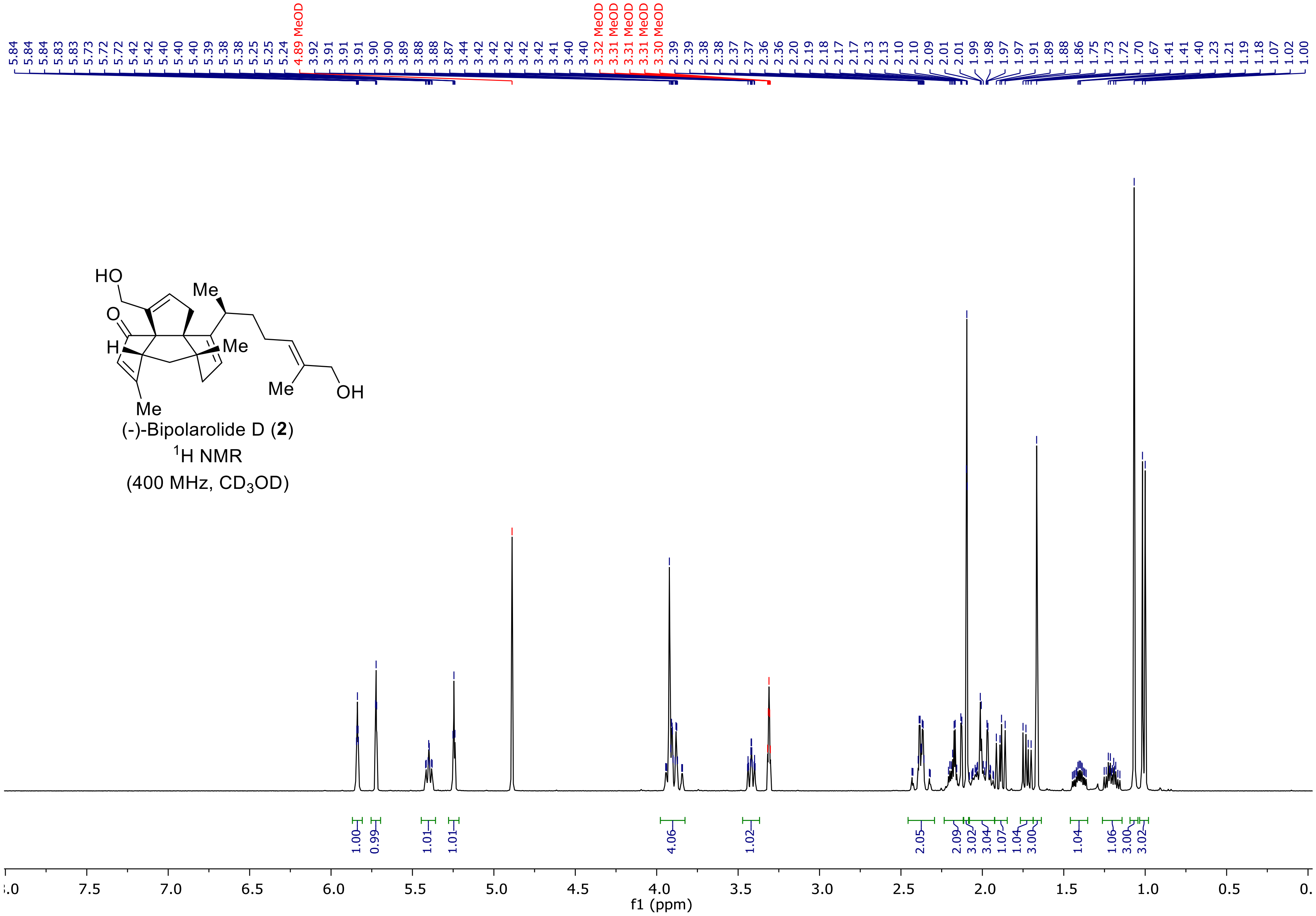
(-)-Bipolarolide D (**2**)
^{1}H NMR
(400 MHz, CD_3OD)
f1 (ppm)

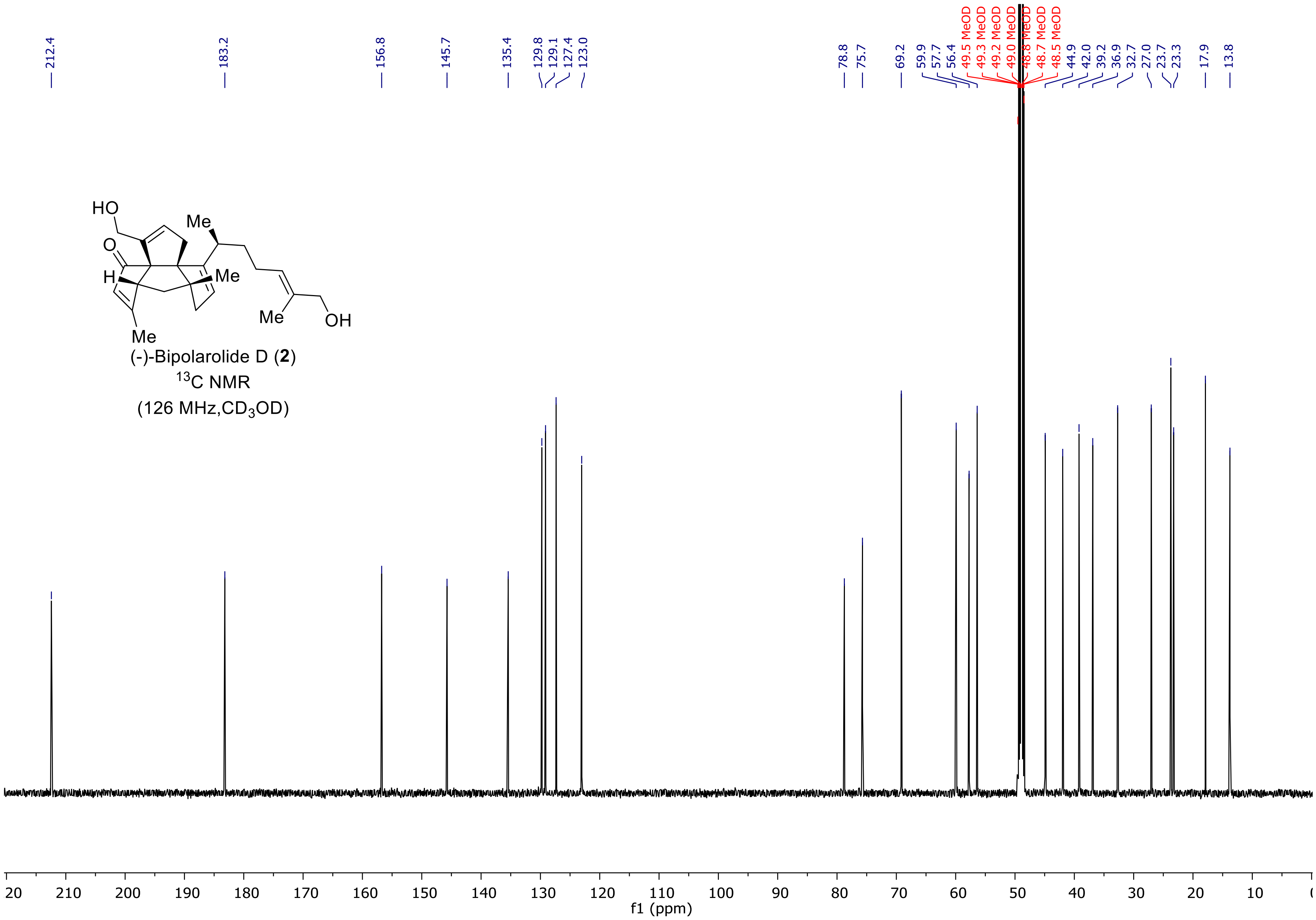
HO
O
Me
H
Me
Me
Me
OH
(-)-Bipolarolide D (2)
13C NMR
(126 MHz,CD3OD)
212.4
183.2
156.8
145.7
135.4
129.8
129.1
127.4
123.0
78.8
75.7
69.2
59.9
57.7
56.4
49.5 MeOD
49.3 MeOD
49.2 MeOD
49.0 MeOD
48.8 MeOD
48.7 MeOD
48.5 MeOD
44.9
42.0
39.2
36.9
32.7
27.0
23.7
23.3
17.9
13.8
f1 (ppm)

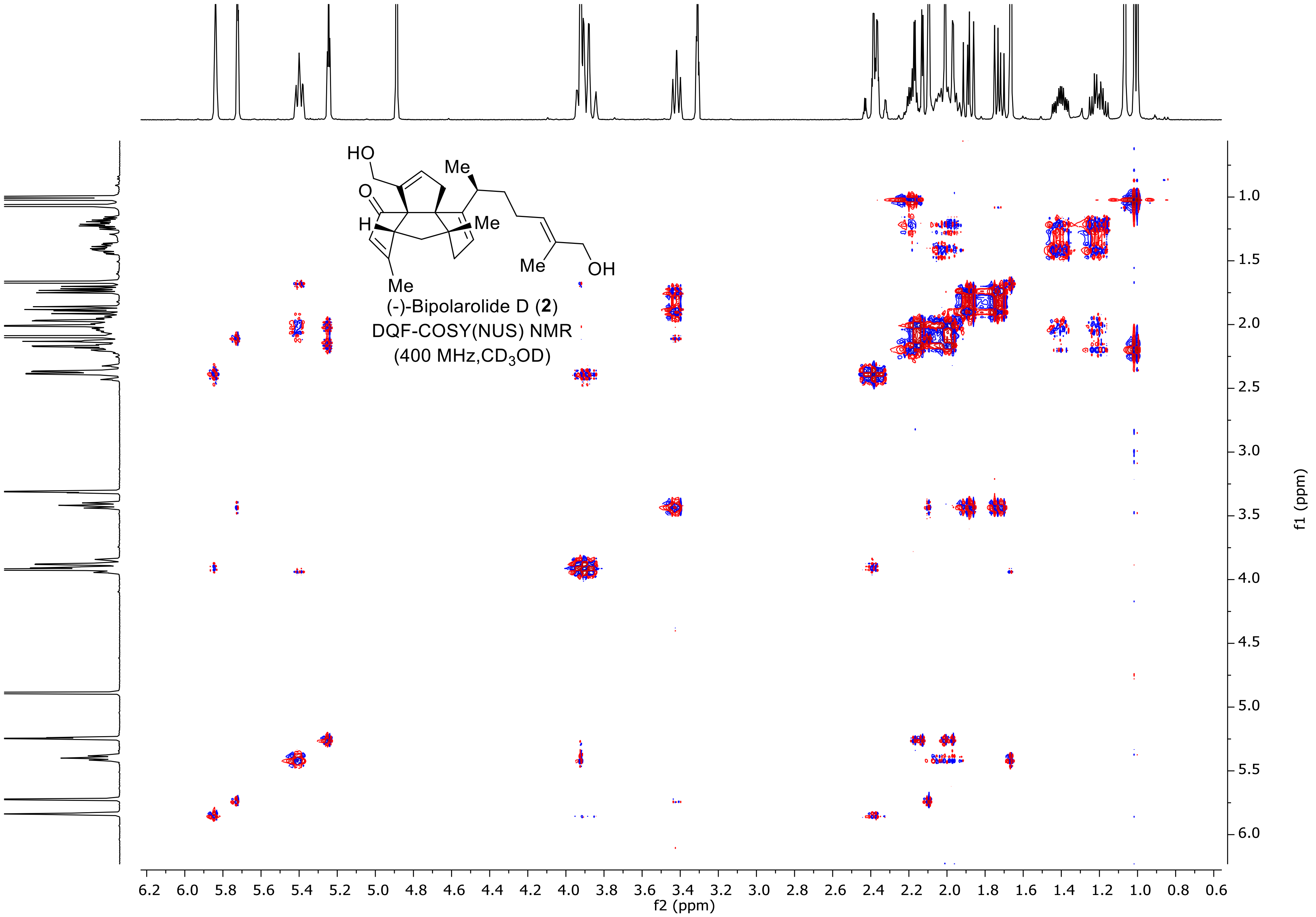
HO
Me
O
H
Me
Me
OH
Me
(-)-Bipolarolide D (2)
DQF-COSY(NUS) NMR
(400 MHz,CD3OD)
f2 (ppm)
f1 (ppm)

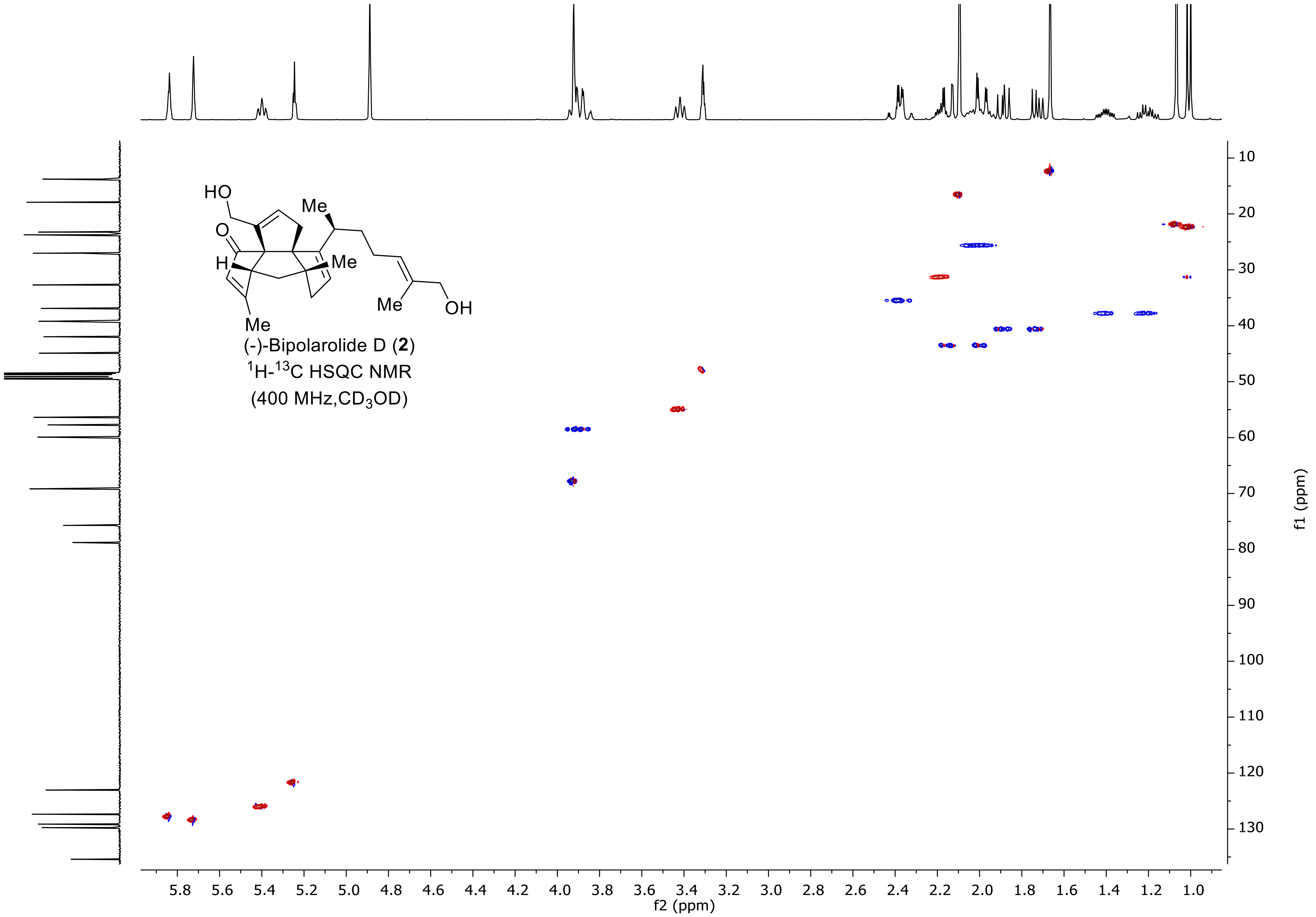
HO
O
H
Me
Me
Me
Me
OH
(-)-Bipolarolide D (2)
1H-13C HSQC NMR
(400 MHz,CD3OD)
f2 (ppm)
f1 (ppm)
